# The Good, the Bad, and the Ugly: “HiPen”, a New Dataset for Validating (S)QM/MM Free Energy Simulations

**DOI:** 10.3390/molecules24040681

**Published:** 2019-02-14

**Authors:** Fiona L. Kearns, Luke Warrensford, Stefan Boresch, H. Lee Woodcock

**Affiliations:** 1 Department of Chemistry, University of South Florida, 4202 E. Fowler Avenue, Tampa, FL 33620, USA; fionakearns@mail.usf.edu (F.L.K.); lwarrensford@mail.usf.edu (L.W.); 2 Department of Computational Biological Chemistry, Faculty of Chemistry, University of Vienna, Waehringerstrasse 17, A-1090 Vienna, Austria

**Keywords:** indirect free energy simulations, quantum mechanical molecular mechanical hybrid modeling, free energy perturbation, nonequilibrium work simulations, Bennett’s acceptance ratio, Jarzynski’s equation, Crooks’ equation

## Abstract

Indirect (S)QM/MM free energy simulations (FES) are vital to efficiently incorporating sufficient sampling and accurate (QM) energetic evaluations when estimating free energies of practical/experimental interest. Connecting between levels of theory, i.e., calculating $\Delta A^{low\to high}$, remains to be the most challenging step within an indirect FES protocol. To improve calculations of $\Delta A^{low\to high}$, we must: (1) compare the performance of all FES methods currently available; and (2) compile and maintain datasets of $\Delta A^{low\to high}$ calculated for a wide-variety of molecules so that future practitioners may replicate or improve upon the current state-of-the-art. Towards these two aims, we introduce a new dataset, “HiPen”, which tabulates $\Delta A_{gas}^{MM\to 3ob}$ (the free energy associated with switching from an MM to an SCC−DFTB molecular description using the 3*ob* parameter set in gas phase), calculated for 22 drug-like small molecules. We compare the calculation of this value using free energy perturbation, Bennett’s acceptance ratio, Jarzynski’s equation, and Crooks’ equation. We also predict the reliability of each calculated $\Delta A_{gas}^{MM\to 3ob}$ by evaluating several convergence criteria including sample size hysteresis, overlap statistics, and bias metric (Π). Within the total dataset, three distinct categories of molecules emerge: the “good” molecules, for which we can obtain converged $\Delta A_{gas}^{MM\to 3ob}$ using Jarzynski’s equation; “bad” molecules which require Crooks’ equation to obtain a converged $\Delta A_{gas}^{MM\to 3ob}$; and “ugly” molecules for which we cannot obtain reliably converged $\Delta A_{gas}^{MM\to 3ob}$ with either Jarzynski’s or Crooks’ equations. We discuss, in depth, results from several example molecules in each of these categories and describe how dihedral discrepancies between levels of theory cause convergence failures even for these gas phase free energy simulations.

## 1. Introduction

Calculating accurate free energy differences from simulation involves two disparate requirements: accurate energetic evaluations (e.g., semi-empirical quantum mechanics (SQM) or high level electronic structure methods (QM)) and sufficiently long simulations to appropriately sample relevant regions of conformational space. (We use abbreviations to reference semiempirical quantum mechanical (SQM) methods, quantum mechanical (QM) methods such as DFT or ab initio, and molecular mechanical (MM) methods. We also use the abbreviations “QM/MM” to refer to quantum mechanical/molecular mechanical hybrid modeling, “SQM/MM” to refer to semi-empirical quantum mechanical/molecular mechanical hybrid modeling, and “(S)QM/MM” to refer to either QM/MM or SQM/MM). Of course, one can see the incongruity here: extensive simulations do not lend themselves to the time and resource intensive nature of (S)QM energy/force evaluations. As such, there is a large effort in the free energy simulation (FES) field to take advantage of both the efficiency of force-field based simulations, and accuracy of (S)QM energetics [1,2,3,4,5,6,7] Thus, we are marching toward the goal of efficiently calculating free energies at the (S)QM/MM level of theory ($\Delta A^{(S)QM/MM}$), but our destination is still far on the horizon.

**Figure 1:**
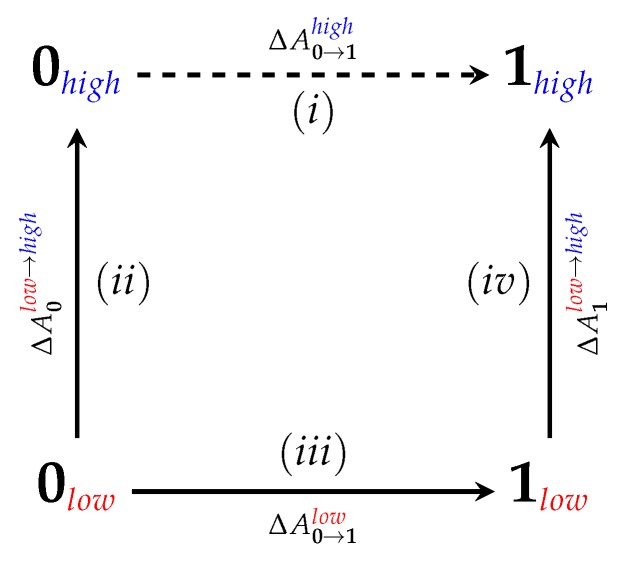
The indirect cycle underlying (S)QM/MM FES. “0” and “1” denote the two physical end states, e.g., a molecule in gas phase and solution, or a ligand in the free state and bound to a receptor.

A useful “trick” to combine the accuracy of (S)QM/MM levels of theory and the extent of sampling only reachable using MM force fields is to carry out the (S)QM/MM FES *indirectly* [8,9,10,11,12,13]. Since $\Delta A_{0\to 1}^{(S)QM/MM}$, a free energy difference of interest “0→1” at the desired high (we use the term “low” to refer to any level of theory capable of conducting sufficient sampling, while the term “high” to refer to any level of theory that is too computationally expensive to conduct sufficient sampling for FES but that provides accurate energetics for evaluating FES) level of theory, is a state function, it can be calculated by employing the thermodynamic cycle shown in Figure 1. Specifically,

(1) $$\Delta A_{0\to 1}^{high}=-\Delta A_{0}^{low\to high}+\Delta A_{0\to 1}^{low}+\Delta A_{1}^{low\to high}.$$

Here, $\Delta A_{0\to 1}^{low}$ is a standard FES at the low level of theory, and the steps $\Delta A_{0}^{low\to high}$ and $\Delta A_{1}^{low\to high}$ connect the low level back to the high level of interest. The low level is chosen such that sufficient sampling can be conducted. Further, if a force field is used for the low level, several tricks of the trade, e.g., soft-core potentials [14,15], may help facilitate alchemical transformations [2]. In particular, if $\Delta A_{0\to 1}^{low}$ is carried out at the MM level of theory, any standard free energy estimator can be used for its calculation, such as free energy perturbation (FEP) [16], thermodynamic integration (TI) [17], Bennett’s Acceptance Ratio (BAR) [18], its multi-state extension MBAR [19], vFEP [20], WHAM [21], etc.

Correction legs (ii) and (iv) have traditionally been calculated using FEP, Equation (2), written here for the specific application of connecting the low and high levels of theory:

(2) $$\Delta A^{low\to high}=-k_{b}T\ln{\langle \exp\left(\frac{-\Delta U^{low\to high}}{k_{b}T}\right)\rangle }_{low}$$

Here, $k_{B}$ and *T* have the usual meaning of Boltzmann’s constant and temperature, and ${\langle \ldots \rangle }_{low}$ denotes an ensemble average generated at the low level of theory and in the canonical ensemble. This is, of course, highly advantageous as costly simulations at the high level of theory are not needed. In a post-processing step, the difference $\Delta U^{low\to high}=U^{high}-U^{low}$ is computed for every frame saved during the *low* level simulation. For this reason, FEP is a so-called “one-sided” method as it only requires simulation from “one side” of the free energy difference.

Although Equation (2) is formally exact, recent research has shown conclusively that FEP, when applied to the calculation of $\Delta A^{low\to high}$, rarely gives converged free energy differences [22,23,24,25,26,27,28,29]. In fact, by now few would dispute the statement that FEP *cannot* be used to compute $\Delta A^{low\to high}$ for systems of practical interest. In order for Equation (2) to converge in practice, at least some configurations sampled at the low level of theory also need to be low energy configurations at the high level of theory (cf. [30]). Typically, however, there are disparities in “stiff” (bonds and angles) and “soft” (dihedrals) degrees of freedom between low (MM or SQM) and high (SQM or QM) levels of theory. Simply put, in many cases, an “MM” molecule does not look like a “QM” molecule, and slight differences between these structures can result in drastic convergence errors [22,23,24,25,26,27,28]. These disparities are exacerbated as the size of the high level/QM region increases. More generally, in most cases, the phase space sampled at the low level of theory has little or no overlap with the phase space which would be sampled if the system of interest were treated at a high level of theory. Following, e.g., Pohorille et al. [30], phase space overlap can be quantified by comparing the distribution of forward (low→high) energy differences $p(\Delta U_{fw})$ and (negative) backward (high→low) energy differences $p(-\Delta U_{bw})$ (cf. below) (For example, $p(\Delta U_{fw})$ is the histogram obtained from configurations saved during a simulation at the low level of theory, for which one computes $\Delta U_{fw}=U^{high}-U^{low}$. The availability of $p(-\Delta U_{bw})$, of course, depends whether simulations at the high level of theory could be carried out).

The failure of FEP would suggest using more efficient methods to compute $\Delta A^{low\to high}$; however, in the present context (as compared to regular alchemical FES), the choice is severely limited. Consider, e.g., Bennett’s Acceptance Ratio (BAR, Equation (3)), a widely used method for calculating ΔA’s [31]. If used to evaluate $\Delta A^{low\to high}$ as in (ii) or (iv), it takes the form:

(3) $$\Delta A^{low\to high}=k_{b}T\ln\left(\frac{{\langle f(U^{low}-U^{high}+C)\rangle }_{high}}{{\langle f(U^{high}-U^{low}-C)\rangle }_{low}}\right)+C,$$

where $f\left(x\right)={\left(1+\exp\left(x/k_{b}T\right)\right)}^{-1}$ and

(4) $$C=k_{b}T\ln\frac{Q_{0}N_{1}}{Q_{1}N_{0}}.$$

As one sees from the use of ${\langle \ldots \rangle }_{low}$ and ${\langle \ldots \rangle }_{high}$ in Equation (3), BAR requires simulation at both endstates, thus is a so-called “two-sided” method. The need for simulations at both levels of theory makes the use of BAR problematic, i.e., computationally too expensive. Thus, the search for reliable, less expensive methods to compute correction steps between low and high levels of theory continues. We, and many others in the FES community, have explored many potential solutions including reweighting schemes [23], force-matching techniques [32], and nonequilibrium work methods [33,34].

Given the underlying problem, poor or non-existing overlap between phase space sampled at the low and high level of theory, there are two paths towards reliably calculating $\Delta A^{low\to high}$. One possible path is to enhance efficiency of methods used to compute free energy differences between disparate levels of theory. Another possible path may be to make the low level of theory “look” more similar in configurational space to the high level of theory. Our proof-of-concept results using force-matching in Reference [32], as well as the results of other groups (e.g., [35,36,37,38,39,40,41,42,43,44,45,46,47,48,49,50]), are examples of the latter strategy. Concerning the former, we successfully used nonequilibrium work techniques to compute $\Delta A^{low\to high}$ [33,34]. In particular, we explored the utility of Jarzynski’s (JAR) equation [51], the nonequilibrium analog to FEP. Formally, one replaces the energy difference $\Delta U^{low\to high}$ in Equation (2) by the nonequilibrium work $W^{low\to high}$ needed to bring the system from a low to high level description:

(5) $$\Delta A\left(low\to high\right)=-k_{b}T\ln{\langle \exp\left(\frac{-W^{low\to high}}{k_{b}T}\right)\rangle }_{low}$$

Although the nonequilibrium switching simulations needed to obtain $W^{low\to high}$ do require evaluation of high level energies and forces at every step, two factors make such calculations practicable. First, at least in our tests to date, rather short switching simulations (a few hundred to a few thousand MD steps per switch) were sufficient. Second, these switching simulations are a post-processing step started from coordinate/velocity sets saved during equilibrium simulations at the low level of theory (cf. Materials and Methods). Therefore, they can be run in parallel, making the JAR calculation scheme much more computationally efficient than conducting a sufficiently long high level simulation, e.g., as needed for BAR.

Given that FEP cannot be used to calculate free energy differences $\Delta A^{low\to high}$ reliably, another challenge in benchmarking performance of FES estimators such as FEP and JAR is obtaining reference results. In the past, we have used BAR, as well as its nonequilibrium analog the Crooks’ equation (CRO) [52], to generate reference results [33,34]. As with FEP and JAR, energy differences in BAR are formally replaced by nonequilibrium work values to give CRO, i.e.,

(6) $$\Delta A\left(low\to high\right)=k_{b}T\ln\left(\frac{{\langle f(W^{high\to low}+C)\rangle }_{high}}{{\langle f(W^{low\to high}-C)\rangle }_{low}}\right)+C$$

Of all the methods discussed, CRO is the most computationally expensive. As with BAR, one must conduct long equilibrium simulations at both levels of theory and then conduct nonequilibrium switching simulations, this time launched not only from low→high but also from high→low. In real applications CRO, and most likely BAR, are far too expensive to calculate $\Delta A^{low\to high}$ within an indirect scheme of practical interest; however, in the context of methodological work, they provide a means to obtain reference results for comparison to cheaper methods.

**Figure 2:**
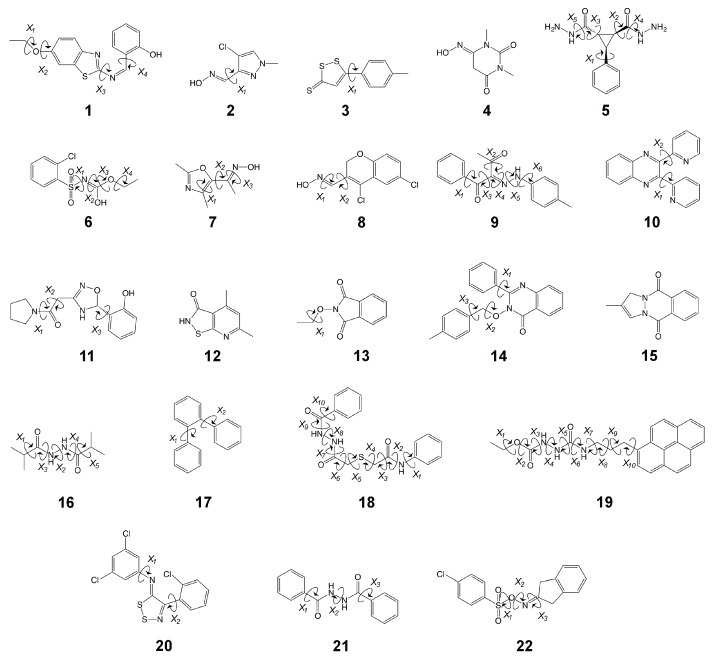
The HiPen dataset modeled herein. Dihedrals that were probed or randomized (see Methods) in this work have been identified for each molecule.

While our earlier work has demonstrated the utility of both nonequilibrium work methods, in particular JAR [33,34], and force-matching approaches [32] for the computation of $\Delta A^{low\to high}$, the techniques were tested only on a relatively small number of systems. To advance the state of the art, a broader test of the existing methodology is required and is the subject of the current study. In previous unrelated work [53], we used ParamChem (https://cgenff.umaryland.edu, a web-interface for automatically predicting parameter and topology sets for small molecules [54,55]) to obtain CHARMM generalized force-field (CGenFF) parameters [56] for the Maybridge Hitfinder set [57]. As part of the ParamChem procedure, “penalties” are assigned that indicate the expected quality of generated parameters. From the full Maybridge set, we then selected 22 molecules that: (1) represent chemical diversity seen in medicinal chemistry, as the Maybridge set includes molecules that are drug-like according to Lipinski’s rule of 5; and (2) had high penalties for bonded and/or charge parameters. We expect these systems, shown in Figure 2, to be challenging cases when computing $\Delta A^{low\to high}$. Because of the high parameter penalties, we refer to our chosen set as “HiPen”.

Given the diversity of the compounds chosen, we view our HiPen set as a benchmark set that can be used to compare methods for computing $\Delta A^{low\to high}$ in the context of indirect (S)QM/MM FES. In related areas of computational chemistry, extensive benchmark sets have proven very useful. For example, the Benchmark Energy and Geometries Database (BEGDB, http://www.begdb.com) is a highly utilized, online computational resource where quantum quality energies and properties for a wide variety of molecules are deposited. BEGDB has a stated purpose of “serv[ing] as benchmarks for testing and parameterization of other computational methods.” Datasets maintained in BEGDB have been cited ≈2400 times, with some of the more frequently cited datasets being S22 (1079 citations), and S66 (454 citations). Similarly, the Minnesota Databases 2.0 (MN2.0) are a large collection of datasets for comprehensive validation. Many of the works citing these BEGDB and MN2.0 datasets are methodological investigations aiming to develop, improve, or validate, the performance of Density Functional Theory (DFT). For example, several DFT functionals that have recently (since 2015) been derived, validated, or improved upon using BEGDB or MN2.0 datasets include but are certainly not limited to: B97M-V [58], the occ-RI-K algorithm [59], ωB97M-V [60], minimally adaptive basis (MAB) [61], ωB97M(2) [62], revised M06 (revM06) [63], revised M06-L [64], and MN15 [65], as well as several large review-style validation studies of DFT methods in general [66,67]. Additionally, the MN2.0 databases were used by Peverati and Truhlar to search for a “universal” density functional in 2014 [68]. For good measure, we also list these seminal works in the DFT development field [69,70,71,72,73]. (It should be noted these references by no means represent a complete list of all DFT improvements facilitated by BEGDB and MN2.0. For a more complete literary listing, travel to http://www.begdb.com and https://comp.chem.umn.edu/db/ and follow links to each dataset’s debut publications, and then view all citing publications.) Thus, as online repositories of maintained datasets, BEGDB and MN2.0 represent invaluable resources to the quantum chemistry modeling discipline and in turn chemistry at large.

In the area of force-field focused alchemical FES, benchmarks and comparative tests exist as well; e.g., a study comparing results for relative free energy differences obtained with the most widely used programs was just published [74]. The datasets forming the basis for the various SAMPL competitions are another excellent source of curated experimental reference values [75,76,77]. Several test systems can be downloaded from the “alchemistry.org” web site (see https://www.alchemistry.org, follow the link “Test System Repository”). Since nothing comparable to these alchemical collections or to aforementioned QM benchmark sets yet exists for indirect (S)QM/MM FES, we view the HiPen set (Figure 2) as the core of a benchmark in this area. Studies with a related goal include work by Cave-Ayland et al. [25], in which forward $p(\Delta U_{fw})$ and backward energy distributions $p(-\Delta U_{bw})$ for a number of compounds are computed and compared systematically, as well as the work in Reference [29], where Ryde searched for criteria to determine whether $\Delta A^{low\to high}$ values have converged or not. Clearly, it should be useful to have available reference results against which novel methodological developments can be compared. The present study is the beginning of such a database, and we ask others in the field to join us.

In Reference [34], we discerned three overall factors contributing to the difficulty of obtaining converged results: (i) subtle differences in bond lengths and angles (i.e., the “stiff” degrees of freedom); (ii) different conformational preferences, such as preferred ranges for dihedrals (i.e., “soft” degrees of freedom); and (iii) differences in charge distribution of the region either described by the low or the high level of theory. The last complication arises only in aqueous solution or in a protein–ligand complex. Here, we concentrate on the first two complications, mismatches in stiff and soft degrees of freedom; hence, all calculations here are carried out in the gas phase. Further, in the present work, we use the MM force field as the low level “as is”, i.e., we do not attempt to improve phase space overlap through force-matching or related techniques. Additionally, for the purposes of generating reference results via two-sided methods such as BAR and CRO, we have chosen to utilize a semi-empirical method as our high level of theory, as it is still cheap enough to achieve relatively efficient sampling. Specifically, we are interested in whether, at least for some systems, FEP is enough to compute $\Delta A_{gas}^{low\to high}$, whether JAR with short switching protocols is sufficient for converged results, or whether two-sided methods, which are too expensive for general use, are needed.

In addition to reporting $\Delta A_{gas}^{MM\to 3ob}$ obtained with each of these methods, we report criteria we have in the past found useful to identifying failures in “convergence” (obtaining the correct $\Delta A^{low\to high}$ within reasonable certainty). These include comparing differences in magnitude of “forward” and “backward” ΔA’s (i.e., $\Delta A^{MM\to 3ob}$ vs. $-\Delta A^{3ob\to MM}$) [23], calculating “sample size hysteresis” (Equation (10) in Reference [34]), calculating the standard deviation in $\Delta U^{low\to high}$/$W^{low\to high}$ and vice versa [78] (cf. [29]), calculating distribution overlaps in $\Delta U^{low\to high}$ and $W^{low\to high}$ values [7,32,33,34,78], and finally applying the Π criterion introduced by Wu and Kofke [79] to the forward/backward energy distributions or work distributions [32,78]. To understand difficult cases and failures, we also computed distributions of dihedral angles sampled during simulations at the two levels of theory, as well as at the end of forward and backward switching simulations [23,33,34].

## 2. Results

Given the average energy difference between an MM and 3ob calculation differs on the order of 10${}^{5}$ kcal/mol, $\Delta A_{gas}^{MM\to 3ob}$ by default can be quite large. Thus, even large standard deviations, $\sigma (\Delta A_{gas}^{MM\to 3ob})$, of 10 kcal/mol or even more are easy to overlook. Therefore, we list “offset” values in Table 1, which must be added for reported positive ΔA and subtracted for reported negative ΔA to give the actual total $\Delta A_{gas}^{MM\to 3ob}$ (for values <0 in coming tables, the offset should be subtracted to give the true ΔA, for values >0 in coming tables, the offset should be added to give the true ΔA). Extracting “offset” values allows trends in variance and convergence failure to be more apparent, and allows data to be presented more compactly.

Additionally, in Table 1, we list the bonded and charge parameter penalties reported for the CGENFF parameters by ParamChem as well as the number of atoms (total/non-hydrogen) in each selected molecule. As described in the Introduction, we specifically chose the twenty-two molecules based on high parameter penalties, in addition to picking molecules to represent chemical diversity seen in small molecule drug sets. Table 2 and Table 3 provide all calculated $\Delta A_{gas}^{MM\to 3ob}$ results for each of the 22 HiPen molecules. In addition, we report values for the convergence criteria considered: hysteresis (Hyst) calculated by comparing ΔA calculated from the complete dataset to block averaged ΔA (this criterion is a simplified version of considerations by Woods and co-workers [80]) (see [78] for complete definition of our “hysteresis” criterion), standard deviations of ΔA calculated from 10 blocks, average $\bar{\Delta U}$ or average $\bar{W}$, standard deviation of ΔU/*W* to indicate width of input distributions (the average energy differences/work values and their standard deviations could also be used to estimate ΔA according to the second order cumulant expansion; however, the underlying distributions are far from Gaussian), and percentage overlap of “forward” and “backward” distributions.

Another criterion we found useful to predict whether free energy differences obtained from free energy estimators FEP, BAR, JAR, and CRO are likely to be converged [32,78] is Π, introduced by Wu and Kofke [79]. Π provides a quantitative means for determining if a distribution was collected from a sufficiently large sample in a manner free of bias. A complete discussion of the Π criterion in theory and derivation is beyond the scope of this work, but we strongly advise the reader to see Wu and Kofke’s works in 2004 and 2005 introducing this measure and its uses [79,81]. In Reference [32], Equation (3) gives the one-sided Π criterion, which we used to determine “reliability” of distributions used herein. Π should be >0.5 to indicate a “well behaved” distribution of $\Delta U^{low\to high}$ or $W^{low\to high}$. It should be noted, however, that the Π criterion assumes input energy or work distributions obey at least an approximate Gaussian distribution, which is not necessarily likely for molecules of practical interest, and as we show below, is not always the case in HiPen. Nevertheless, we have still found reasonable correlation between Π values and deviations from reference results in Reference [78].

In Reference [78], we also noted that FEP/JAR is likely to fail when the standard deviation of energy difference or work values ($\sigma_{\Delta U^{low\to high}}$ or $\sigma_{W^{low\to high}}$), i.e., the raw data entering Equation (2) or Equation (5) becomes too large; $\sigma >4\;k_{B}T$ being the absolute threshold of reliability, beyond which the corresponding ΔA is likely untrustworthy. A similar observation in the context of indirect (S)QM/MM FES was recently also made by Ryde [29]. All of these criteria have proven useful in the past to indicate convergence of $\Delta A^{low\to high}$, as well as while investigating convergence failure in more difficult cases. As such, for each $\Delta A^{MM\to 3ob}$ presented in this work, we also calculated each of these metrics to evaluate the “quality of convergence”.

## 3. Discussion

Looking at Table 2 and Table 3, one immediate observation is that equilibrium methods (FEP/BAR) overall provided results we would consider “unreliable.” This can be seen in Table 2: for most molecules, FEP (fw), FEP (bw), and BAR results differ by several kcal/mol; most ΔU distributions are broad (with $\sigma_{\Delta U}\gg 4k_{B}T$) and do not pass the Π sampling criterion requirement of being >0.5; calculated Hyst values are >1 kcal/mol; and finally the percentage overlap in nearly all cases is not large enough to ensure sufficient sampling for even the two-sided estimator BAR. Therefore, we did not see fit to classify molecules according to performance of FEP/BAR, and results obtained by equilibrium methods are not considered further. While compiling nonequilibrium results, three clusters of molecules emerged within HiPen, which we have named the Good, the Bad, and the Ugly. “Good” molecules were any molecules for which a converged $\Delta A_{gas}^{MM\to 3ob}$ could be calculated using JAR (fw). “Bad” molecules were any molecules for which JAR (fw)/JAR (bw) calculations appeared unconverged, but for which CRO was (largely) converged. “Ugly” molecules were any molecules for which even CRO exhibited convergence issues according to all of our convergence metrics. It is worth expounding upon some examples in each of Good, Bad, and Ugly cases and illustrate how nonequilibrium switches can be used to sharpen distributions and improve configurational overlap between levels of theory in most of these cases. Although we have collected ΔU/*W* distribution overlaps as well as rotatable dihedral distributions for every molecule in the HiPen data set, presenting all such data would overwhelm the reader, and represent somewhat of a digression from the purpose of this work. For those looking to validate their own data, all plots can be found in the Supplementary Materials. Additionally, all data including input scripts will be made available upon request, and we have compiled all minimized topology/coordinates as well as parameters and input files and those can be found at https://zenodo.org/record/2328952 (doi:10.5281/zenodo.2328952). Before we begin the classification and discussion, we would like to emphasize that our classifications of “Good”, “Bad”, and “Ugly” are merely intended for grouping comparable results and attempting to describe trends from those groups without having to describe results from every molecule. Within each group, there are further gradients of “best” and “worst” convergence performers.

### 3.1. The Good

We were able to calculate $\Delta A_{gas}^{MM\to 3ob}$ using our JAR (fw) protocol (a 1 ps “forward” switching protocol, see Methods for details) for the following molecules: **2**, **3**, **4**, **7**, **10**, **11**, **12**, **13**, **14**, **15**, and **17**. Thus, for these molecules obtaining $\Delta A^{low\to high}$ within an indirect scheme should be fairly straightforward and should not require full simulations at the high level of theory. We will focus our discussion here on molecules **2** and **11**.

#### 3.1.1. Molecule **2**

Molecule **2** (4-chloro-1-methyl-1*H*-pyrazole-3-carbaldehyde oxime, ZINC00077329), is a pyrazole oxime compound, which can be used to synthesize other pyrazole oximes that have antitumor, insecticidal, and acaricidal activities [82].

**Figure 3:**
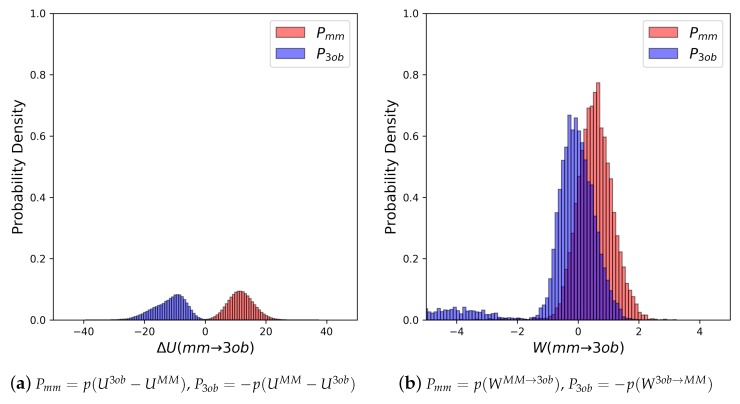
(**a**) Molecule **2**’s potential energy “forward” ($P_{mm}$) and “backward” ($P_{3ob}$) distributions plotted as “offset” from the $\bar{\Delta U^{MM\to 3ob}}$ to simplify the x-axis. (**b**) Molecule **2**’s nonequilibrium work “forward” ($P_{mm}$) and “backward” ($P_{3ob}$) distributions plotted as “offset” from the $\bar{W^{MM\to 3ob}}$ to simplify the x-axis.

The potential energy overlaps, shown in Figure 3a, indicate $P_{mm}$ and $P_{3ob}$ are quite distinct, with ${\langle \Delta U^{MM\to 3ob}\rangle }_{MM}-{\langle -\Delta U^{3ob\to MM}\rangle }_{3ob}$, i.e., the difference between distribution means, being 23.62 kcal/mol, while the standard deviation of these distributions is 4.29 and 5.28 kcal/mol, respectively; resulting in an overlap (area under intersecting $P_{mm}$ and $P_{3ob}$ distributions) of only 0.29%. (We use $P_{mm}$ here to refer to the probability density, from an MM simulation, of $p(U^{3ob}-U^{MM})$ or $p(W^{MM\to 3ob})$, respectively. We use $P_{3ob}$ to refer to the probability density $p(-\left(U^{MM}-U^{3ob}\right))$ or $p(-W^{3ob\to MM})$, respectively, obtained from a 3ob simulation) Further, FEP (fw) and FEP (bw) results are not equal in magnitude, i.e., −255.52 kcal/mol vs. 258.88 kcal/mol, and are clearly problematic as shown by the one-sided Π values of −1.15 and −1.47, for FEP (fw) and FEP (bw), respectively. Thus, potential energy distributions from equilibrium simulations are not “well behaved” for **2**, and using FEP or BAR to calculate $\Delta A_{gas}^{MM\to 3ob}$ is not likely to provide converged or accurate results. However, utilizing nonequilibrium $W^{MM\to 3ob}$ instead of $\Delta U^{MM\to 3ob}$ vastly improves overlap of $P_{mm}$ and $P_{3ob}$ (shown in Figure 3b) to 53.89%, and improves one-sided Π values to 1.86 and 2.19 for JAR (fw) and JAR (bw), respectively. One point of concern regarding *W* distributions for **2** is the “tail”, or small secondary peak, seen around −4 kcal/mol in $P_{3ob}$; however, despite this secondary peak, there appears to be more than enough configurational overlap to provide converged results.

It should also be noted how poorly FEP performs compared to converged results from JAR and CRO: FEP (fw) predicts $\Delta A_{gas}^{MM\to 3ob}$ = −255.52 kcal/mol, while CRO predicts $\Delta A_{gas}^{MM\to 3ob}$ = −271.84 kcal/mol, a difference of 16.32 kcal/mol. This FEP/CRO discrepancy is quite concerning as these are gas phase FES, and thus the only potential sources for error in $\Delta A_{gas}^{MM\to 3ob}$ are due to lack of overlap in intramolecular degrees of freedom. In aqueous solution, or in simulations of a protein–ligand complex, errors resulting from the use of FEP are likely to be even larger. Further, it would be foolish to hope for error cancellation in steps $\Delta A_{0}^{low\to high}$ and $\Delta A_{1}^{low\to high}$ of the full indirect scheme in Figure 1, as the two corrections may be computed in different environments. This highlights the need for improving overlap/convergence when computing free energy differences between levels of theory.

#### 3.1.2. Molecule **11**

Molecule **11** (ZINC00138607, IUPAC name 2-[5-(2-hydroxyphenyl)-4,5-dihydro1,2,4-oxadiazol-3-yl]1-tetrahydro-1*H*-pyrrol-1-ylethan-1-one) contains chemical features seen in a large number of molecules within the PubChem database. Thus, correctly modeling such a molecule is imperative for providing theoretical aid to experimentalists applying some of **11**’s chemical features in drug discovery or other application-driven investigations.

**Figure 4:**
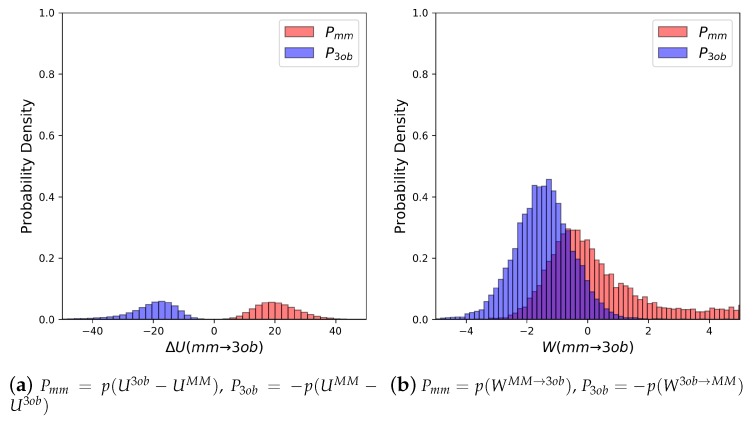
(**a**) Molecule **11**’s potential energy “forward” ($P_{mm}$) and “backward” ($P_{3ob}$) distributions plotted as “offset” from the $\bar{\Delta U^{MM\to 3ob}}$ to simplify the x-axis. (**b**) Molecule **11**’s nonequilibrium work “forward” ($P_{mm}$) and “backward” ($P_{3ob}$) distributions plotted as “offset” from the $\bar{W^{MM\to 3ob}}$ to simplify the x-axis.

As seen with **2**, potential energy distributions coming from MM and 3ob equilibrium simulations are disparate, with merely 0.03% overlap (Figure 4a). Additionally, Π values (−3.26 and −3.38 for FEP (fw) and FEP (bw), respectively (see Table 2)) indicate that these potential energy distributions are not suited to providing converged FES between levels of theory. Poor convergence can also be seen in: high sample size hysteresis values (>1 kcal/mol), deviation between FEP (fw) and FEP (bw), and broadened $\Delta U^{MM\to 3ob}$ distributions themselves. By all metrics, FEP and BAR fail for molecule **11**, a small molecule in gas phase with chemical features seen in many experimental contexts. However, as again seen with molecule **2**, $W^{MM\to 3ob}$ distributions were far more suited for calculating converged $\Delta A_{gas}^{MM\to 3ob}$ (Figure 4b). Π values were much improved compared to their equilibrium counterparts, being 0.99 and 1.68 for JAR (fw) and JAR (bw), respectively; overlap between the two distributions is also dramatically improved at 44.12%; JAR (fw) and JAR (bw) are essentially absent of sample size hysteresis, and agree with each other within ≈0.5 kcal/mol.

**Figure 5:**
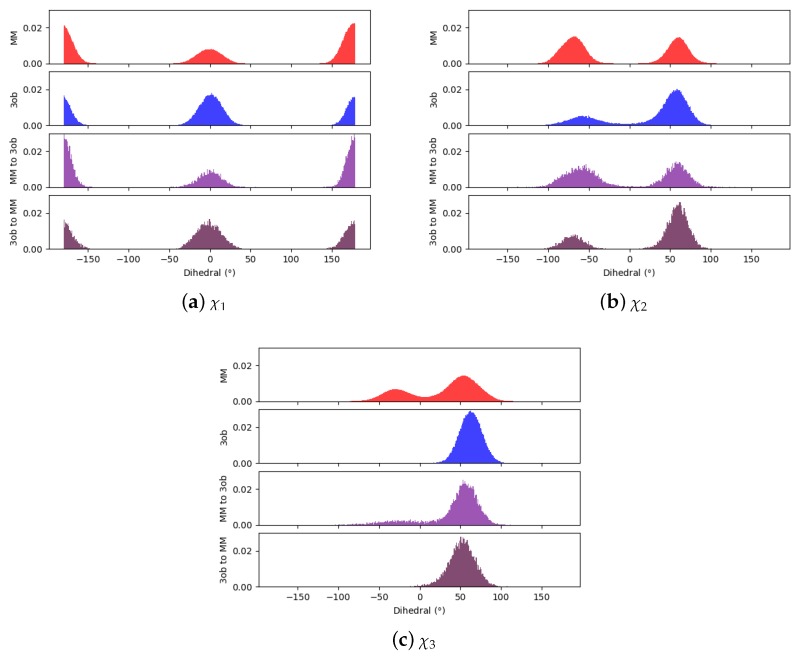
Dihedral populations for **11**’s dihedral degrees of freedom (see Figure 2 for dihedral labels).

Comparing dihedral distributions among MM, 3ob, and nonequilibrium switching simulations illustrates again MM and 3ob levels of theory agree on low energy dihedral conformations although they may not largely agree on relative energy differences between said dihedral angles (Figure 5). However, there appears to be enough dihedral overlap to allow for converged JAR and CRO, but likely distinctions between “stiff” degrees of freedom (i.e., bonds, angles) cause issues in FEP/BAR convergence, whereas bond/angle discrepancies are likely resolved during nonequilibrium switching simulations.

It again should be noted how poorly FEP performed compared to converged JAR/CRO: FEP (fw) predicts (excluding offset values in Table 1) $\Delta A_{gas}^{MM\to 3ob}$ to be −460.27 kcal/mol, whereas CRO predicts −409.50 kcal/mol (see Table 2 and Table 3), a difference of 49.23 kcal/mol. Again, considering the only sources of convergence difficulties here are the intramolecular degrees of freedom (i.e., bonds, angles, dihedrals, and intramolecular nonbonded interactions) one cannot expect error cancellation in a full indirect QM/MM FES.

### 3.2. The Bad

For several molecules, we could not obtain converged $\Delta A_{gas}^{MM\to 3ob}$ using JAR (fw) alone but rather needed long equilibrium 3ob simulations and nonequilibrium 3ob→MM simulations to obtain a converged value via CRO. These molecules include: **1**, **5**, **6**, **16**, **18**, **19**, **21**, and **22**. We focus our discussion here on molecules **5** and **6**, as representative examples.

#### 3.2.1. Molecule **5**

Molecule **5** (ZINC00087557, 3-phenylcyclopropane-1,2-dicrabohydrazide) contains functional groups useful in metal-organic chemistry [83], and thus has potential for future modeling focus not only in drug discovery projects but also in inorganic modeling. However, we have seen that it is quite difficult to obtain converged $\Delta A_{gas}^{MM\to 3ob}$ for this species even with nonequilibrium switching simulations.

**Figure 6:**
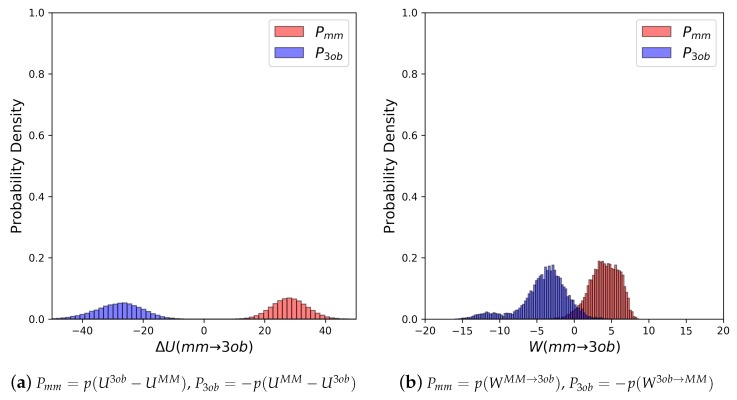
(**a**) Molecule **5**’s potential energy “forward” ($P_{mm}$) and “backward” ($P_{3ob}$) distributions plotted as “offset” from the $\bar{\Delta U^{MM\to 3ob}}$ to simplify the x-axis. (**b**) Molecule **5**’s nonequilibrium work “forward” ($P_{mm}$) and “backward” ($P_{3ob}$) distributions plotted as “offset” from the $\bar{W^{MM\to 3ob}}$ to simplify the x-axis.

Molecule **5**’s equilibrium $\Delta U^{MM\to 3ob}$ distributions are quite broad and exhibit little to no overlap (≈0.0%) (see Figure 6a). Convergence issues in FEP/BAR are further indicated by very low one-sided Π values, sample size hysteresis is seen in FEP (fw), FEP(bw) and BAR, and once again magnitude differences between FEP (fw) and FEP (bw) (see Table 2). Unlike with the “Good” molecules, although nonequilibrium simulations of **5** improved overlap and resulted in narrower distributions (Figure 6b), JAR (fw)/JAR (bw) metrics still indicate potential convergence failure and CRO results might require further validation. For example, for JAR (fw) Π = 0.08, and JAR (bw) Π = −0.66, both indicate unreliable one-sided distributions. Additionally, JAR (bw) exhibits sample size hysteresis (Hyst = 2.26 kcal/mol), and JAR (fw) and JAR (bw) disagree by 1.66 kcal/mol. CRO results, on the other hand, do barely pass convergence metrics: sample size hysteresis is low, and overlap is sufficient for a two-sided method at 8.76%. Thus, while CRO may be able to calculate a trustworthy result, it is far from the stellar nonequilibrium convergence results seen from “Good” molecules.

**Figure 7:**
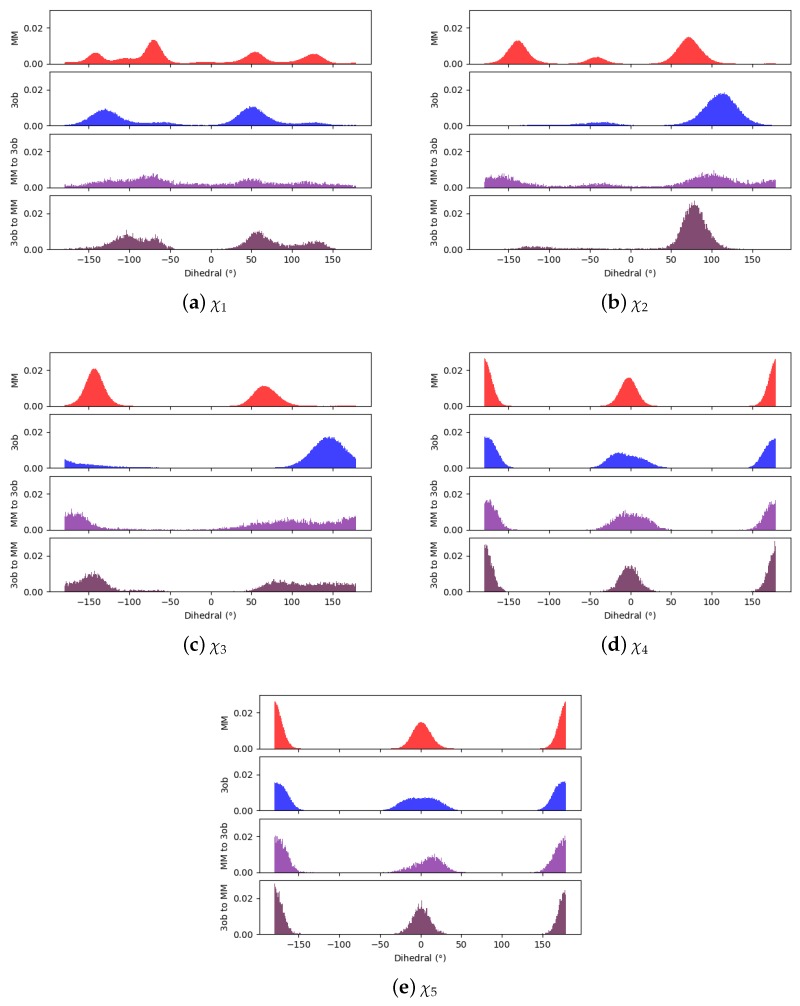
Dihedral populations for **5**’s dihedral degrees of freedom (see Figure 2 for dihedral labels).

Through examining the dihedral distributions of **5**, we see that $\chi_{2}$ and $\chi_{3}$ could be causing convergence issues in FEP, BAR, and JAR (Figure 7). For example, consider MM and 3ob dihedral populations in $\chi_{2}$ and $\chi_{3}$. These populations have distinct minimum angles at the two levels of theory, which doubtless are contributing to convergence failure when using FEP/BAR. However, when conducting MM→3ob switching simulations, the barriers to rotation from MM preferred dihedral conformations to 3ob preferred dihedral conformations may be too high to traverse during the shorter nonequilibrium switching simulations (i.e., 1 ps). Although the switching simulation dihedral populations are more similar to their respective target level of theories, there are still discrepancies. For example, considering **5**’s $\chi_{3}$, we see MM predicts two low energy angles at 60${}^{\circ }$ and −140${}^{\circ }$ while 3ob predicts one low energy dihedral value at 140${}^{\circ }$. After MM→3ob switching simulations, the two peaks predicted by MM relax to one larger peak vaguely encompassing the low energy region in 3ob simulations, although this low energy dihedral peak does not have the same population density as seen from 3ob equilibrium simulations. It is likely that longer switching simulations may be needed to allow these dihedral degrees of freedom to completely relax. However, by pooling both $W^{MM\to 3ob}$ and $W^{3ob\to MM}$ values, we were able to obtain marginally converged CRO results.

Finally, CRO predicts $\Delta A_{gas}^{MM\to 3ob}$ to be −537.50 kcal/mol, while FEP(fw) predicts −587.63 kcal/mol and JAR (fw) −539.16 kcal/mol. The FEP (fw) result is again ≈50 kcal/mol from the CRO value; an error which almost certainly will not be resolved via error cancellation in the indirect scheme. However, the JAR (fw) result is only ≈1.66 kcal/mol from the CRO value. Thus, the JAR (fw) result may have given essentially the “correct” result in an indirect cycle (“correct” when compared to our calculated CRO value), yet this result may have been spurious, and thus it is always wise to evaluate convergence metrics before trusting nonequilibrium switching simulations to resolve most configurational disparities in a distribution.

#### 3.2.2. Molecule **6**

Molecule **6** (ZINC00095858, ethyl *N*-[(2-chlorophenyl)sulfonyl]carbamate) is a flexible small molecule with thousands of similar structures available in the PubChem Database, thus ensuring accurate FES modeling of **6** could be beneficial for modeling many other small molecules.

**Figure 8:**
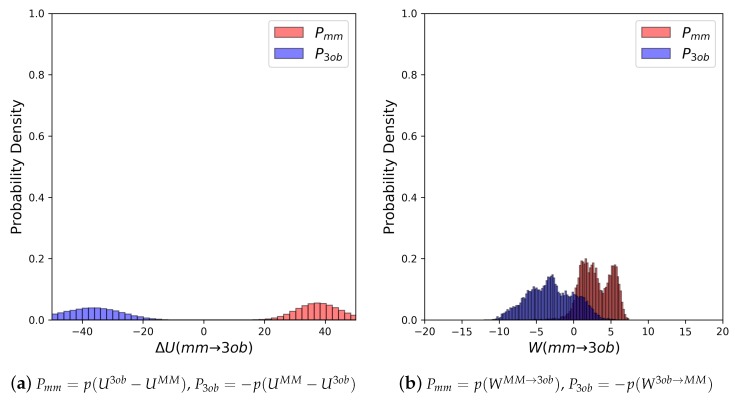
(**a**) Molecule **6**’s Potential energy “forward” ($P_{mm}$) and “backward” ($P_{3ob}$) distributions plotted as “offset” from the $\bar{\Delta U^{MM\to 3ob}}$ to simplify the x-axis. (**b**) Molecule **6**’s nonequilibrium work “forward” ($P_{mm}$) and “backward” ($P_{3ob}$) distributions plotted as “offset” from the $\bar{W^{MM\to 3ob}}$ to simplify the x-axis.

As seen with **5**, FEP and BAR results for **6** are unreliable: $\Delta U^{MM\to 3ob}$ distributions are broad and non-overlapping (Figure 8a), Π values are poor, sample size hysteresis for FEP (fw) and FEP (bw) is high, and FEP (fw) and FEP (bw) differ by ≈21 kcal/mol (see Table 2). Unfortunately, as with with FEP, JAR (fw) and JAR (bw) results are also not immediately trustworthy, with one-sided Π values of −0.65 and −0.47 for JAR (fw) and JAR (bw), respectively, and discrepancy between JAR (fw) and JAR (bw) of ≈5 kcal/mol (see Table 3). However, by utilizing data from both MM→3ob and 3ob→MM switching simulations, i.e., CRO, we were able to calculate a marginally converged $\Delta A_{gas}^{MM\to 3ob}$. Overlap between nonequilibrium work distributions (23.95%) is much improved compared to $\Delta U^{MM\to 3ob}$ distributions (0.00%).

**Figure 9:**
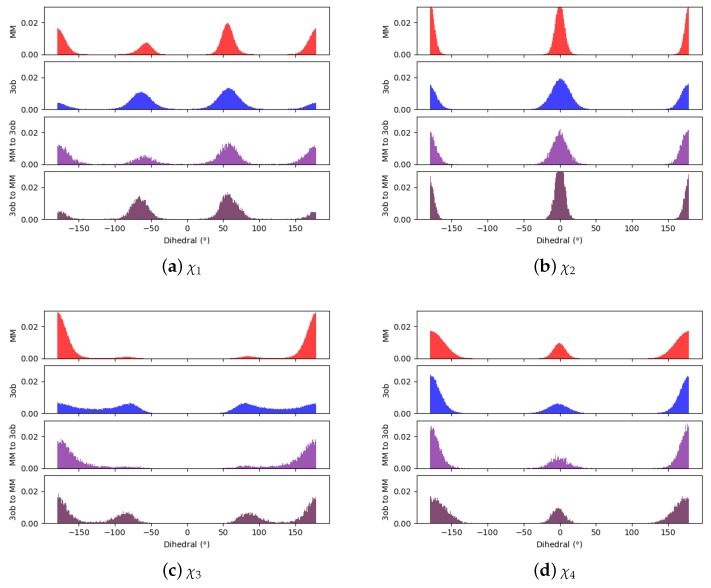
Dihedral populations for **6**’s dihedral degrees of freedom (see Figure 2 for dihedral labels).

Again, qualitatively comparing dihedral distributions illuminates possible causes for JAR convergence failures (Figure 9). Although most of the dihedral populations appear largely similar for **6**, $\chi_{3}$ may be distinct enough to cause convergence errors, certainly with FEP/BAR as trans-$\chi_{3}$ is vastly overrepresented from MM simulations. This may also be the case in JAR (fw) and JAR (bw) results, as MM→3ob switching simulations are unable to replicate the near degeneracy of the trans- and gauche-$\chi_{3}$ conformations as seen in 3ob simulations. However, pooling together all switching simulations may provide enough gauche-$\chi_{3}$ conformations to achieve convergence.

Once again, we should point out that FEP (fw) predicts $\Delta A_{gas}^{MM\to 3ob}$ to be −109.58 kcal/mol, while CRO predicts $\Delta A_{gas}^{MM\to 3ob}$ to be −140.17 kcal/mol, an error of ≈30 kcal/mol, and an error which would not necessarily be resolved in error cancellation within the indirect cycle. Thus, using convergence metrics, such as Π, is important for evaluating the reliability of a dataset.

### 3.3. The Ugly

Finally, there were three molecules for which we observed severe convergence issues even when using CRO: molecules **8**, **9**, and **20**. We focus our discussion here on molecules **8** and **9**.

#### 3.3.1. Molecule **8**

Molecule **8** (ZINC ID 00107778, 4,6-dichloro-2*H*-chromene-3-carbaldehyde oxime) is another oxime species similar to molecule **2**. As mentioned above, oxime species have been used recently as promising anti-cancer agents, and thus the computational community should ensure our methods can properly model such compounds [82,84,85,86].

**Figure 10:**
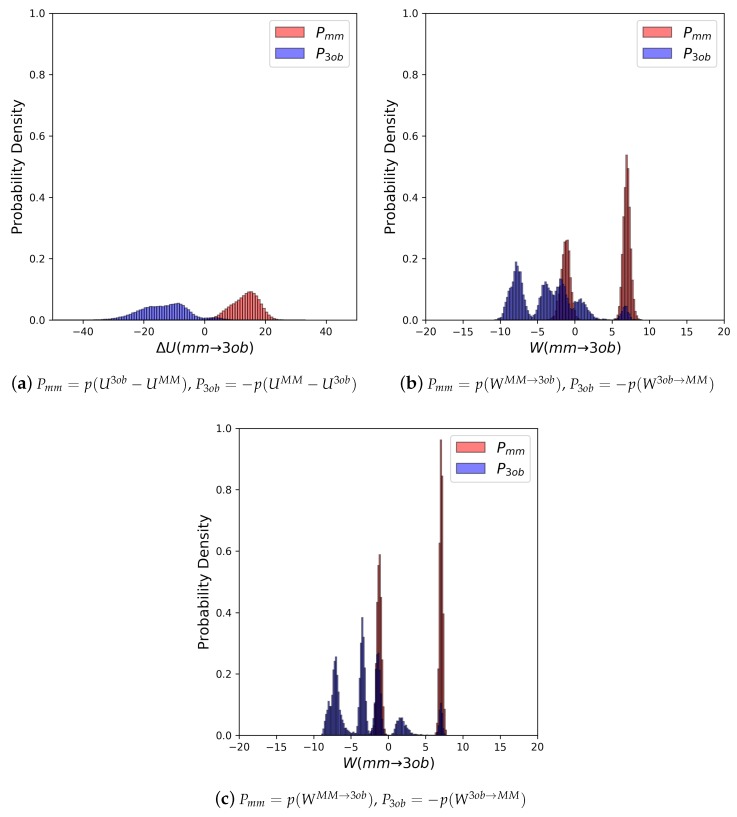
(**a**) Molecule **8**’s potential energy “forward” ($P_{mm}$) and “backward” ($P_{3ob}$) distributions plotted as “offset” from the $\bar{\Delta U^{MM\to 3ob}}$ to simplify the x-axis. (**b**) Molecule **8**’s nonequilibrium work “forward” ($P_{mm}$) and “backward” ($P_{3ob}$) distributions from 1 ps switching simulations plotted as “offset” from the $\bar{W^{MM\to 3ob}}$ to simplify the x-axis. (**c**) Molecule **8**’s nonequilibrium work “forward” ($P_{mm}$) and “backward” ($P_{3ob}$) distributions from 5 ps switching simulations plotted as “offset” from the $\bar{W^{MM\to 3ob}}$ to simplify the x-axis.

As in earlier cases, FEP/BAR results again are unconvincing. However, interestingly, the metrics do not indicate there should be much more of a convergence problem than as seen for the “Bad” molecules. Π values illustrate the $\Delta U^{MM\to 3ob}$ distributions are unreliable as expected, and yet FEP (fw) and FEP (bw) differ by only ≈6 kcal/mol, and $\Delta U^{MM\to 3ob}$ distributions do exhibit barely enough overlap for a converged result at 3.74% (Figure 10). Surprisingly, this potential energy overlap percentage indicates equilibrium results for **8** should result in marginally converged $\Delta A_{gas}^{MM\to 3ob}$, and yet other analyses of ΔU distributions indicate these are unreliable datasets. Even though $W^{MM\to 3ob}$ distributions do overlap considerably better than $\Delta U^{MM\to 3ob}$, at 24.92%, Π evaluations of $W^{MM\to 3ob}$ (fw and bw) distributions indicate only marginally improved reliability, and not enough to be sufficiently confident in JAR or even CRO results. Additionally, the *W* distributions in Figure 10b seem to be oddly polymodal. Considering the difficulties in convergence observed, we conducted longer switching simulations (5 ps) in the hopes of improving convergence by allowing longer relaxation times. Data from 5 ps switching simulations are given in Table 3 in row “**8 (5 ps)**”, and distributions are shown in Figure 10c. Unfortunately, even conducting 5 ps switching simulations did not allow for significantly improved $W^{MM\to 3ob}$ distributions.

**Figure 11:**
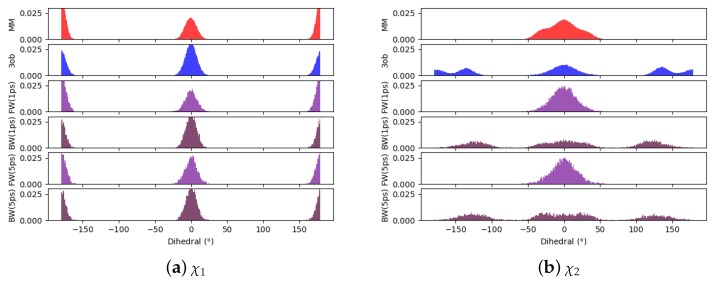
Dihedral populations for **8**’s dihedral degrees of freedom (see Figure 2 for dihedral labels).

Examining **8**’s dihedral populations may provide insight into this molecule’s convergence issues: although $\chi_{1}$ is fairly consistent between MM and 3ob distributions, $\chi_{2}$ is quite distinct between MM and 3ob (see Figure 11). MM and 3ob simulations agree there is a low energy cis-$\chi_{2}$ conformation, however 3ob simulations also predict the gauche- and trans-$\chi_{2}$ conformation is populated, while MM simulations do not visit this region. It should also be noted, as described in the Methods, equilibrium simulations were launched by initiating randomized dihedrals to ensure thorough dihedral sampling. Even after randomizing $\chi_{2}$, MM simulations did not visit trans regions that were shown to be energetically stable in 3ob simulations. Furthermore, even after MM→3ob switching simulations of 1 ps and 5 ps, MM configurations are not able to relax into the trans- and gauche-$\chi_{2}$ conformations predicted by 3ob. Thus, the barrier to rotation around $\chi_{2}$ must be too high to overcome, even during longer/slower switching protocols. This is a case where intramolecular force matching may improve low-level classical parameters and thus overlap to the higher level of theory.

Finally, FEP (fw) predicts $\Delta A_{gas}^{MM\to 3ob}$ = −944.00 kcal/mol, JAR (fw, 1 ps) gives −991.95 kcal/mol, JAR (fw, 5 ps) gives −995.88 kcal/mol, and CRO(5 ps) gives −995.87 kcal/mol. Thus, although conducting lengthened switching simulations in this case does appear to improve convergence (Π values improve marginally from 1 ps to 5 ps switching protocols, i.e., JAR (fw, 5 ps) and CRO(5 ps) are in closer agreement than their 1 ps counterparts), it is clear from visualizing the $W^{MM\to 3ob}$ distributions that these values are not converged. Additionally, FEP (fw) is ≈50 kcal/mol from the CRO(5 ps) result, although even CRO(5 ps) result cannot be entirely trusted. Thus, molecule **8** is truly one of the toughest convergence cases in our HiPen dataset.

#### 3.3.2. Molecule **9**

Molecule **9** (ZINC ID 00123162, 1-phenyl-1,2,3-butanetrione 2-[*N*-(4-chlorophenyl)hydrazone]) contains chemical features seen in thousands of molecules available in the PubChem database, and therefore ensuring appropriate FES modeling with **9** could ensure appropriate FES modeling of many other compounds in the near future.

**Figure 12:**
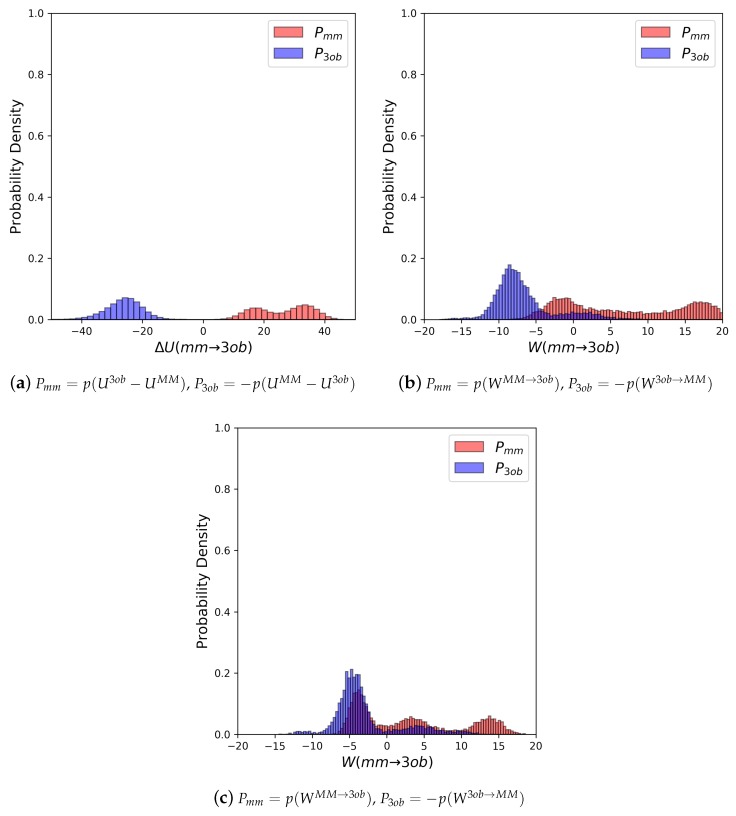
(**a**) Molecule **9**’s potential energy “forward” ($P_{mm}$) and “backward” ($P_{3ob}$) distributions plotted as “offset” from the $\bar{\Delta U^{MM\to 3ob}}$ to simplify the x-axis. (**b**) Molecule **9**’s nonequilibrium work “forward” ($P_{mm}$) and “backward” ($P_{3ob}$) distributions from 1 ps switching simulations plotted as “offset” from the $\bar{W^{MM\to 3ob}}$ to simplify the x-axis. (**c**) Molecule **9**’s nonequilibrium work “forward” ($P_{mm}$) and “backward” ($P_{3ob}$) distributions from 5 ps switching simulations plotted as “offset” from the $\bar{W^{MM\to 3ob}}$ to simplify the x-axis.

Equilibrium FES methods were, once again, unable to calculate reliable $\Delta A_{gas}^{MM\to 3ob}$ for **9**: FEP (fw), FEP (bw), and BAR exhibited sample size hysteresis; FEP (fw) and FEP (bw) did not agree in magnitude; Π values did not indicate well-behaved ΔU distributions at −4.27 and −4.77 for FEP (fw) and FEP (bw), respectively; and finally forward and backward ΔU distributions exhibited only 0.02% overlap (see Figure 12a). Additionally, 1 ps nonequilibrium switching protocol did not improve according to convergence criteria as would be expected: JAR (fw, 1 ps), JAR (bw, 1 ps), and CRO (1 ps) exhibit considerable sample size hysteresis; JAR (fw, 1 ps) and JAR (bw, 1 ps) differ in magnitude by ≈11 kcal/mol; and Π values are −4.64 and −2.48, for JAR (fw, 1 ps) and JAR (bw, 1 ps), respectively, with 22.83% overlap (see Figure 12b). As such, we once again conducted longer nonequilibrium switching simulations (5 ps). Much like with **8**, such longer nonequilibrium switching simulations only marginally improved results compared to 1 ps switching simulations. JAR (fw, 5 ps) and JAR (bw, 5 ps) still do not agree in magnitude, although JAR (fw, 5 ps) agrees with CRO (5 ps), and JAR (bw, 5 ps) exhibits ≈10 kcal/mol in sample size hysteresis. Calculated Π values (−1.24 and −2.95, for JAR (fw, 5 ps) and JAR (bw, 5 ps), respectively) indicate *W* distributions after 5 ps are still not well behaved.

**Figure 13:**
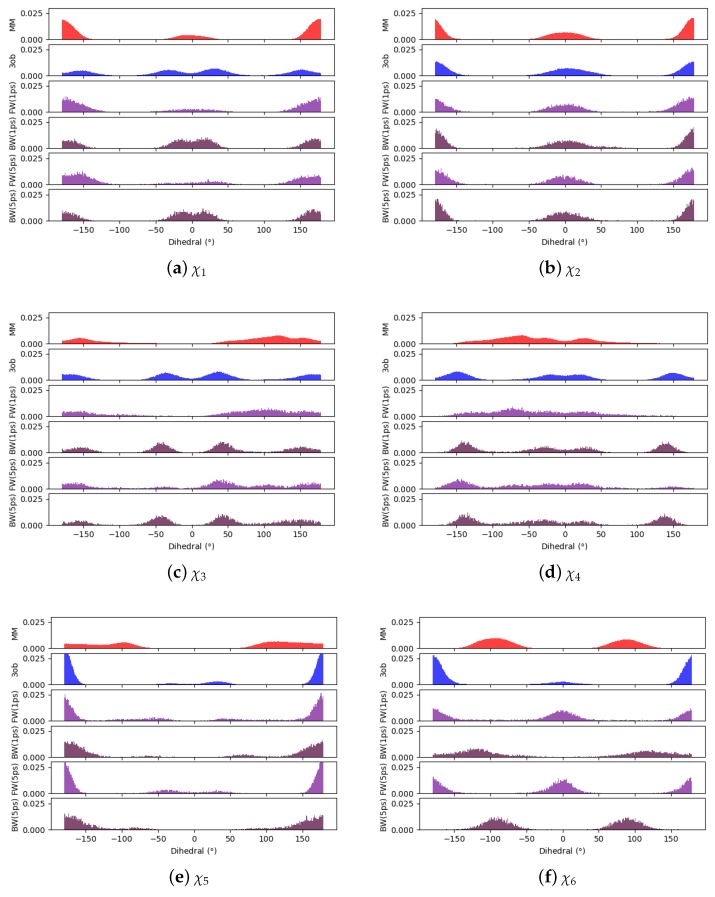
Dihedral populations for **9**’s dihedral degrees of freedom (see Figure 2 for dihedral labels).

Given the difficulty in arriving at a converged $\Delta A_{gas}^{MM\to 3ob}$ for **9**, we again hoped to pin-point convergence issues in dihedral degrees of freedom, Figure 13. As can be seen in Figure 13a,b, $\chi_{1}$ and $\chi_{2}$ distributions between equilibrium MM and 3ob simulations are fairly similar, low energy dihedral conformations are consistent between levels of theory and relative populations between such angles are also consistent. However, for $\chi_{3}$, $\chi_{4}$, $\chi_{5}$, and $\chi_{6}$, there are large discrepancies between MM and 3ob regarding the low energy dihedral values and their relative populations, such discrepancy is especially clear in $\chi_{6}$ (Figure 13g). Furthermore, such discrepancies are not completely resolved within 1, or even 5 ps nonequilibrium switching simulations, as is the case for $\chi_{3}$ and $\chi_{4}$. Thus, these dihedrals likely represent the roadblock to converged $\Delta A^{low\to high}$ for **9** and further likely require intramolecular force matching to be resolved.

## 4. Materials and Methods

All molecular modeling described herein was conducted using CHARMM (Chemistry at Harvard Molecular Modeling) software (version C43a2) [87].

### 4.1. Equilibrium Simulations

The complete Maybridge Hitfinder set, a curated online database of “drug-like” (according to Lipinski rules) molecules (https://www.maybridge.com) was scanned through ParamChem, an online tool for generating parameter and topology files used in CHARMM (https://cgenff.umaryland.edu). Standard ParamChem output includes listing “parameter and charge penalties”. These penalties represent how trustworthy the output parameters and topologies are, thus higher penalties may indicate less trustworthy parameters and potential modeling inaccuracies. From the Maybridge Hitfinder set, 22 molecules were chosen which returned high parameter or charge penalties from ParamChem and these 22 molecules constituted our “HiPen” dataset.

Initial 3D coordinates of each HiPen molecule were collected from the ZINC${}_{12}$ Structural Database (http://zinc.docking.org [88]); see Table 1 for the ZINC IDs. As mentioned, CGenFF (CHARMM Generalized Force Field [56]) parameter and topology files for each molecule were obtained through the ParamChem web interface (https://cgenff.umaryland.edu [54,55]). The starting coordinates were optimized by 1000 steps of Steepest Descent minimization, followed by 1000 steps of Adopted Basis Newton–Raphson minimization. To calculate $\Delta A_{gas}^{MM\to 3ob}$ according to the various methods compared in this work (FEP, BAR, JAR, and CRO), Langevin Dynamics (LD) simulations were performed at the two levels of theory, MM and SCC-DFTB/3*ob*. In all cases, a friction coefficient of 5 ps^−1^ was applied to all atoms, and random velocities were added at each step corresponding to a temperature bath of 300 K.

***MM simulations:*** For each molecule, *ten* LD simulations were carried out, which were started from different initial random velocities. Additionally, to enhance sampling, we employed different starting coordinates if/when the molecule contained rotatable bonds (cf. Figure 2). First, all rotatable bonds were randomized. Next, 1000 steps of Adopted Basis Newton–Raphson minimization were carried out while restraining the dihedral angles harmonically (k=100 kcal/mol/A^2^) to their randomized value(s). Finally, restraints were removed and 10 ps of LD were carried out as equilibration. As molecules were simulated in the gas phase, all nonbonded interactions were calculated explicitly during the simulation; neither switching nor shifting functions were applied. Following these preparation steps, 10 million steps of LD were carried out with a timestep of 1 fs; this corresponds to 10 ns per run, and a cumulative simulation length of 100 ns for the *ten* runs per molecule. Restart files (containing both coordinates and velocities) were saved every 100 steps, resulting in 1 million coordinate and velocity sets per molecule. For use in FEP and BAR, the energies for each coordinate set were computed at both the MM and SCC-DFTB/3*ob* levels of theory. We further computed the instantaneous dihedral angles of all rotatable bonds considered.

***SCC-DFTB3/3ob simulations:*** All simulations employing the SCC-DFTB/3*ob* potential energy function, were conducted according to a protocol closely mirroring what was just described for the MM simulations. However, in the case of SCC-DFTB/3*ob* simulations, the simulation length per run (again, 10 runs per molecule) was only 1 ns (a timestep of 1 fs was used); the cumulative simulation length, therefore, was 10 ns. Coordinate and velocity information was saved every 100 steps resulting in a total of 100,000 restart files. As in the MM case, for each of the coordinate sets we computed the energy at the force field and SCC-DFT3/3*ob* levels of theory, as well as the instantaneous values of the dihedrals of the rotatable bonds.

### 4.2. Nonequilibrium “Switching” Simulations

To compute $\Delta A_{gas}^{MM\to 3ob}$ using JAR (Equation (5)) or CRO (Equation (6)), one must repeatedly compute the nonequilibrium work for switching from the MM Hamiltonian to the SCC-DFTB/3*ob* Hamiltonian and/or vice versa. In CHARMM, this can be accomplished using the program’s MSCALE [89] and PERT [87] functionalities. The multi-scale (MSCALE) modeling module of CHARMM allows the user to treat a system, in part or whole, according to two (or more) different energy functions. In the present case, MSCALE is employed to mix the MM and SCC-DFTB/3*ob* energy functions as needed. In combination with the PERT free energy facility of CHARMM in slow-growth mode, the degree of mixing can be changed continuously from 100% MM to 100% SCC-DFTB/3*ob*. Since during switching over any finite time window the system is not at equilibrium, the “energy differences” obtained in slow-growth calculations really are nonequilibrium work values. This is even more the case when switches are carried out very quickly (within a few ps or less) to avoid excessive computational cost, as is the case when switching to (S)QM/MM Hamiltonians. For full details, we refer the reader to our earlier work [33,34]; additionally, a recent general review about nonequilibrium work methods can be found in Reference [90]. Switching simulations in both forward (MM → SCC-DFTB/3*ob*) and backward (SCC-DFTB/3*ob* → MM) direction were started from the restart files saved during the respective equilibrium simulations (see above). The timestep during all switching simulations was 1 fs.

***MM to 3ob Switching simulations:***MM→3ob switching simulations were launched from every 10,000th MM simulation step, i.e., from snapshots saved at 10 ps intervals during the MM equilibrium simulations. Per molecule, this resulted in a total of 10,000 MM→3ob nonequilibrium switches. Unless otherwise noted, all switching simulations were conducted for 1 ps (1000 steps). $W^{MM\to 3ob}$ was recorded per switch and post-processed using JAR and CRO. For each of the final coordinates, we also computed the dihedral angles of the rotatable bonds.

***3ob to MM Switching simulations:***3ob→MM switching simulations were launched from every 1000th 3ob simulation step, or every 1 ps. Per molecule, this resulted in a total of 10,000 3ob→MM nonequilibrium simulations. Unless otherwise noted, all switching simulations were conducted for 1 ps (1000 steps). $W^{3ob\to MM}$ was recorded per switch and post-processed according to JAR and CRO. As was done at the end of the MM→3ob switching simulations, dihedral angle values were recorded.

## 5. Conclusions

We present here a new dataset to be used for future method development in the QM/MM FES community. In particular, we have calculated $\Delta A_{gas}^{MM\to 3ob}$ for 22 drug-like small molecules obtained from the Maybridge Hitfinder set. In compiling our dataset, we observed three categories of molecules emerge: “good”, molecules for which JAR (fw) could obtain a reliably converged $\Delta A_{gas}^{MM\to 3ob}$; “bad”, molecules for which JAR (fw) results proved unreliable but CRO results were reliably converged; and “ugly” molecules for which even CRO could not produce reliably converged results. Although we have yet to derive any strict/concrete patterns quantitatively relating our convergence criteria to how “wrong” a calculated $\Delta A^{low\to high}$ may be (i.e., we cannot yet tell from a one-sided Π value calculated from $\Delta U^{MM\to 3ob}$ distribution how wrong the resultant FEP (fw) will be), we have illustrated that several convergence metrics should always be evaluated and compared before trusting a ΔA, especially those calculated from equilibrium FES. We have also seen, once again, how discrepancies in “stiff” and “soft” degrees of freedom between levels of theory can result in drastic convergence errors which may not always be ameliorated via nonequilibrium work (even extended) switching simulations, as was seen in the case of “ugly” molecules **8** and **9**. We intend to use these data in the near future for further method development as well as evaluating the same criterion/metrics in solvent phase free energy simulations. Furthermore, we hope this dataset will prove as useful to FES practitioners in providing standardized results as the BEGDB and MNDB2.0 datasets are to the quantum mechanical calculation community.

## Figures and Tables

**Table 1:** ZINC Database ID’s for each HiPen molecule; the total number of atoms ($N_{tot}$) and total number of heavy atoms ($N_{heavy}$) per molecule; ParamChem reported CGenFF penalties; calculated ΔA “offset” for each molecule in the dataset should be added or subtracted (to positive and negative ΔA’s respectively) to every ΔA listed in Table 2 and Table 3 to give the total calculated ΔA.

	ZINC ID	$N_{\mathrm{tot}}/N_{\mathrm{Heavy}}$	CGenFF Penalties		Offset (kcal/mol)
			Param	Charge	
1	00061095	36/21	432.10	200.99	29,000
2	00077329	16/10	378.50	347.24	15,000
3	00079729	18/13	683.00	207.72	17,000
4	00086442	21/12	312.50	283.62	19,000
5	00087557	31/17	378.50	347.31	25,000
6	00095858	25/16	567.90	361.40	25,000
7	00107550	21/11	378.50	347.29	16,000
8	00107778	22/15	378.50	347.29	21,000
9	00123162	34/21	385.50	217.28	29,000
10	00133435	34/22	470.50	27.14	28,000
11	00138607	36/20	336.00	261.56	29,000
12	00140610	20/12	449.00	214.90	17,000
13	00164361	23/14	424.00	194.49	20,000
14	00167648	44/26	436.50	226.60	35,000
15	00169358	26/16	540.40	142.16	22,000
16	01755198	28/12	329.00	21.11	19,000
17	01867000	32/18	470.50	5.82	22,000
18	03127671	41/24	329.00	25.20	34,000
19	04344392	52/29	329.00	24.78	40,000
20	04363792	28/21	698.00	185.49	28,000
21	06568023	30/18	329.00	21.60	25,000
22	33381936	33/21	545.50	395.62	30,000

**Table 2:** Equilibrium results. $\Delta A^{MM\to 3ob}$ calculated with FEP (fw), FEP (bw), and BAR, as well as calculated convergence metrics. For each ΔA, we divide the total dataset into 10 blocks, calculate $\Delta A_{i}$ from each of these *i* blocks, and compare the average of those $\Delta A_{i}$ to ΔA calculated from the total dataset (giving Hyst), and we also report the standard deviation of these $\Delta A_{i}$ ($\sigma_{\Delta A}$). To determine the reliability of ΔU distributions for calculating ΔA we report: $\bar{\Delta U}$, the standard deviation of ΔU’s ($\sigma_{\Delta U}$) as narrower distributions are likely to provide converged results, and finally we report one-sided Π which, when >0.5, likely indicates the ΔU distribution is resultant from sufficient and unbiased sampling. Finally, we report percentage overlap in ΔU between forward and backward distributions.

	FEP (fw)						FEP (bw)						BAR			Overlap (%)
	ΔA	Hyst	$\sigma_{\Delta A}$	$\bar{\Delta U}$	$\sigma_{\Delta U}$	Π	ΔA	Hyst	$\sigma_{\Delta A}$	$\bar{\Delta U}$	$\sigma_{\Delta U}$	Π	ΔA	Hyst	$\sigma_{\Delta A}$	ΔU
1	−301.11	4.08	5.29	−282.47	7.89	−3.14	305.82	1.87	2.02	322.87	7.30	−3.29	−303.34	−0.15	3.15	0.04
2	−255.52	0.19	0.51	−245.10	4.29	−1.15	258.88	0.29	0.60	268.72	5.28	−1.47	−256.87	−0.05	0.11	0.28
3	−412.88	0.35	0.65	−402.47	3.49	−1.17	416.38	0.54	0.78	428.26	4.86	−2.06	−414.19	−0.01	0.01	0.12
4	−254.51	0.43	0.69	−239.09	5.66	−2.43	259.24	0.51	0.84	269.67	4.34	−1.64	−256.34	0.00	0.03	0.06
5	−589.94	2.30	2.38	−570.06	5.78	−3.41	604.61	2.25	2.49	626.49	7.84	−4.29	−596.97	0.48	0.25	0.00
6	−109.58	2.58	2.59	−86.31	7.14	−4.07	130.57	4.83	4.41	162.27	10.42	−6.04	−118.33	0.56	2.35	0.00
7	−992.13	0.33	0.67	−982.02	3.94	−1.06	994.96	4.83	12.05	1011.88	19.05	−3.26	−993.15	0.21	0.49	0.28
8	−994.00	4.16	4.03	−982.45	4.39	−1.46	988.29	9.09	5.74	1009.22	7.77	−4.10	−992.02	1.03	14.08	3.72
9	−447.42	2.99	3.12	−423.12	9.04	−4.27	451.15	8.94	4.93	475.54	6.40	−4.77	−449.44	1.02	5.61	0.02
10	−336.30	0.84	0.99	−320.41	5.46	−2.54	341.91	0.31	0.72	352.24	4.10	−1.61	−337.98	0.11	0.07	0.04
11	−460.25	1.37	1.30	−441.09	7.09	−3.26	464.90	1.43	1.15	482.37	8.89	−3.38	−461.88	0.11	0.07	0.02
12	−70.74	2.17	1.31	−54.33	4.82	−2.66	84.97	0.85	1.21	115.59	11.07	−5.86	−77.20	0.25	0.02	0.00
13	−556.49	2.79	1.67	−547.25	5.39	−3.27	571.83	1.37	1.32	587.80	5.62	−3.28	−567.59	0.18	0.15	0.01
14	−80.28	0.79	0.97	−65.97	4.62	−2.17	85.55	0.78	0.77	100.32	6.15	−2.89	−82.62	0.12	0.10	0.03
15	−406.76	0.18	0.46	−398.31	3.39	−0.56	408.29	0.32	0.54	419.87	5.52	−2.04	−407.59	0.02	0.00	0.51
16	−633.17	1.32	1.57	−621.65	4.28	−1.45	638.22	2.26	2.73	664.09	8.08	−5.12	−636.14	0.72	0.68	0.05
17	−673.11	0.21	0.55	−664.21	3.29	−0.70	672.67	−0.13	0.71	682.01	3.11	−1.71	−673.41	−0.03	0.01	0.69
18	−518.20	1.82	1.80	−501.32	5.34	−2.76	525.15	4.99	4.91	551.31	9.84	−5.09	−520.79	0.90	3.94	0.10
19	−879.27	3.54	2.13	−857.72	6.10	−3.74	892.96	1.86	2.43	918.55	9.50	−4.99	−885.39	0.51	0.21	0.00
20	−691.39	3.08	4.83	−676.22	6.43	−2.37	713.26	0.83	1.34	753.13	14.99	−7.29	−702.33	0.83	1.13	0.00
21	−70.62	2.52	1.58	−59.20	3.66	−1.43	69.86	1.10	1.30	87.37	8.66	−3.39	−69.33	0.25	0.84	0.37
22	−177.51	0.73	0.97	−165.15	4.25	−1.68	181.81	0.82	1.04	213.38	37.01	−6.02	−179.19	0.14	0.22	0.07

**Table 3:** Nonequilibrium results. $\Delta A^{MM\to 3ob}$ calculated with JAR (fw), JAR (bw), and CRO, as well as calculated convergence metrics. For each ΔA, we divide the total dataset into 10 blocks, calculate $\Delta A_{i}$ from each of these *i* blocks, and compare the average of those $\Delta A_{i}$ to ΔA calculated from the total dataset (giving Hyst), and we also report the standard deviation of these $\Delta A_{i}$ ($\sigma_{\Delta A}$). To determine the reliability of *W* distributions for calculating ΔA, we report: $\bar{W}$, the standard deviation of *W*’s ($\sigma_{W}$) as narrower distributions are likely to provide converged results, and finally we report one-sided Π which, when >0.5, likely indicates the *W* distribution is resultant from sufficient and unbiased sampling. Finally, we report percentage overlap in *W* between forward and backward distributions.

	JAR (fw)						JAR (bw)						CRO			Overlap (%)
	ΔA	Hyst	$\sigma_{\Delta A}$	$\bar{W}$	$\sigma_{W}$	Π	ΔA	Hyst	$\sigma_{\Delta A}$	$\bar{W}$	$\sigma_{W}$	Π	ΔA	Hyst	$\sigma_{\Delta A}$	*W*
1	−305.97	2.92	3.87	−299.85	5.07	−0.80	300.95	0.47	0.39	302.15	1.97	1.60	−301.61	0.53	4.44	32.71
2	−272.53	0.21	0.48	−271.48	0.60	1.86	271.88	0.13	0.48	272.59	1.59	2.19	−271.84	0.09	0.08	53.89
3	−408.69	0.00	0.03	−408.18	0.90	2.40	408.63	0.00	0.05	409.03	0.63	2.57	−408.66	0.00	0.00	56.68
4	−271.25	0.00	0.03	−271.10	0.41	3.02	271.15	0.00	0.02	271.31	0.49	3.00	−271.20	0.00	0.00	79.52
5	−539.16	0.58	0.98	−535.17	1.98	0.08	537.50	2.26	2.71	543.25	3.18	−0.66	−538.78	0.36	0.37	8.76
6	−143.51	3.69	2.10	−137.78	2.08	−0.65	138.57	1.46	2.12	143.83	3.12	−0.47	−140.17	0.41	1.70	23.95
7	−999.40	0.42	0.69	−998.41	0.69	1.91	998.80	1.37	2.82	1000.98	3.29	1.03	−999.02	0.42	0.28	44.88
8	−995.50	3.54	3.79	−990.77	4.06	−0.25	989.35	6.99	4.61	998.13	4.05	−1.69	−995.41	0.90	9.68	24.92
8 (5 ps)	−995.88	2.90	3.74	−991.48	4.16	−0.18	989.36	7.05	3.29	997.91	3.78	−1.71	−995.87	0.68	1.60	27.70
9	−426.22	1.72	1.95	−414.69	8.21	−2.48	415.08	7.74	5.19	428.09	4.12	−2.87	−423.89	0.91	8.28	22.83
9 (5 ps)	−426.53	−0.28	1.04	−419.30	7.09	−1.24	412.65	9.23	5.75	425.23	4.45	−2.95	−425.45	1.22	6.82	60.27
10	−285.45	0.02	0.15	−284.78	0.82	2.25	285.36	0.00	0.03	286.22	1.18	2.03	−285.41	0.01	0.00	46.14
11	−510.05	0.02	0.17	−507.81	2.93	0.99	509.50	0.23	0.41	510.72	0.96	1.68	−509.92	0.03	0.01	44.12
12	−81.64	0.00	0.03	−81.37	0.55	2.78	81.48	0.00	0.03	81.77	0.63	2.73	−81.56	0.00	0.00	72.35
13	−558.93	0.00	0.02	−558.82	0.36	3.13	558.80	0.00	0.01	558.91	0.36	3.12	−558.86	0.00	0.00	84.07
14	−61.10	0.00	0.05	−60.35	0.91	2.08	60.95	−0.01	0.09	61.75	0.99	1.97	−61.03	0.02	0.00	45.35
15	−408.64	0.00	0.02	−408.50	0.39	2.63	408.56	0.00	0.00	408.70	0.42	3.03	−408.59	0.01	0.00	76.59
16	−604.94	1.73	2.59	−600.37	2.46	−0.55	599.77	2.54	0.82	607.38	4.70	−1.37	−602.79	0.52	2.78	33.53
17	−672.92	0.00	0.02	−672.79	0.38	3.08	672.88	0.00	0.01	673.01	0.41	3.07	−672.90	0.00	0.00	76.92
18	−533.65	2.30	2.30	−527.81	2.42	−0.69	529.28	2.69	3.09	536.11	5.43	−1.05	−530.42	0.61	3.72	26.08
19	−912.05	4.65	3.21	−904.31	3.36	−1.53	906.22	0.00	0.81	909.91	3.03	0.05	−907.05	0.45	1.40	25.82
20	−704.48	2.63	4.45	−699.99	4.35	−0.18	697.46	5.67	3.51	706.31	2.49	−1.72	−704.26	0.77	7.32	13.21
21	−55.94	1.36	1.40	−53.45	1.12	0.82	53.51	−0.02	0.04	54.19	1.09	2.05	−53.70	0.09	0.53	60.60
22	−172.40	6.19	3.39	−162.42	1.51	−2.45	165.24	0.37	0.78	171.36	7.48	−0.80	−165.13	0.11	0.20	9.71

## Supplementary Materials

The following are available online. This Supporting Information contains dihedral distribution plots and potential energy overlap plots for all 22 molecules in the HiPen set. Additionally, all topology, minimized coordinates, parameters, and necessary input files can be found at https://zenodo.org/record/2328952, doi:10.5281/zenodo.2328952.

## Abbreviations

The following abbreviations are used in this manuscript:

MM	Molecular Mechanics
QM	Quantum Mechanics
SQM	Semiempirical Quantum Mechanics
QM/MM	Quantum Mechanical/Molecular Mechanical hybrid methods
SQM/MM	Semiempirical Quantum Mechanical/Molecular Mechanical hybrid methods
FEP	Free Energy Perturbation
BAR	Bennett’s Acceptance Ratio
JAR	Jarzynski’s equation
CRO	Crooks’ equation
