# Supplementary material for: The Good, the Bad, and the Ugly: “HiPen”, a New Dataset for Validating (S)QM/MM Free Energy Simulations
Source: Molecules. 2019 Feb 14;24(4):681. doi: 10.3390/molecules24040681 (PMC6413162; doi:10.3390/molecules24040681)
Supplement: Supplementary file 1 [file molecules-24-00681-s001.zip › final-SI/hipen22.pdf]

Supporting Information for The Good, the Bad, and the Ugly: "Hipen," a new dataset for validating indirect free energy simulations

Fiona L. Kearns,[*,†] Luke Warrensford,[*,†] Stefan Boresch,[*,‡] and H. Lee Woodcock[*,†]

†*Department of Chemistry, University of South Florida, 4202 E. Fowler Ave., CHE205, Tampa, FL 33620-5250, United States*

‡*University of Vienna, Faculty of Chemistry, Department of Computational Biological Chemistry, Waehringerstrasse 17, A-1090, Vienna, Austria*

E-mail: fionakearns@mail.usf.edu; lwarrensford@mail.usf.edu; stefan@mdy.univie.ac.at; hlw@usf.edu

This Supporting Information contains dihedral distribution plots and potential energy overlap plots for all 22 molecules in the HiPen set. Additionally, all topology, minimized coordinates, parameters, and necessary input files can be found at `https://zenodo.org/record/2328952`, doi:10.5281/zenodo.2328952.

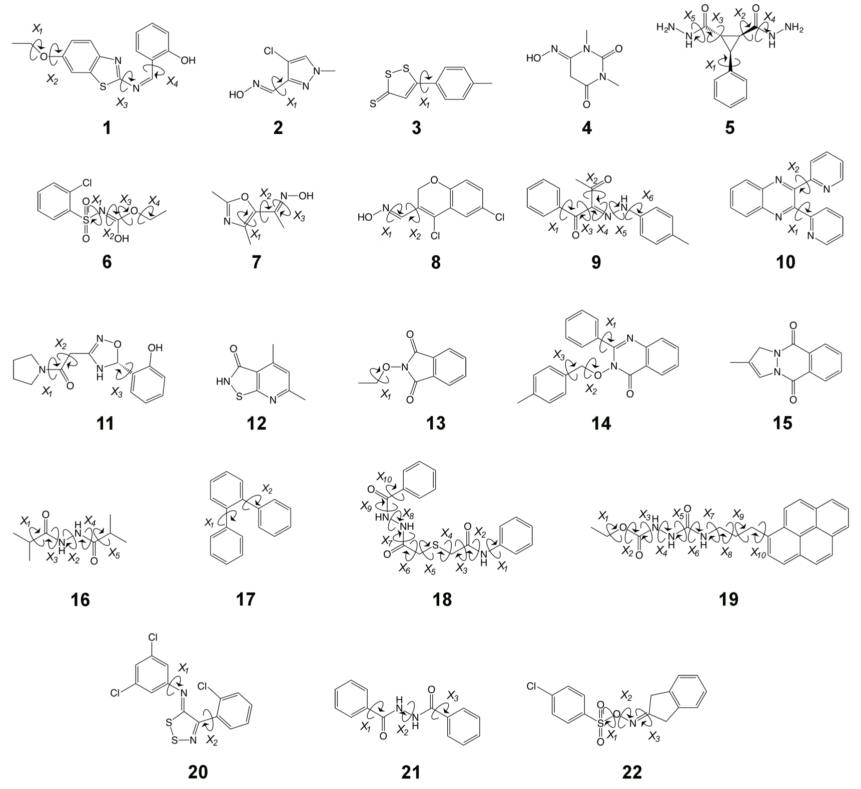

Figure 1: All 22 molecules included in our HiPen testset.

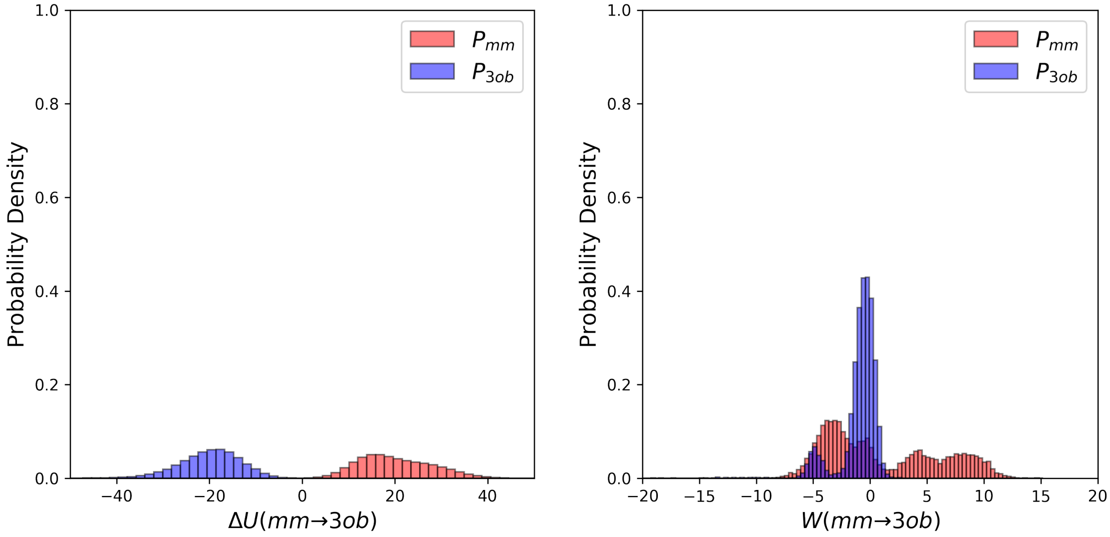

(a) $P_{mm} = p(U^{3ob} - U^{MM})$, $P_{3ob} = -p(U^{MM} - U^{3ob})$ **(b)** $P_{mm} = p(W^{MM\to 3ob})$, $P_{3ob} = -p(W^{3ob\to MM})$

Figure 2: (a) **1**'s potential energy "forward" (P_{mm}) and "backward" (P_{3ob}) distributions plotted as "offset" from the $\overline{\Delta U^{MM\to 3ob}}$ to simplify the x-axis. (b) **1**'s nonequilibrium work "forward" (P_{mm}) and "backward" (P_{3ob}) distributions plotted as "offset" from the $\overline{W^{MM\to 3ob}}$ to simplify the x-axis.

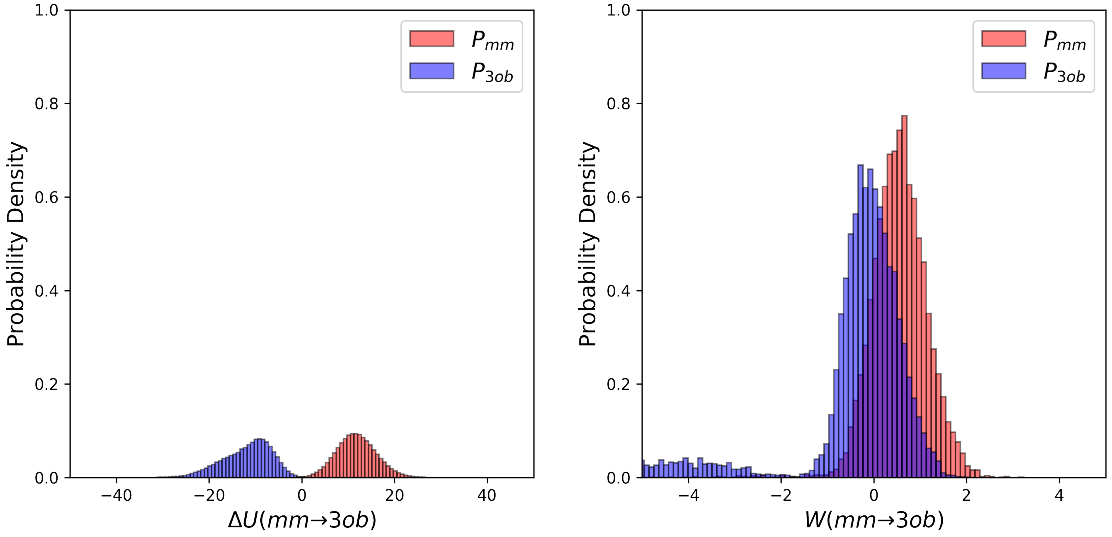

(a) $P_{mm} = p(U^{3ob} - U^{MM})$, $P_{3ob} = -p(U^{MM} - U^{3ob})$ **(b)** $P_{mm} = p(W^{MM\to 3ob})$, $P_{3ob} = -p(W^{3ob\to MM})$

Figure 3: (a) **2**'s potential energy "forward" (P_{mm}) and "backward" (P_{3ob}) distributions plotted as "offset" from the $\overline{\Delta U^{MM\to 3ob}}$ to simplify the x-axis. (b) **2**'s nonequilibrium work "forward" (P_{mm}) and "backward" (P_{3ob}) distributions plotted as "offset" from the $\overline{W^{MM\to 3ob}}$ to simplify the x-axis.

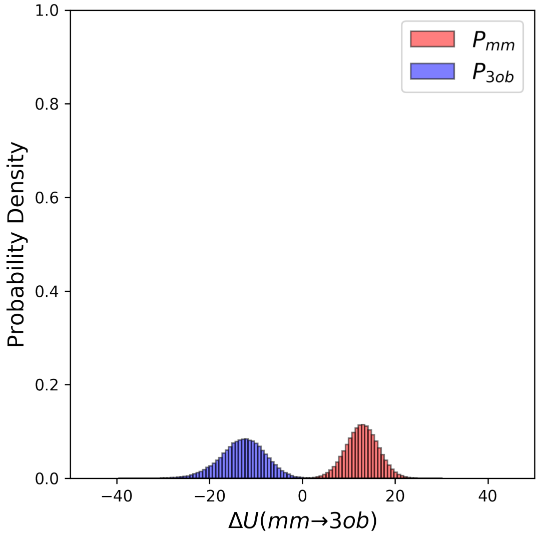

(a) $P_{mm} = p(U^{3ob} - U^{MM})$, $P_{3ob} = -p(U^{MM} - U^{3ob})$

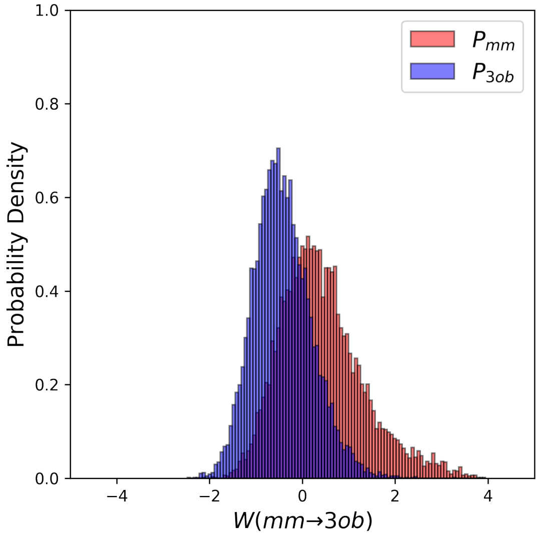

(b) $P_{mm} = p(W^{MM\to 3ob})$, $P_{3ob} = -p(W^{3ob\to MM})$

Figure 4: (a) **3**'s potential energy "forward" (P_{mm}) and "backward" (P_{3ob}) distributions plotted as "offset" from the $\overline{\Delta U^{MM\to 3ob}}$ to simplify the x-axis. (b) **3**'s nonequilibrium work "forward" (P_{mm}) and "backward" (P_{3ob}) distributions plotted as "offset" from the $\overline{W^{MM\to 3ob}}$ to simplify the x-axis.

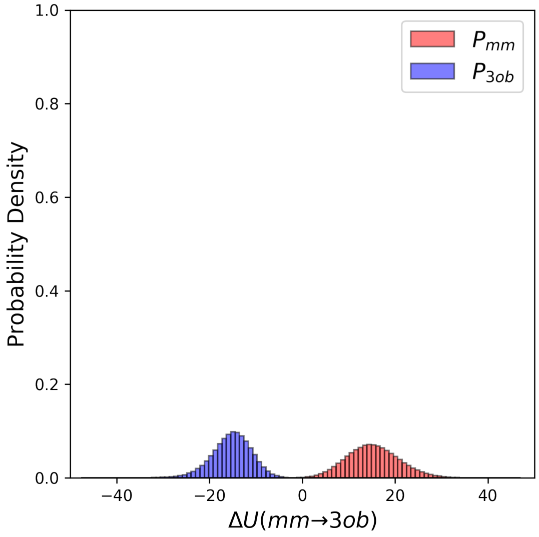

(a) $P_{mm} = p(U^{3ob} - U^{MM})$, $P_{3ob} = -p(U^{MM} - U^{3ob})$

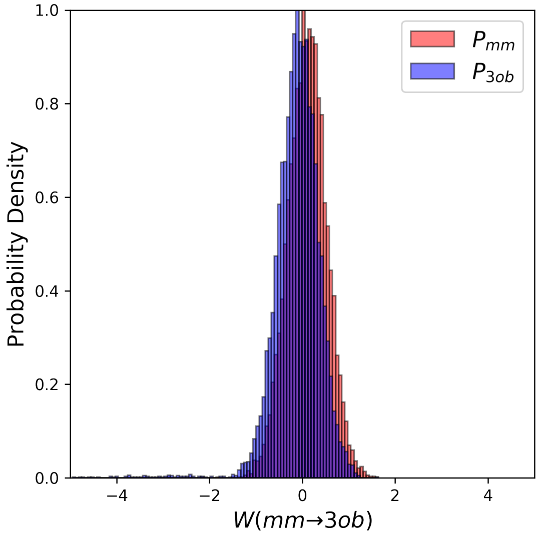

(b) $P_{mm} = p(W^{MM\to 3ob})$, $P_{3ob} = -p(W^{3ob\to MM})$

Figure 5: (a) **4**'s potential energy "forward" (P_{mm}) and "backward" (P_{3ob}) distributions plotted as "offset" from the $\overline{\Delta U^{MM\to 3ob}}$ to simplify the x-axis. (b) **4**'s nonequilibrium work "forward" (P_{mm}) and "backward" (P_{3ob}) distributions plotted as "offset" from the $\overline{W^{MM\to 3ob}}$ to simplify the x-axis.

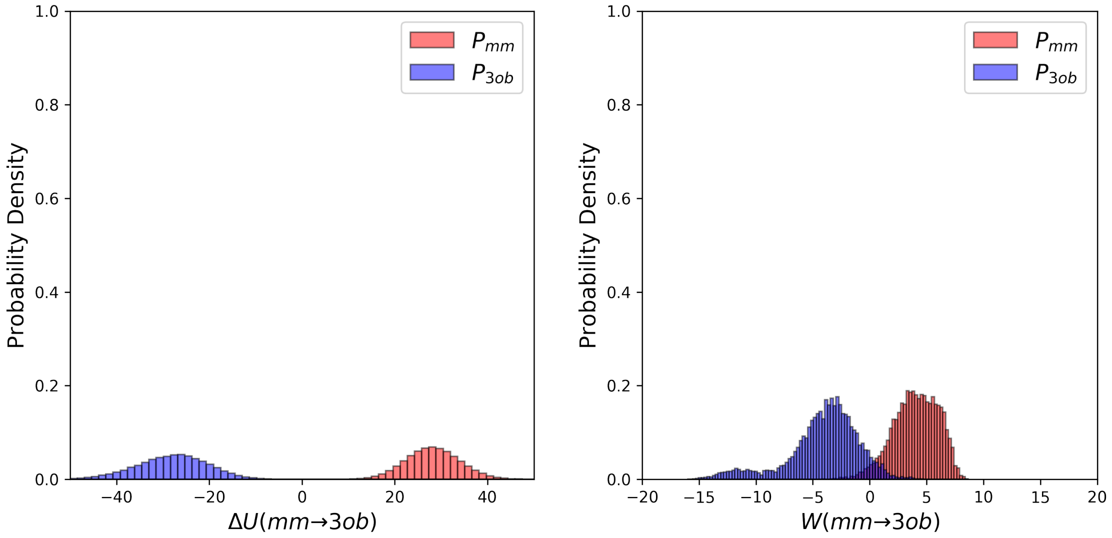

(a) $P_{mm} = p(U^{3ob} - U^{MM})$, $P_{3ob} = -p(U^{MM} - U^{3ob})$ **(b)** $P_{mm} = p(W^{MM\to 3ob})$, $P_{3ob} = -p(W^{3ob\to MM})$

Figure 6: (a) **5**'s potential energy "forward" (P_{mm}) and "backward" (P_{3ob}) distributions plotted as "offset" from the $\overline{\Delta U^{MM\to 3ob}}$ to simplify the x-axis. (b) **5**'s nonequilibrium work "forward" (P_{mm}) and "backward" (P_{3ob}) distributions plotted as "offset" from the $\overline{W^{MM\to 3ob}}$ to simplify the x-axis.

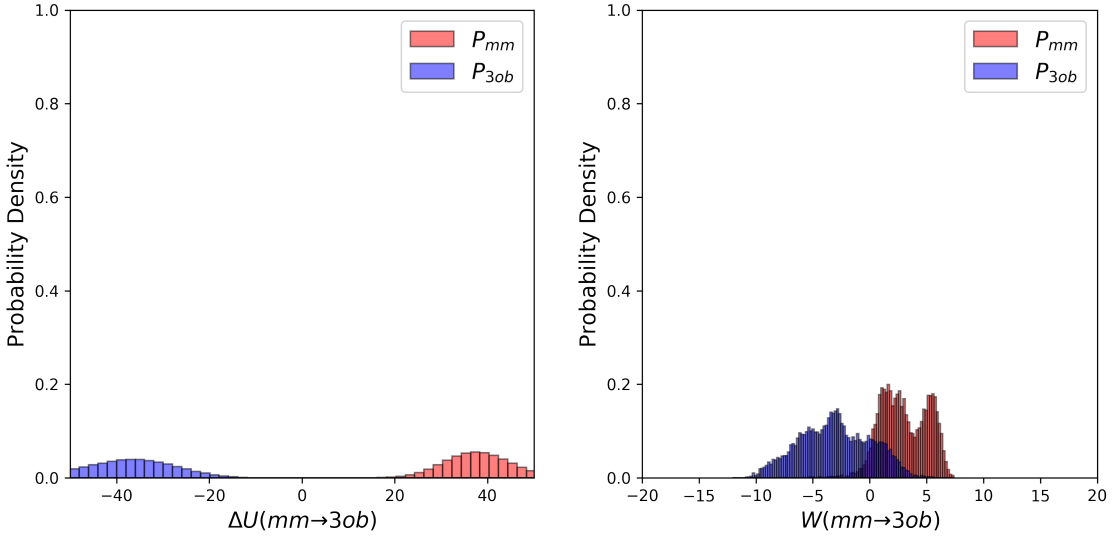

(a) $P_{mm} = p(U^{3ob} - U^{MM})$, $P_{3ob} = -p(U^{MM} - U^{3ob})$ **(b)** $P_{mm} = p(W^{MM\to 3ob})$, $P_{3ob} = -p(W^{3ob\to MM})$

Figure 7: (a) **6**'s potential energy "forward" (P_{mm}) and "backward" (P_{3ob}) distributions plotted as "offset" from the $\overline{\Delta U^{MM\to 3ob}}$ to simplify the x-axis. (b) **6**'s nonequilibrium work "forward" (P_{mm}) and "backward" (P_{3ob}) distributions plotted as "offset" from the $\overline{W^{MM\to 3ob}}$ to simplify the x-axis.

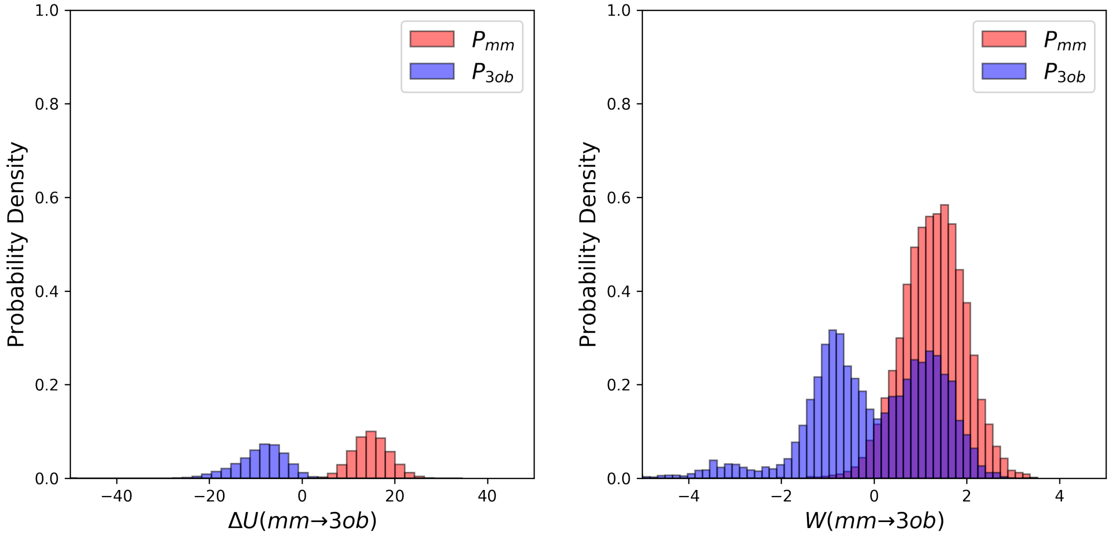

(a) $P_{mm} = p(U^{3ob} - U^{MM})$, $P_{3ob} = -p(U^{MM} - U^{3ob})$ **(b)** $P_{mm} = p(W^{MM\to 3ob})$, $P_{3ob} = -p(W^{3ob\to MM})$

Figure 8: (a) **7**'s potential energy "forward" (P_{mm}) and "backward" (P_{3ob}) distributions plotted as "offset" from the $\overline{\Delta U^{MM\to 3ob}}$ to simplify the x-axis. (b) **7**'s nonequilibrium work "forward" (P_{mm}) and "backward" (P_{3ob}) distributions plotted as "offset" from the $\overline{W^{MM\to 3ob}}$ to simplify the x-axis.

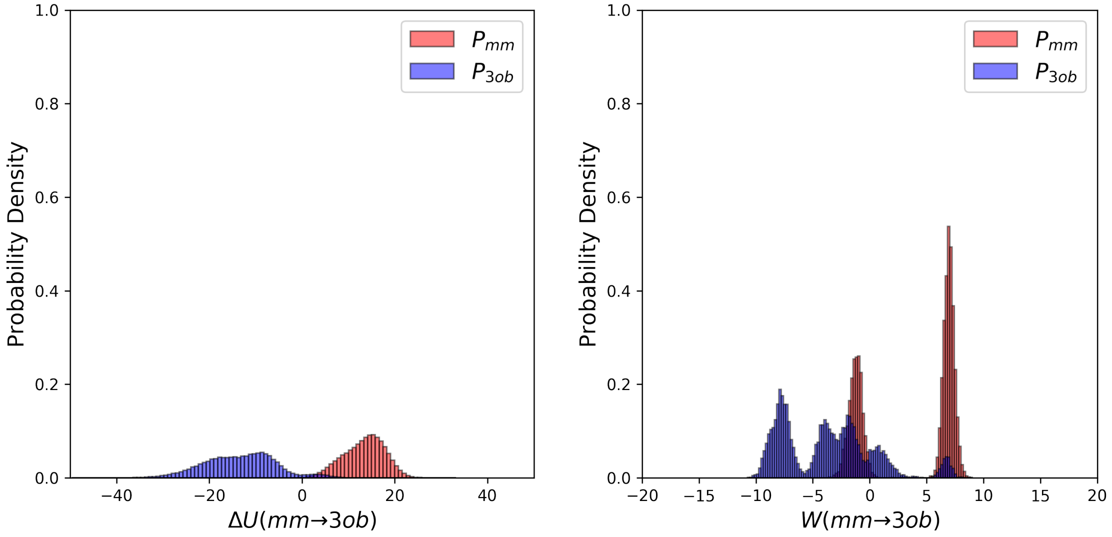

(a) $P_{mm} = p(U^{3ob} - U^{MM})$, $P_{3ob} = -p(U^{MM} - U^{3ob})$ **(b)** $P_{mm} = p(W^{MM\to 3ob})$, $P_{3ob} = -p(W^{3ob\to MM})$

Figure 9: (a) **8**'s potential energy "forward" (P_{mm}) and "backward" (P_{3ob}) distributions plotted as "offset" from the $\overline{\Delta U^{MM\to 3ob}}$ to simplify the x-axis. (b) **8**'s nonequilibrium work "forward" (P_{mm}) and "backward" (P_{3ob}) distributions plotted as "offset" from the $\overline{W^{MM\to 3ob}}$ to simplify the x-axis.

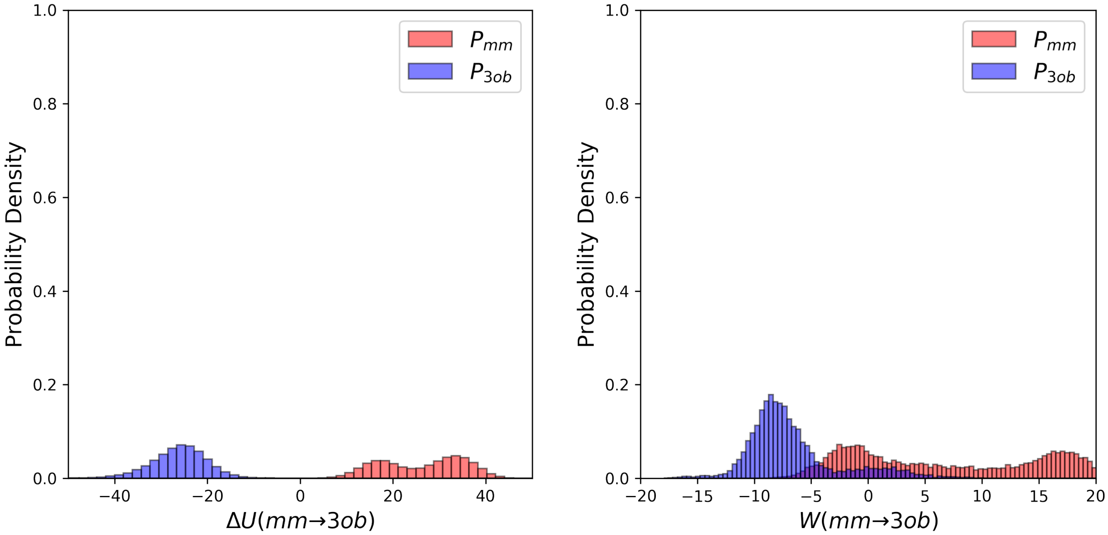

(a) $P_{mm} = p(U^{3ob} - U^{MM})$, $P_{3ob} = -p(U^{MM} - U^{3ob})$ **(b)** $P_{mm} = p(W^{MM\to 3ob})$, $P_{3ob} = -p(W^{3ob\to MM})$

Figure 10: (a) **9**'s potential energy "forward" (P_{mm}) and "backward" (P_{3ob}) distributions plotted as "offset" from the $\overline{\Delta U^{MM\to 3ob}}$ to simplify the x-axis. (b) **9**'s nonequilibrium work "forward" (P_{mm}) and "backward" (P_{3ob}) distributions plotted as "offset" from the $\overline{W^{MM\to 3ob}}$ to simplify the x-axis.

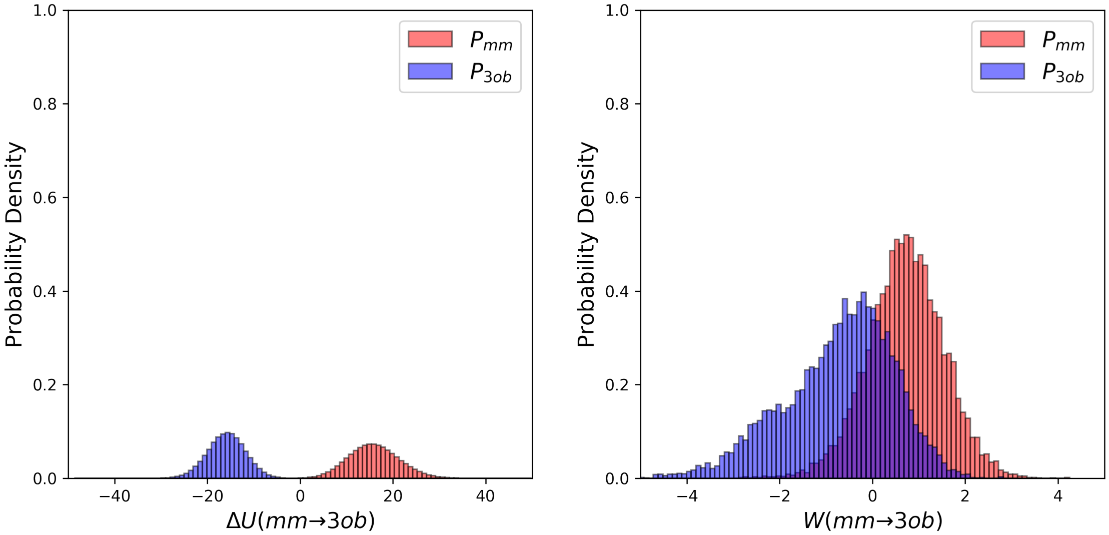

(a) $P_{mm} = p(U^{3ob} - U^{MM})$, $P_{3ob} = -p(U^{MM} - U^{3ob})$ **(b)** $P_{mm} = p(W^{MM\to 3ob})$, $P_{3ob} = -p(W^{3ob\to MM})$

Figure 11: (a) **10**'s potential energy "forward" (P_{mm}) and "backward" (P_{3ob}) distributions plotted as "offset" from the $\overline{\Delta U^{MM\to 3ob}}$ to simplify the x-axis. (b) **10**'s nonequilibrium work "forward" (P_{mm}) and "backward" (P_{3ob}) distributions plotted as "offset" from the $\overline{W^{MM\to 3ob}}$ to simplify the x-axis.

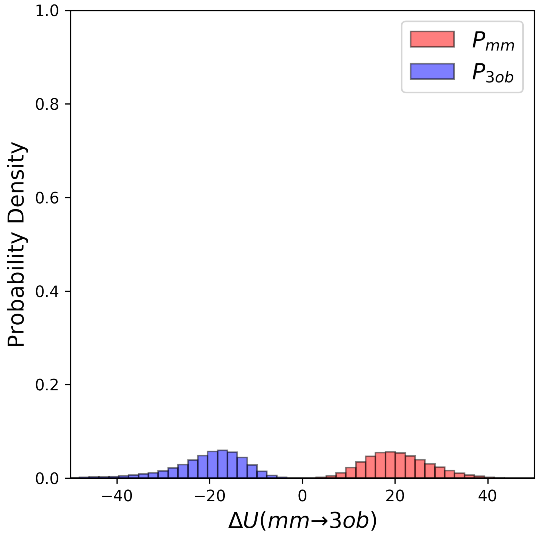

(a) $P_{mm} = p(U^{3ob} - U^{MM})$, $P_{3ob} = -p(U^{MM} - U^{3ob})$

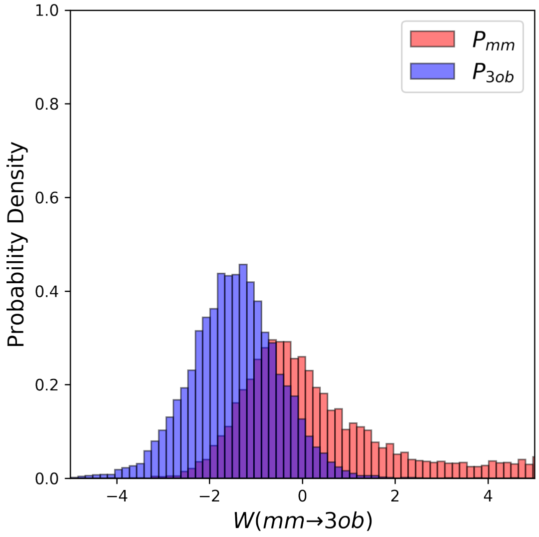

(b) $P_{mm} = p(W^{MM\to 3ob})$, $P_{3ob} = -p(W^{3ob\to MM})$

Figure 12: (a) **11**'s potential energy "forward" (P_{mm}) and "backward" (P_{3ob}) distributions plotted as "offset" from the $\overline{\Delta U^{MM\to 3ob}}$ to simplify the x-axis. (b) **11**'s nonequilibrium work "forward" (P_{mm}) and "backward" (P_{3ob}) distributions plotted as "offset" from the $\overline{W^{MM\to 3ob}}$ to simplify the x-axis.

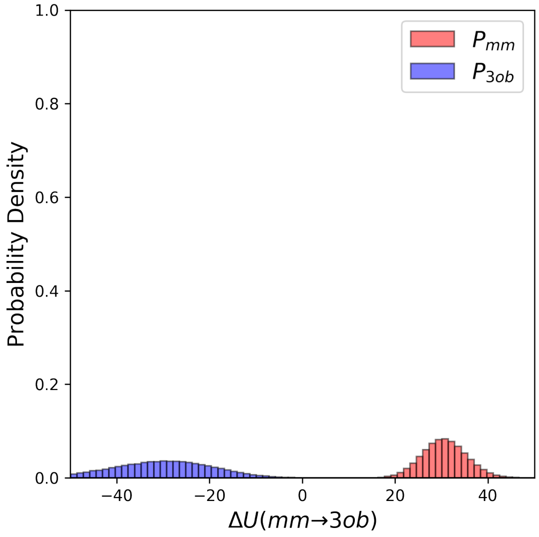

(a) $P_{mm} = p(U^{3ob} - U^{MM})$, $P_{3ob} = -p(U^{MM} - U^{3ob})$

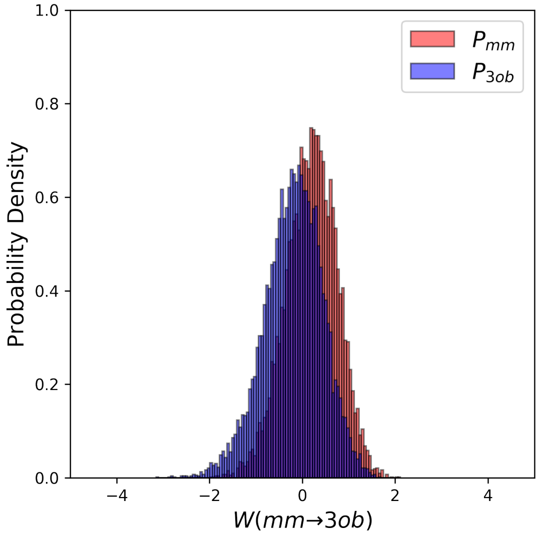

(b) $P_{mm} = p(W^{MM\to 3ob})$, $P_{3ob} = -p(W^{3ob\to MM})$

Figure 13: (a) **12**'s potential energy "forward" (P_{mm}) and "backward" (P_{3ob}) distributions plotted as "offset" from the $\overline{\Delta U^{MM\to 3ob}}$ to simplify the x-axis. (b) **12**'s nonequilibrium work "forward" (P_{mm}) and "backward" (P_{3ob}) distributions plotted as "offset" from the $\overline{W^{MM\to 3ob}}$ to simplify the x-axis.

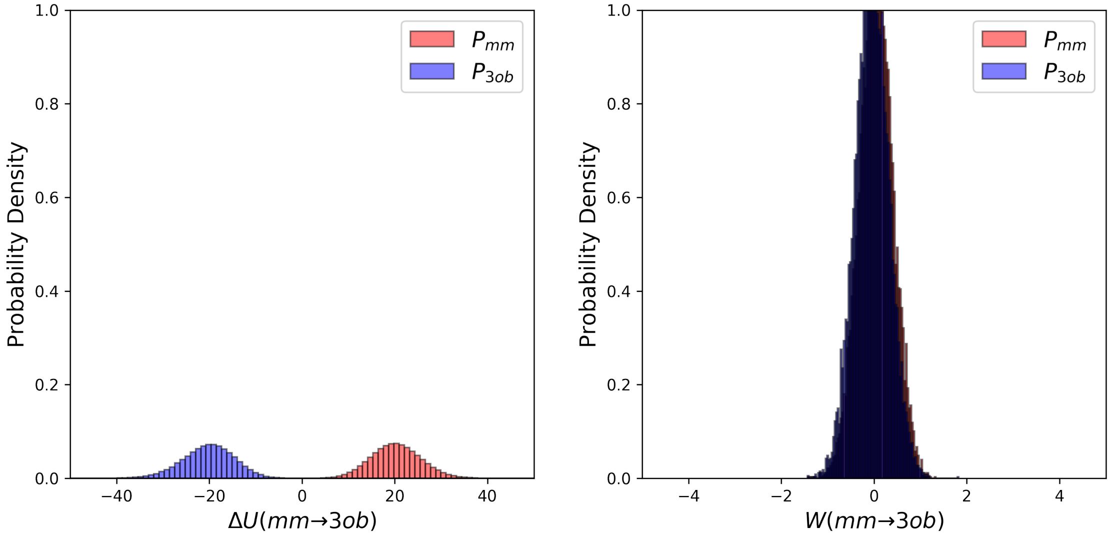

(a) $P_{mm} = p(U^{3ob} - U^{MM})$, $P_{3ob} = -p(U^{MM} - U^{3ob})$ **(b)** $P_{mm} = p(W^{MM \to 3ob})$, $P_{3ob} = -p(W^{3ob \to MM})$

Figure 14: (a) **13**'s potential energy "forward" (P_{mm}) and "backward" (P_{3ob}) distributions plotted as "offset" from the $\overline{\Delta U^{MM \to 3ob}}$ to simplify the x-axis. (b) **13**'s nonequilibrium work "forward" (P_{mm}) and "backward" (P_{3ob}) distributions plotted as "offset" from the $\overline{W^{MM \to 3ob}}$ to simplify the x-axis.

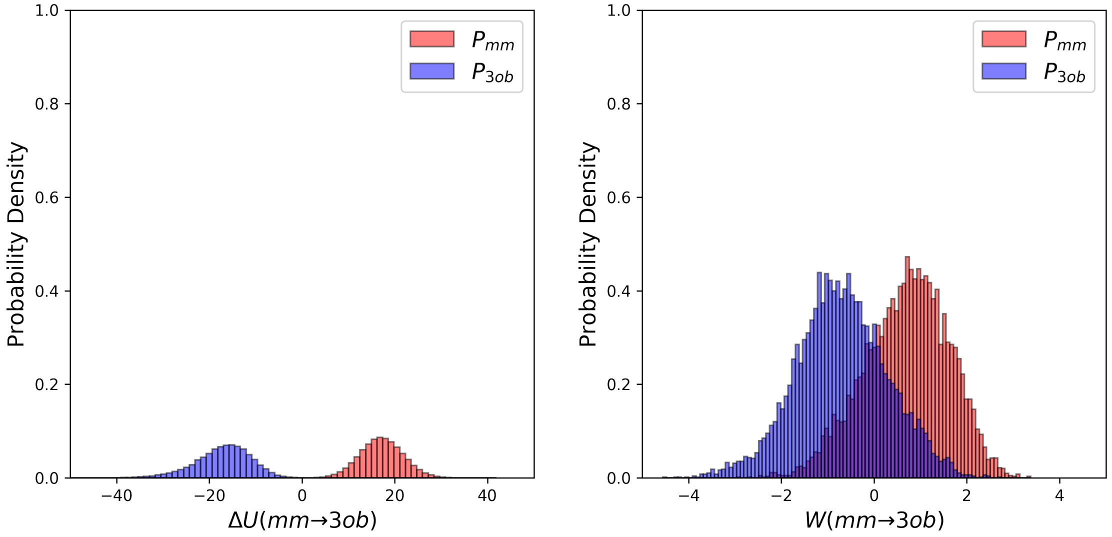

(a) $P_{mm} = p(U^{3ob} - U^{MM})$, $P_{3ob} = -p(U^{MM} - U^{3ob})$ **(b)** $P_{mm} = p(W^{MM \to 3ob})$, $P_{3ob} = -p(W^{3ob \to MM})$

Figure 15: (a) **14**'s potential energy "forward" (P_{mm}) and "backward" (P_{3ob}) distributions plotted as "offset" from the $\overline{\Delta U^{MM \to 3ob}}$ to simplify the x-axis. (b) **14**'s nonequilibrium work "forward" (P_{mm}) and "backward" (P_{3ob}) distributions plotted as "offset" from the $\overline{W^{MM \to 3ob}}$ to simplify the x-axis.

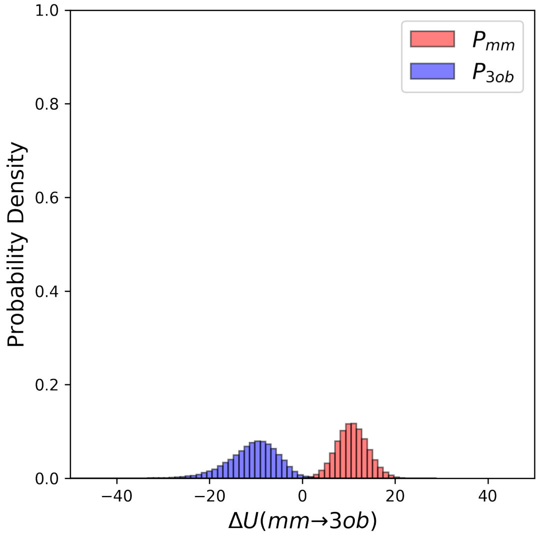

(a) $P_{mm} = p(U^{3ob} - U^{MM})$, $P_{3ob} = -p(U^{MM} - U^{3ob})$

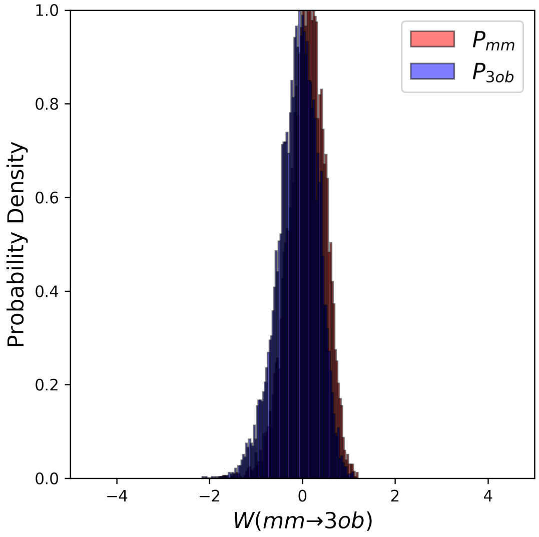

(b) $P_{mm} = p(W^{MM \to 3ob})$, $P_{3ob} = -p(W^{3ob \to MM})$

Figure 16: (a) **15**'s potential energy "forward" (P_{mm}) and "backward" (P_{3ob}) distributions plotted as "offset" from the $\overline{\Delta U^{MM \to 3ob}}$ to simplify the x-axis. (b) **15**'s nonequilibrium work "forward" (P_{mm}) and "backward" (P_{3ob}) distributions plotted as "offset" from the $\overline{W^{MM \to 3ob}}$ to simplify the x-axis.

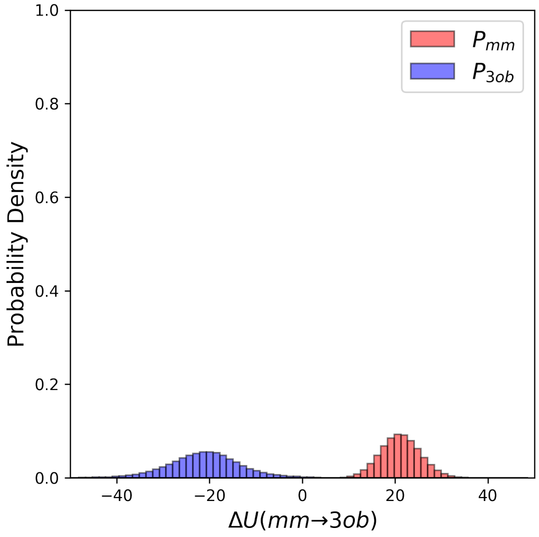

(a) $P_{mm} = p(U^{3ob} - U^{MM})$, $P_{3ob} = -p(U^{MM} - U^{3ob})$

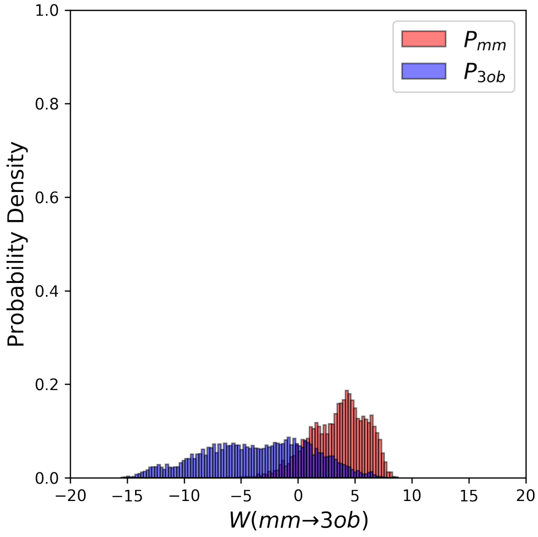

(b) $P_{mm} = p(W^{MM \to 3ob})$, $P_{3ob} = -p(W^{3ob \to MM})$

Figure 17: (a) **16**'s potential energy "forward" (P_{mm}) and "backward" (P_{3ob}) distributions plotted as "offset" from the $\overline{\Delta U^{MM \to 3ob}}$ to simplify the x-axis. (b) **16**'s nonequilibrium work "forward" (P_{mm}) and "backward" (P_{3ob}) distributions plotted as "offset" from the $\overline{W^{MM \to 3ob}}$ to simplify the x-axis.

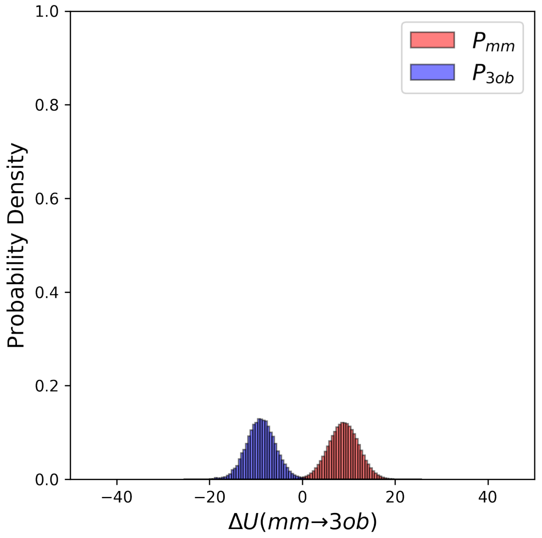

(a) $P_{mm} = p(U^{3ob} - U^{MM})$, $P_{3ob} = -p(U^{MM} - U^{3ob})$

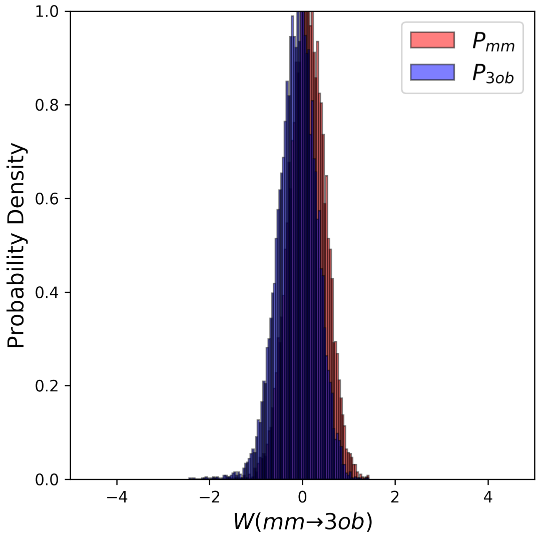

(b) $P_{mm} = p(W^{MM \to 3ob})$, $P_{3ob} = -p(W^{3ob \to MM})$

Figure 18: (a) **17**'s potential energy "forward" (P_{mm}) and "backward" (P_{3ob}) distributions plotted as "offset" from the $\overline{\Delta U^{MM \to 3ob}}$ to simplify the x-axis. (b) **17**'s nonequilibrium work "forward" (P_{mm}) and "backward" (P_{3ob}) distributions plotted as "offset" from the $\overline{W^{MM \to 3ob}}$ to simplify the x-axis.

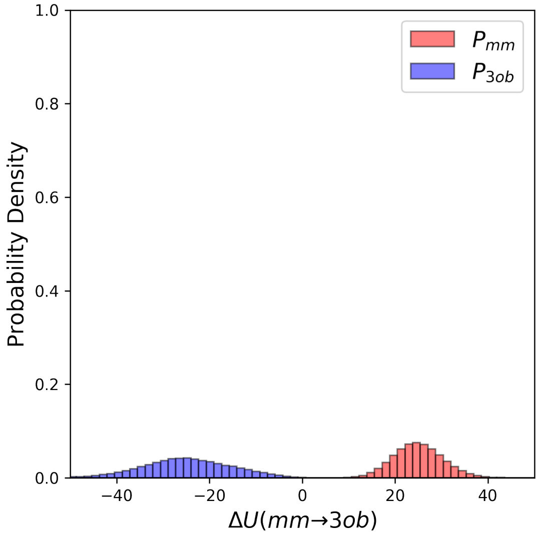

(a) $P_{mm} = p(U^{3ob} - U^{MM})$, $P_{3ob} = -p(U^{MM} - U^{3ob})$

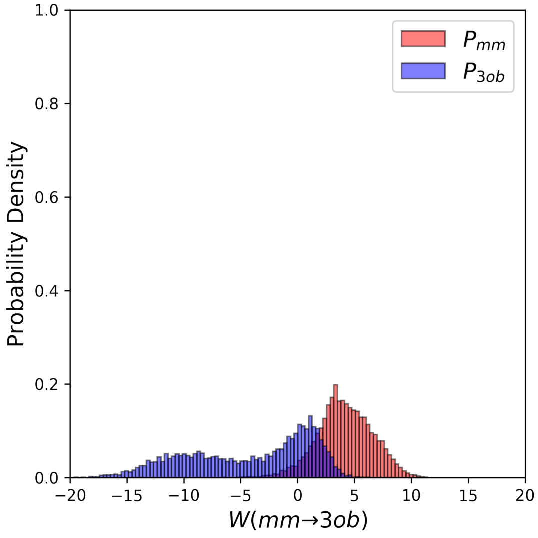

(b) $P_{mm} = p(W^{MM \to 3ob})$, $P_{3ob} = -p(W^{3ob \to MM})$

Figure 19: (a) **18**'s potential energy "forward" (P_{mm}) and "backward" (P_{3ob}) distributions plotted as "offset" from the $\overline{\Delta U^{MM \to 3ob}}$ to simplify the x-axis. (b) **18**'s nonequilibrium work "forward" (P_{mm}) and "backward" (P_{3ob}) distributions plotted as "offset" from the $\overline{W^{MM \to 3ob}}$ to simplify the x-axis.

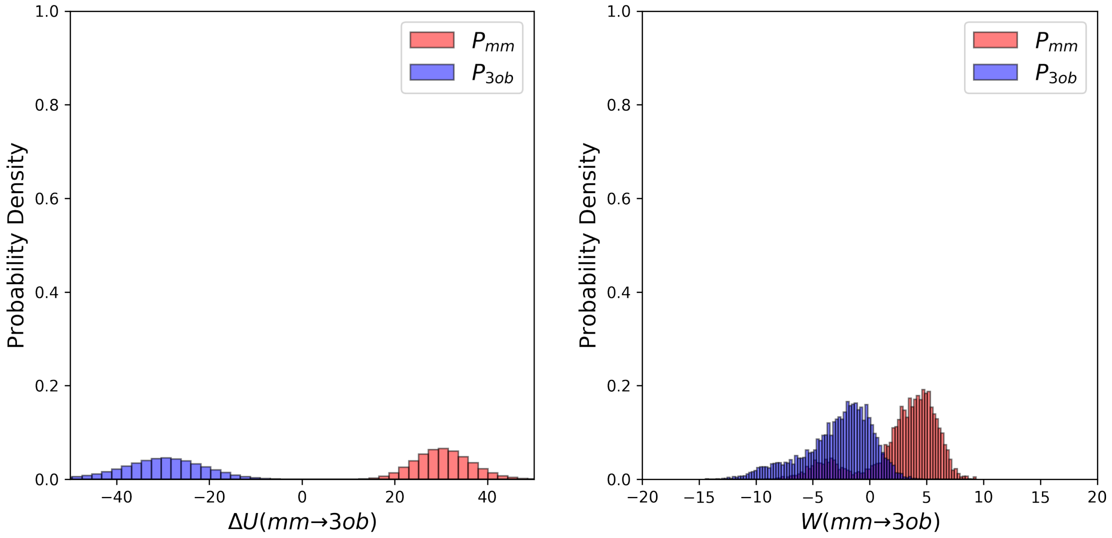

(a) $P_{mm} = p(U^{3ob} - U^{MM})$, $P_{3ob} = -p(U^{MM} - U^{3ob})$ **(b)** $P_{mm} = p(W^{MM \to 3ob})$, $P_{3ob} = -p(W^{3ob \to MM})$

Figure 20: (a) **19**'s potential energy "forward" (P_{mm}) and "backward" (P_{3ob}) distributions plotted as "offset" from the $\overline{\Delta U^{MM \to 3ob}}$ to simplify the x-axis. (b) **19**'s nonequilibrium work "forward" (P_{mm}) and "backward" (P_{3ob}) distributions plotted as "offset" from the $\overline{W^{MM \to 3ob}}$ to simplify the x-axis.

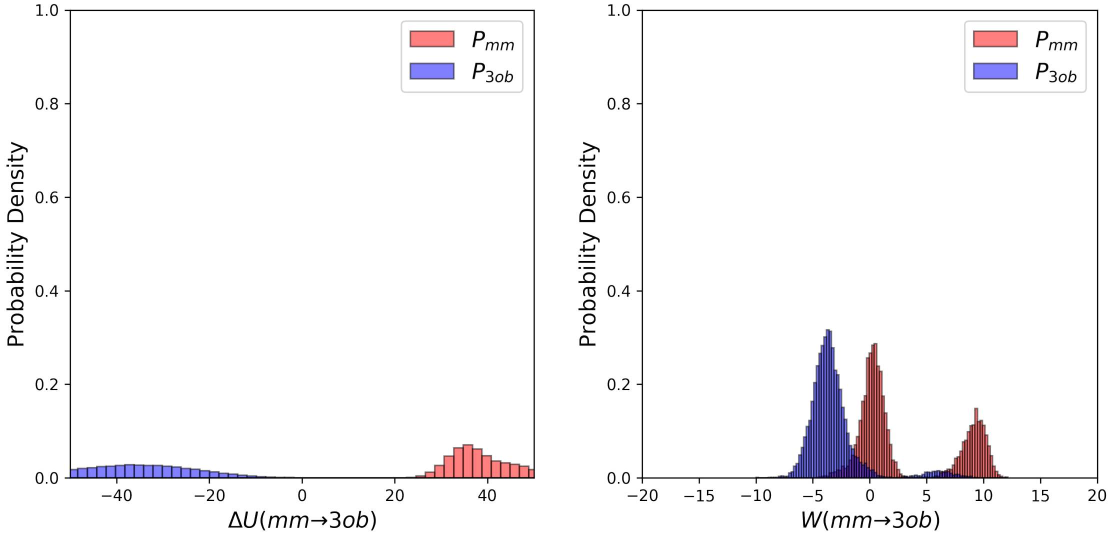

(a) $P_{mm} = p(U^{3ob} - U^{MM})$, $P_{3ob} = -p(U^{MM} - U^{3ob})$ **(b)** $P_{mm} = p(W^{MM \to 3ob})$, $P_{3ob} = -p(W^{3ob \to MM})$

Figure 21: (a) **20**'s potential energy "forward" (P_{mm}) and "backward" (P_{3ob}) distributions plotted as "offset" from the $\overline{\Delta U^{MM \to 3ob}}$ to simplify the x-axis. (b) **20**'s nonequilibrium work "forward" (P_{mm}) and "backward" (P_{3ob}) distributions plotted as "offset" from the $\overline{W^{MM \to 3ob}}$ to simplify the x-axis.

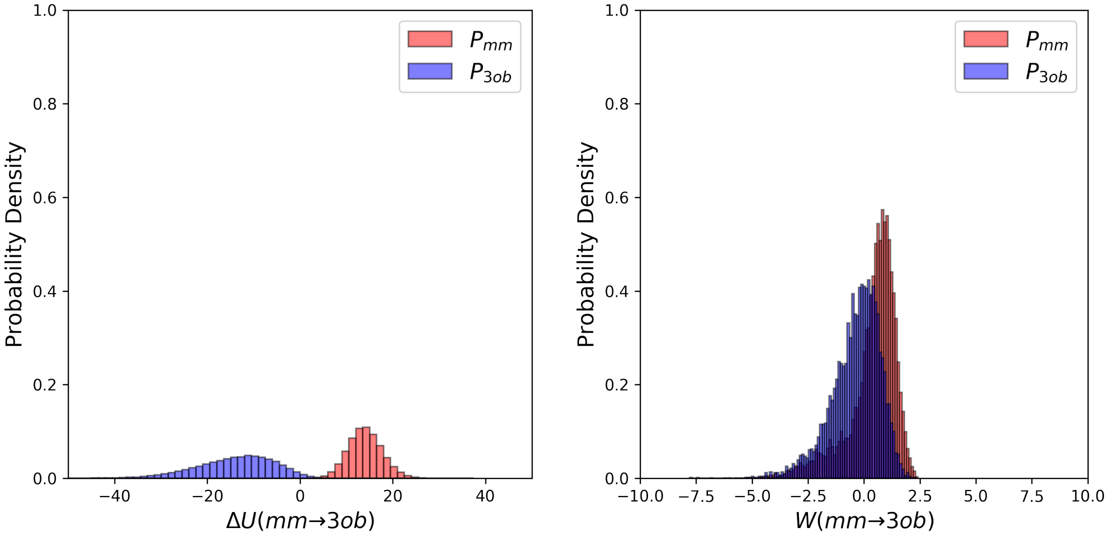

(a) $P_{mm} = p(U^{3ob} - U^{MM})$, $P_{3ob} = -p(U^{MM} - U^{3ob})$ **(b)** $P_{mm} = p(W^{MM \rightarrow 3ob})$, $P_{3ob} = -p(W^{3ob \rightarrow MM})$

Figure 22: (a) **21**'s potential energy "forward" (P_{mm}) and "backward" (P_{3ob}) distributions plotted as "offset" from the $\overline{\Delta U^{MM \rightarrow 3ob}}$ to simplify the x-axis. (b) **21**'s nonequilibrium work "forward" (P_{mm}) and "backward" (P_{3ob}) distributions plotted as "offset" from the $\overline{W^{MM \rightarrow 3ob}}$ to simplify the x-axis.

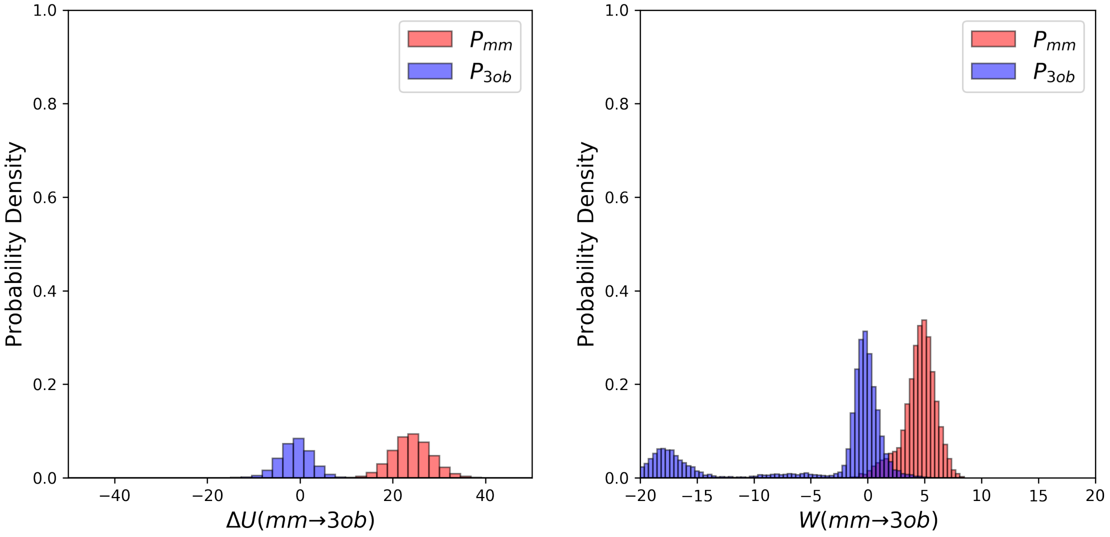

(a) $P_{mm} = p(U^{3ob} - U^{MM})$, $P_{3ob} = -p(U^{MM} - U^{3ob})$ **(b)** $P_{mm} = p(W^{MM \rightarrow 3ob})$, $P_{3ob} = -p(W^{3ob \rightarrow MM})$

Figure 23: (a) **22**'s potential energy "forward" (P_{mm}) and "backward" (P_{3ob}) distributions plotted as "offset" from the $\overline{\Delta U^{MM \rightarrow 3ob}}$ to simplify the x-axis. (b) **22**'s nonequilibrium work "forward" (P_{mm}) and "backward" (P_{3ob}) distributions plotted as "offset" from the $\overline{W^{MM \rightarrow 3ob}}$ to simplify the x-axis.

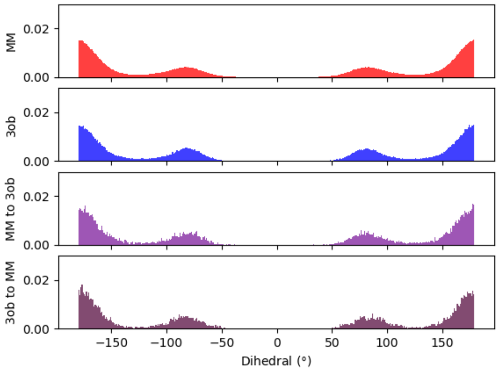

(a) χ_1

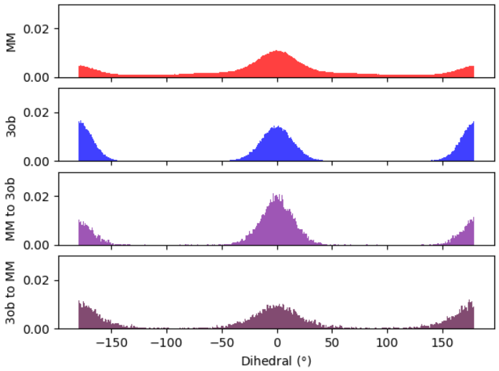

(b) χ_2

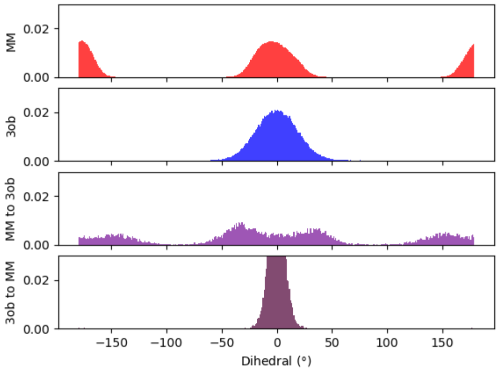

(c) χ_3

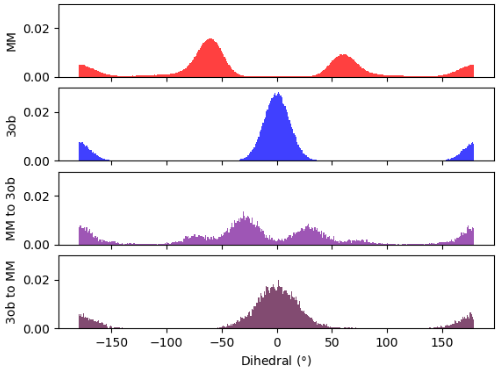

(d) χ_4

Figure 24: Dihedral distributions of molecule **1**.

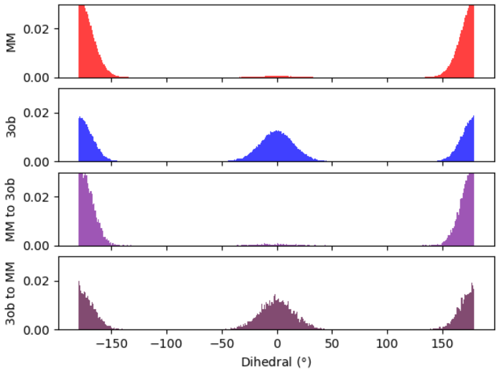

(a) χ_1

Figure 25: Dihedral distributions of molecule **2**.

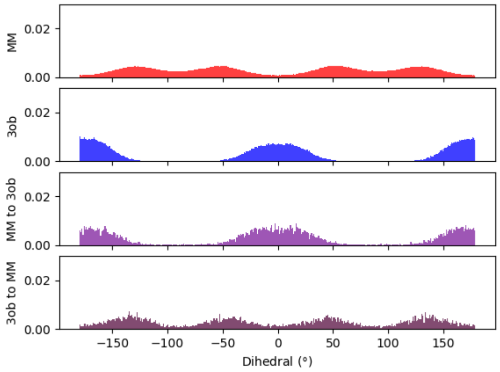

(a) χ_1

Figure 26: Dihedral distributions of molecule **3**.

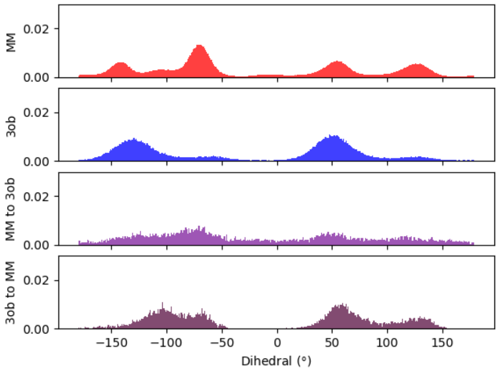

(a) χ_1

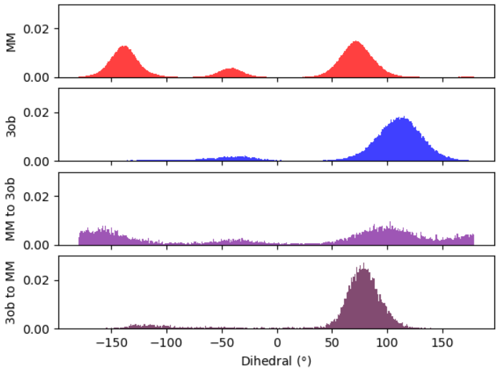

(b) χ_2

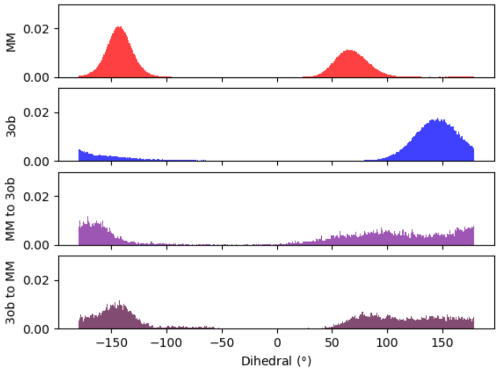

(c) χ_3

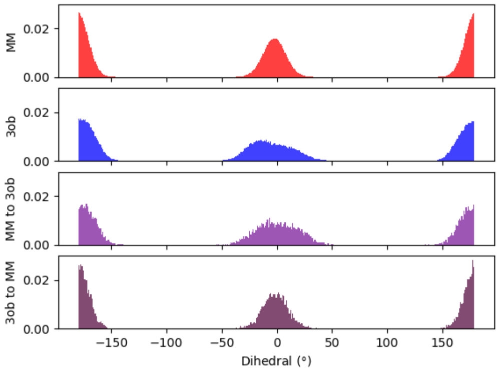

(d) χ_4

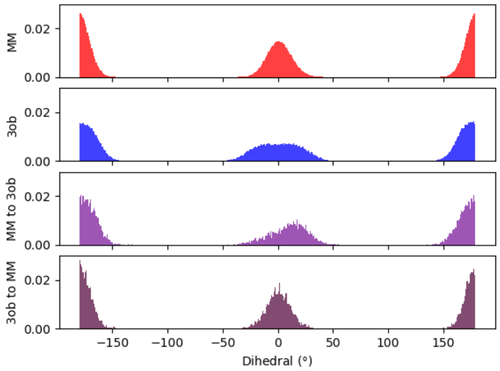

(e) χ_5

Figure 27: Dihedral distributions of molecule **5**.

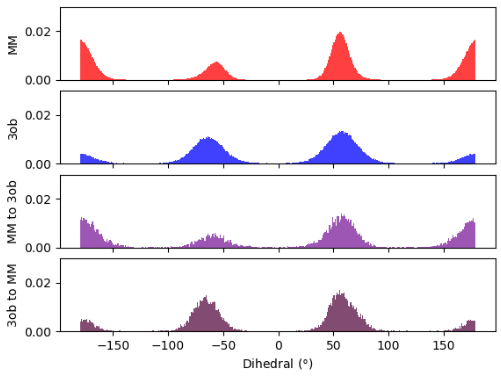

(a) χ_1

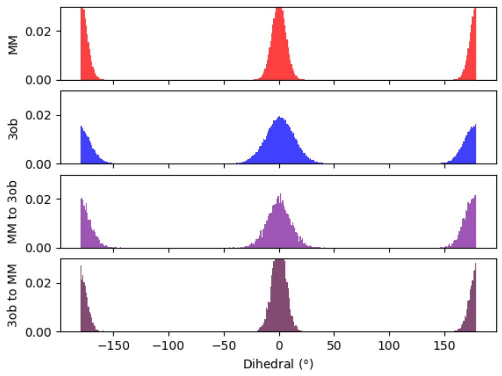

(b) χ_2

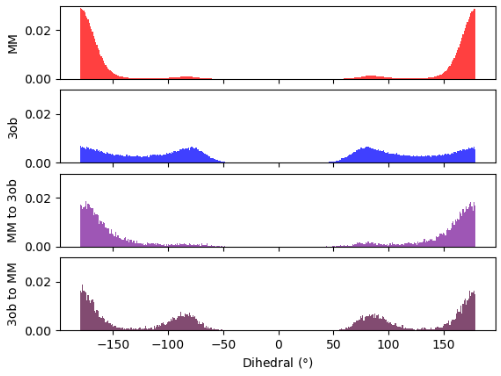

(c) χ_3

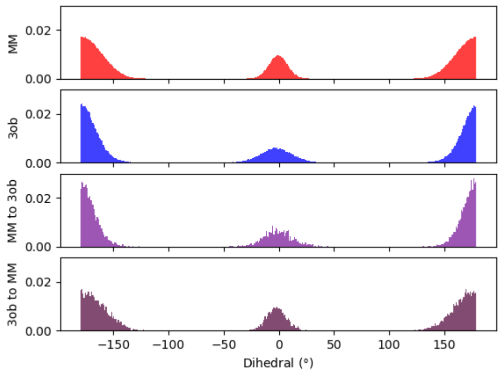

(d) χ_4

Figure 28: Dihedral distributions of molecule **6**.

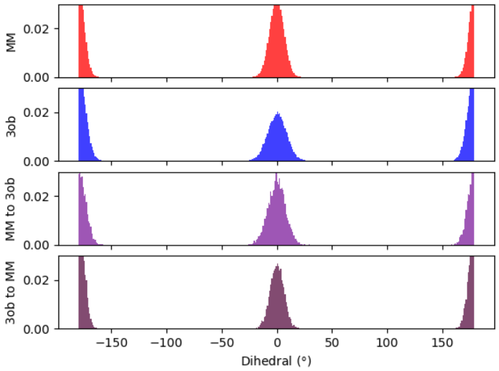

(a) χ_1

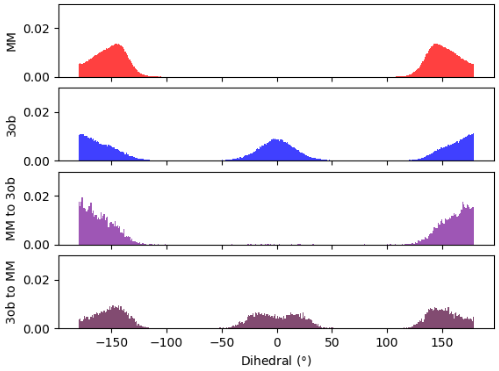

(b) χ_2

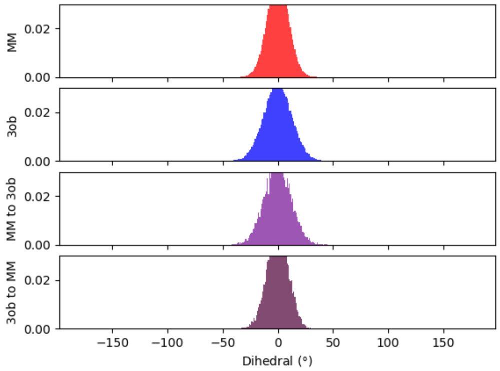

(c) χ_3

Figure 29: Dihedral distributions of molecule **7**.

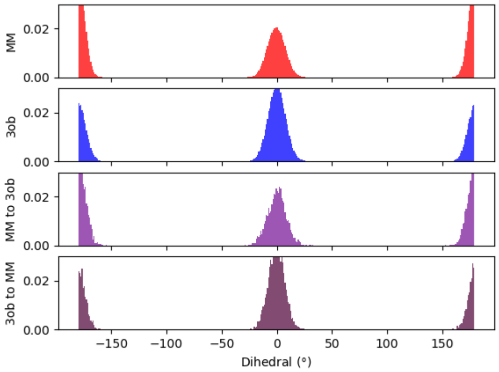

(a) χ_1

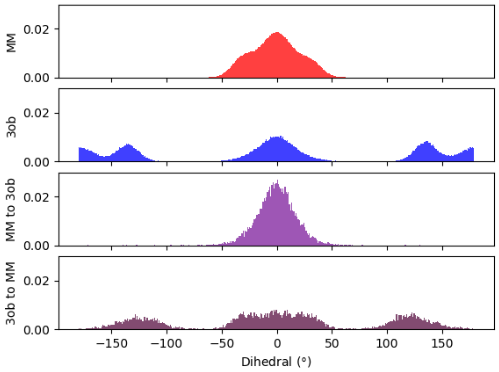

(b) χ_2

Figure 30: Dihedral distributions of molecule **8**.

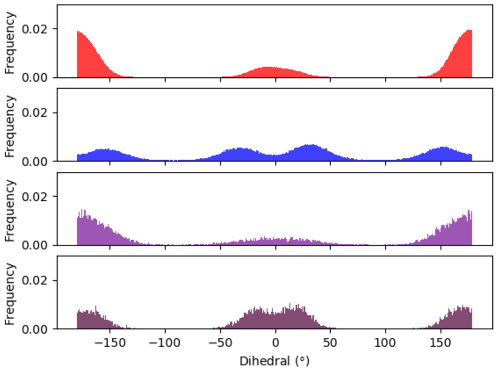

(a) χ_1

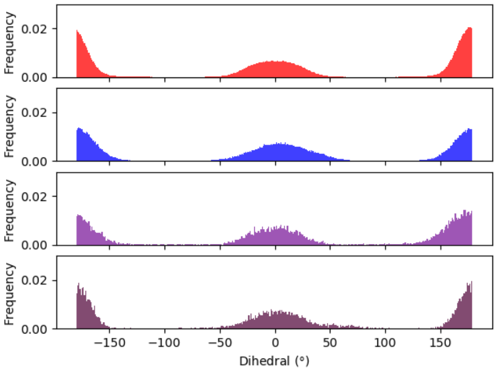

(b) χ_2

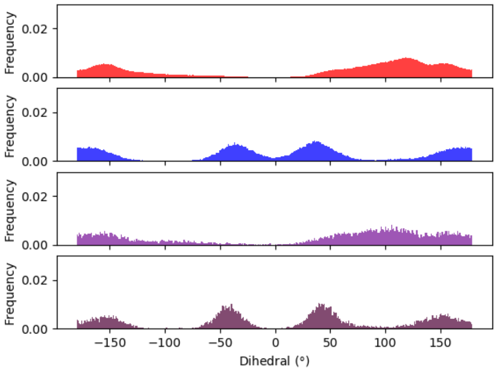

(c) χ_3

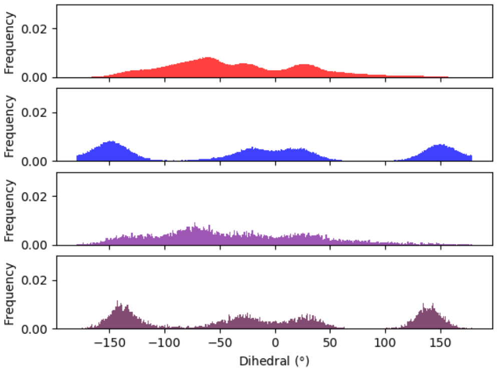

(d) χ_4

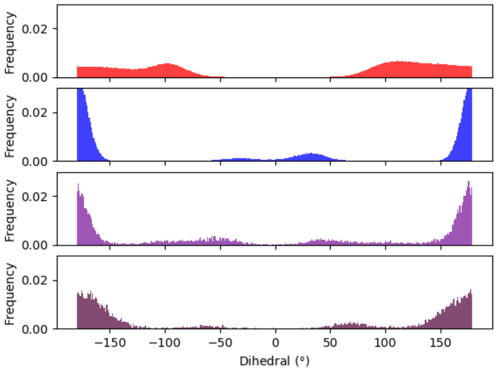

(e) χ_5

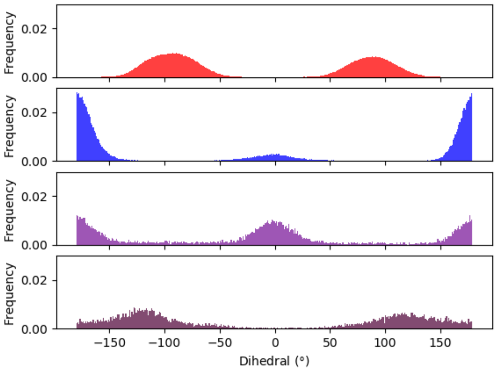

(f) χ_6

Figure 31: Dihedral distributions of molecule **9**.

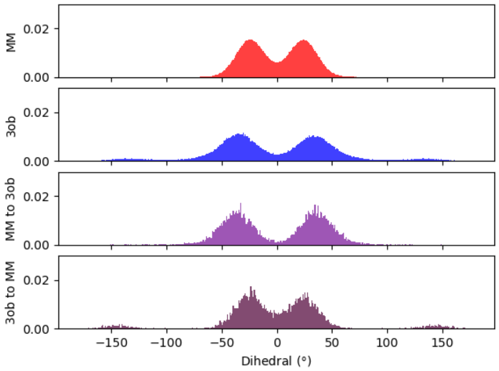

(a) χ_1

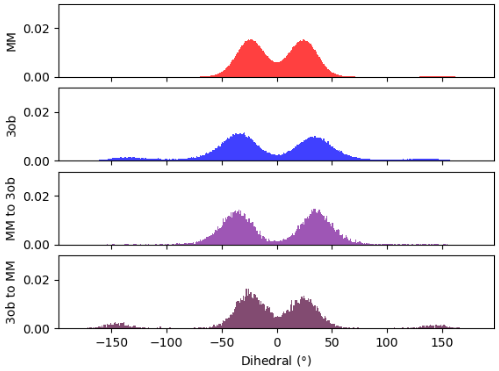

(b) χ_2

Figure 32: Dihedral distributions of molecule **10**.

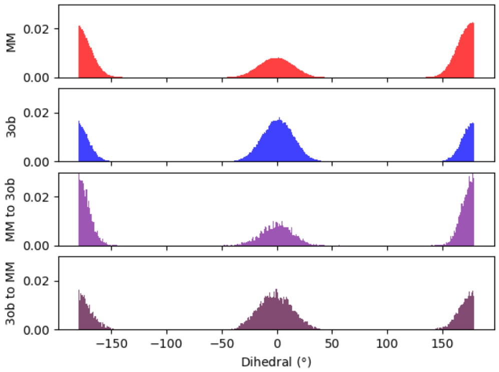

(a) χ_1

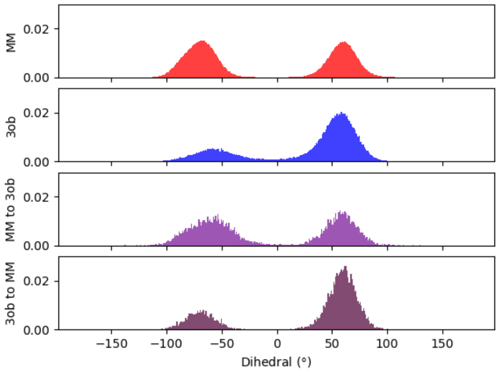

(b) χ_2

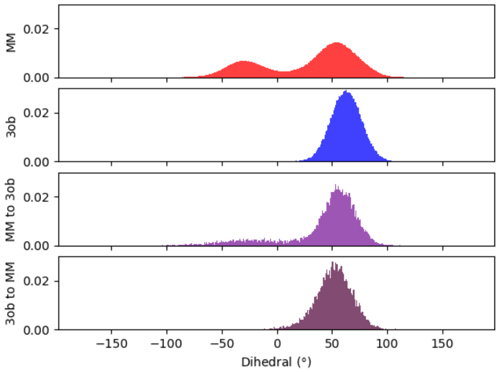

(c) χ_3

Figure 33: Dihedral distributions of molecule **11**.

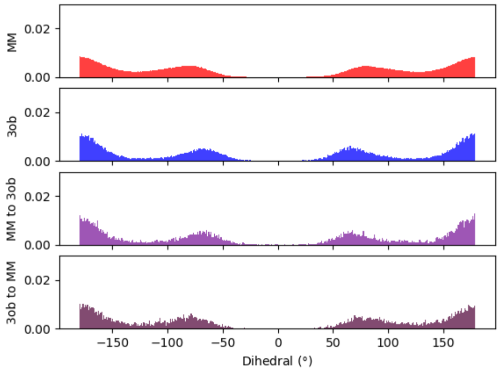

(a) χ_1

Figure 34: Dihedral distributions of molecule **13**.

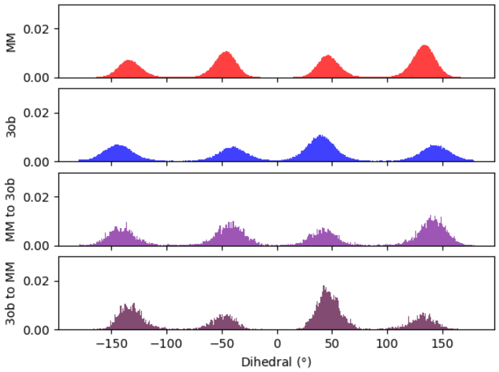

(a) χ_1

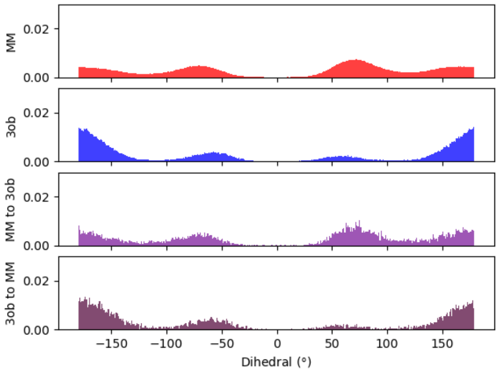

(b) χ_2

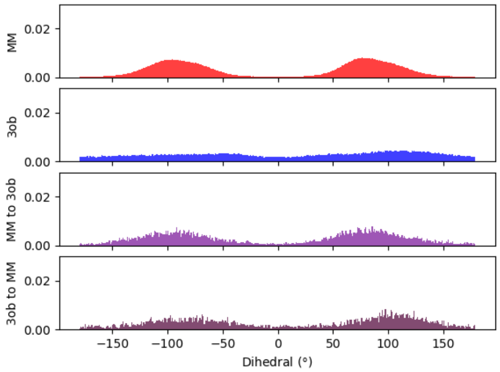

(c) χ_3

Figure 35: Dihedral distributions of molecule **14**.

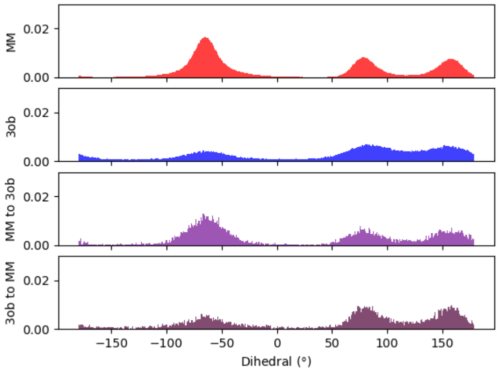

(a) χ_1

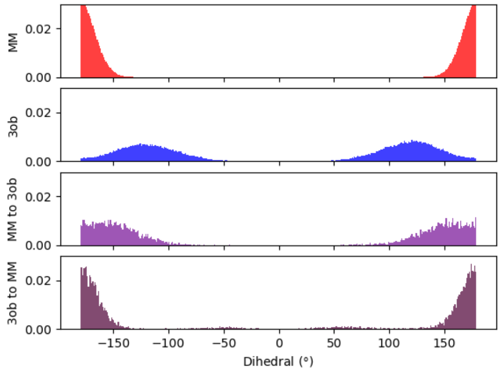

(b) χ_2

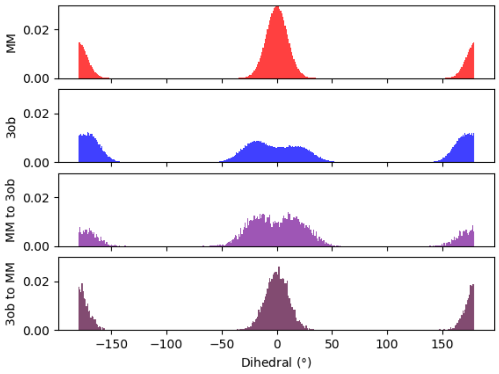

(c) χ_3

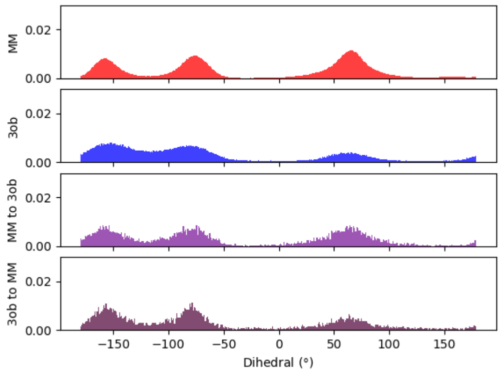

(d) χ_4

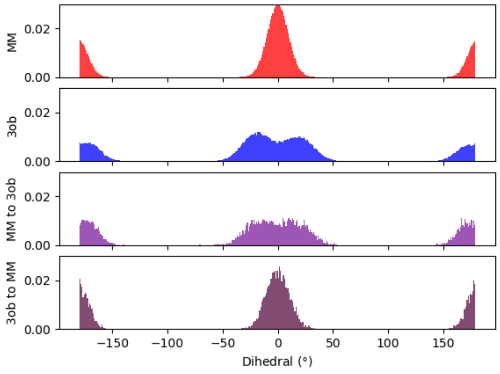

(e) χ_5

Figure 36: Dihedral distributions of molecule **16**.

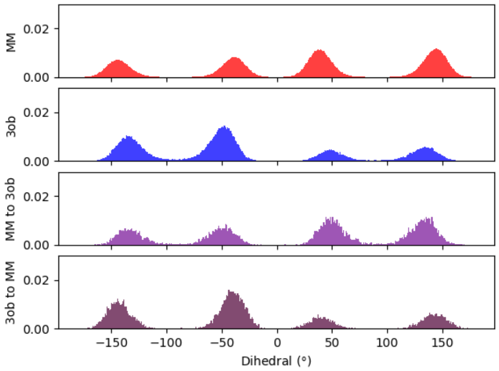

(a) χ_1

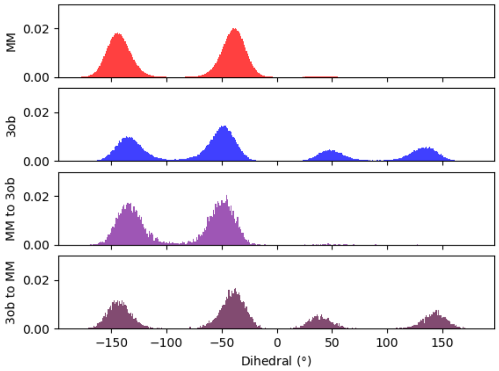

(b) χ_2

Figure 37: Dihedral distributions of molecule **17**.

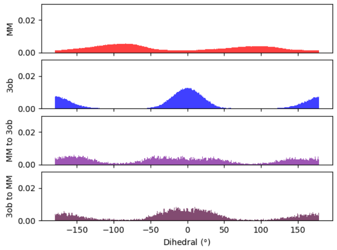

(a) χ_1

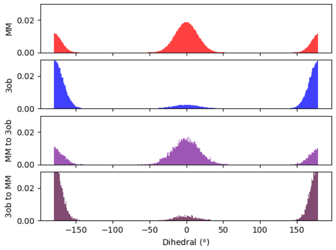

(b) χ_2

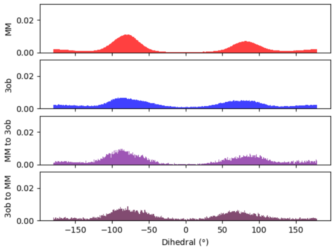

(c) χ_3

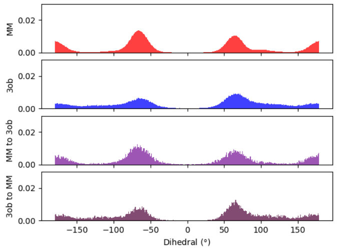

(d) χ_4

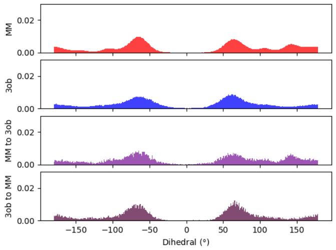

(e) χ_5

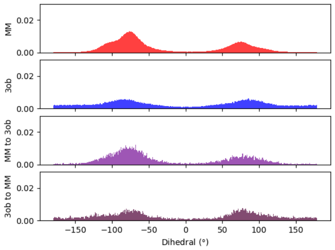

(f) χ_6

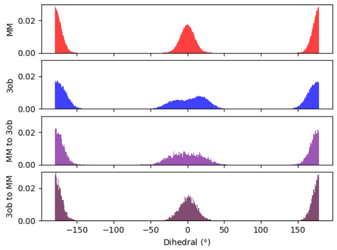

(g) χ_7

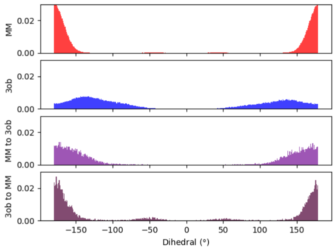

(h) χ_8

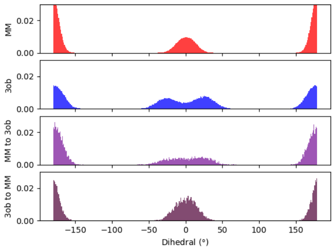

(i) χ_9

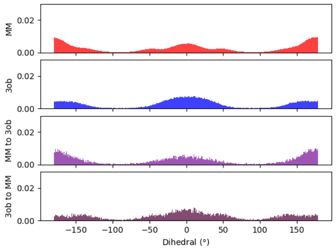

(j) $\chi_1 0$

Figure 38: Dihedral distributions of molecule **18**.

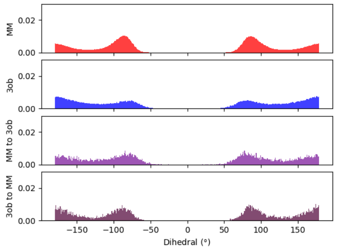

(a) χ_1

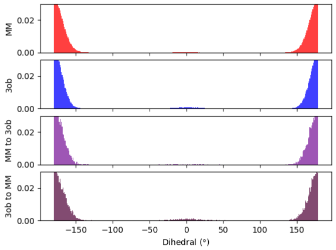

(b) χ_2

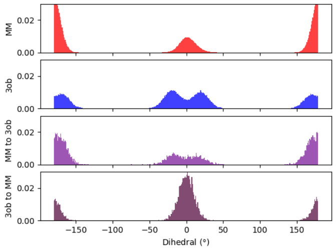

(c) χ_3

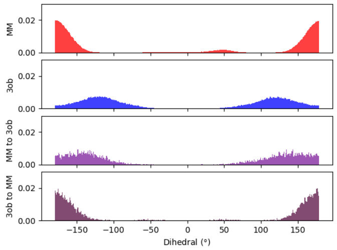

(d) χ_4

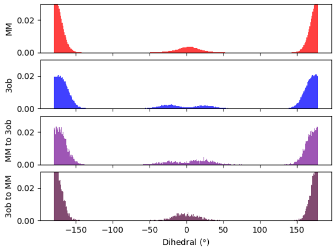

(e) χ_5

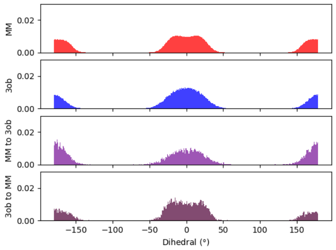

(f) χ_6

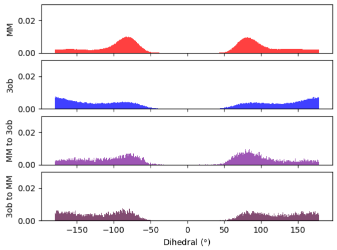

(g) χ_7

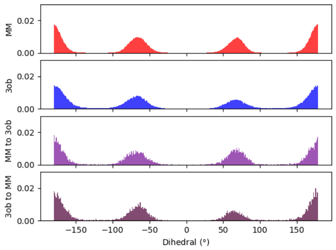

(h) χ_8

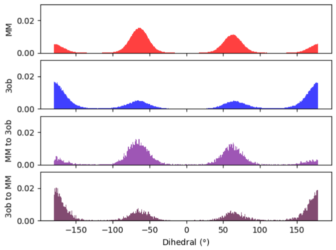

(i) χ_9

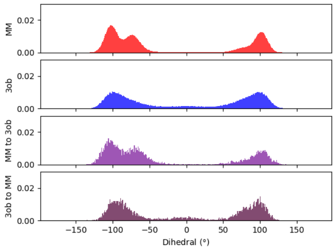

(j) χ_10

Figure 39: Dihedral distributions of molecule **19**.

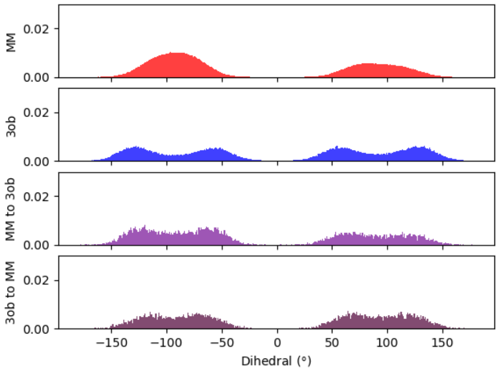

(a) χ_1

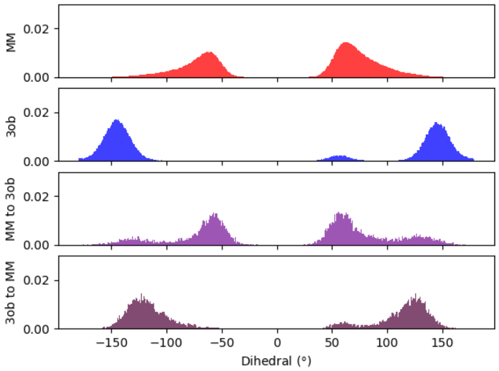

(b) χ_2

Figure 40: Dihedral distributions of molecule **20**.

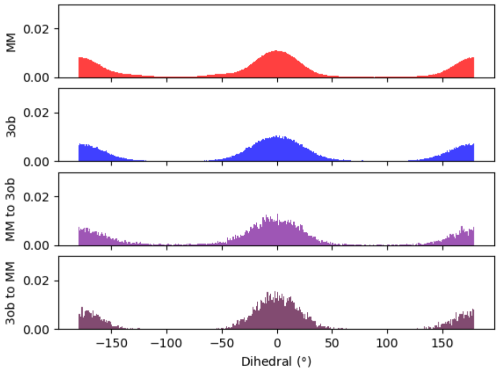

(a) χ_1

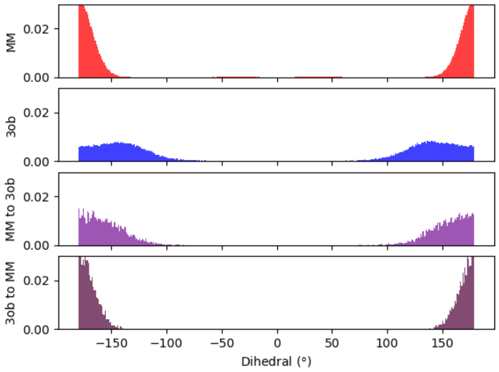

(b) χ_2

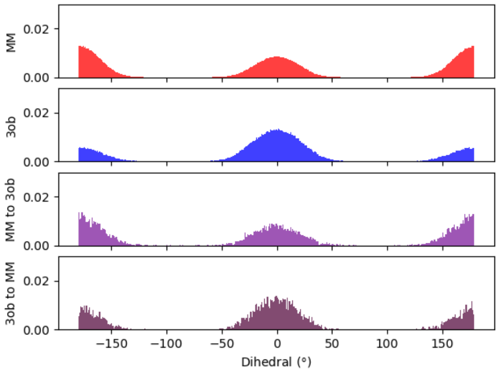

(c) χ_3

Figure 41: Dihedral distributions of molecule **21**.

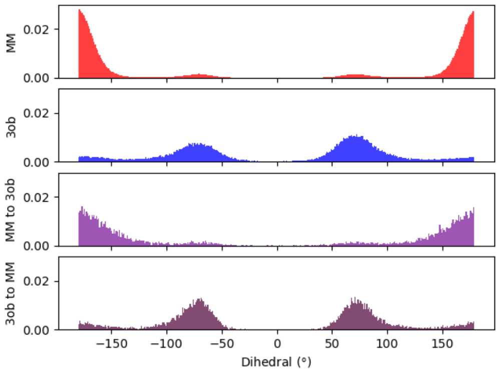

(a) χ_1

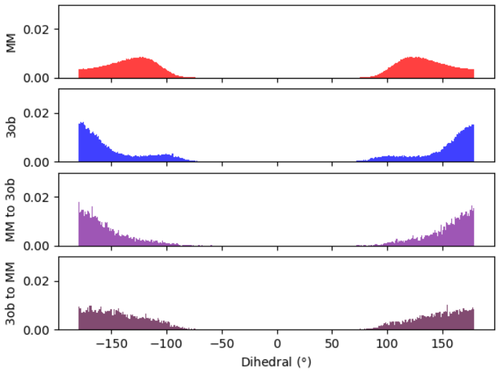

(b) χ_2

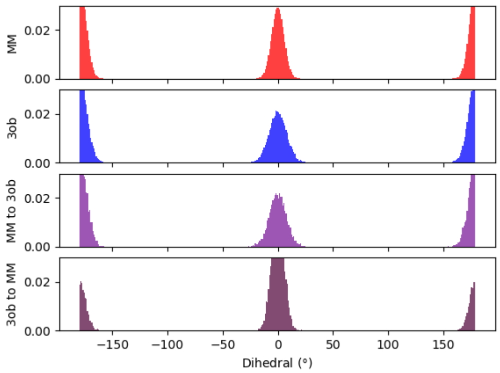

(c) χ_3

Figure 42: Dihedral distributions of molecule **22**.
