# Supplementary figures and images for: The Good, the Bad, and the Ugly: “HiPen”, a New Dataset for Validating (S)QM/MM Free Energy Simulations (part 1 of 2)
Source: Molecules. 2019 Feb 14;24(4):681. doi: 10.3390/molecules24040681 (PMC6413162; doi:10.3390/molecules24040681)

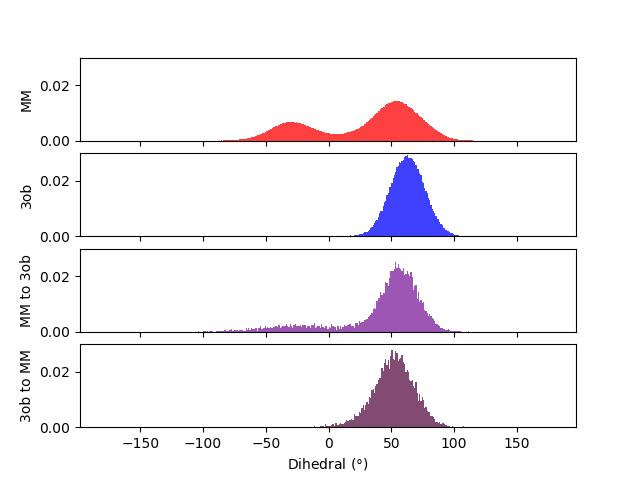

Supplement: Supplementary file 1 [file molecules-24-00681-s001.zip › final-SI/images/zinc_00138607-mm-3ob-subplots-dihe-chi3.png]

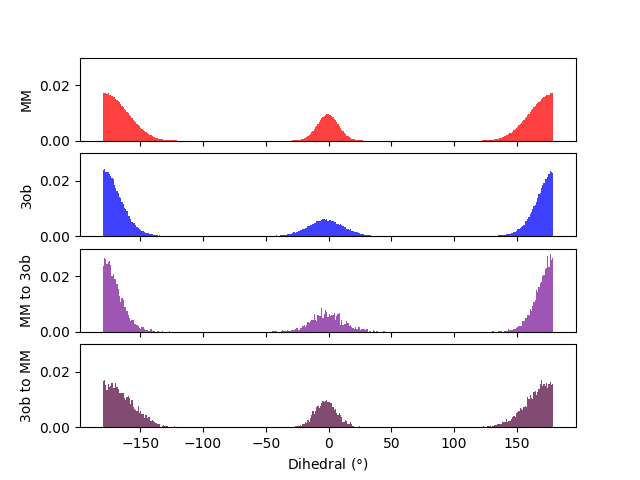

Supplement: Supplementary file 1 [file molecules-24-00681-s001.zip › final-SI/images/zinc_00095858-mm-3ob-subplots-dihe-chi4.png]

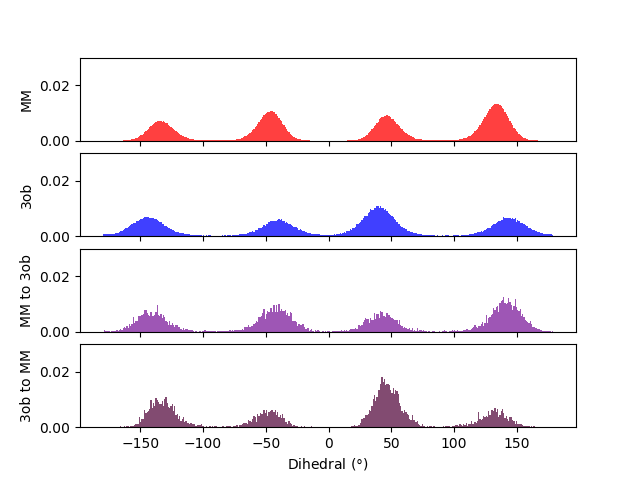

Supplement: Supplementary file 1 [file molecules-24-00681-s001.zip › final-SI/images/zinc_00167648-mm-3ob-subplots-dihe-chi1.png]

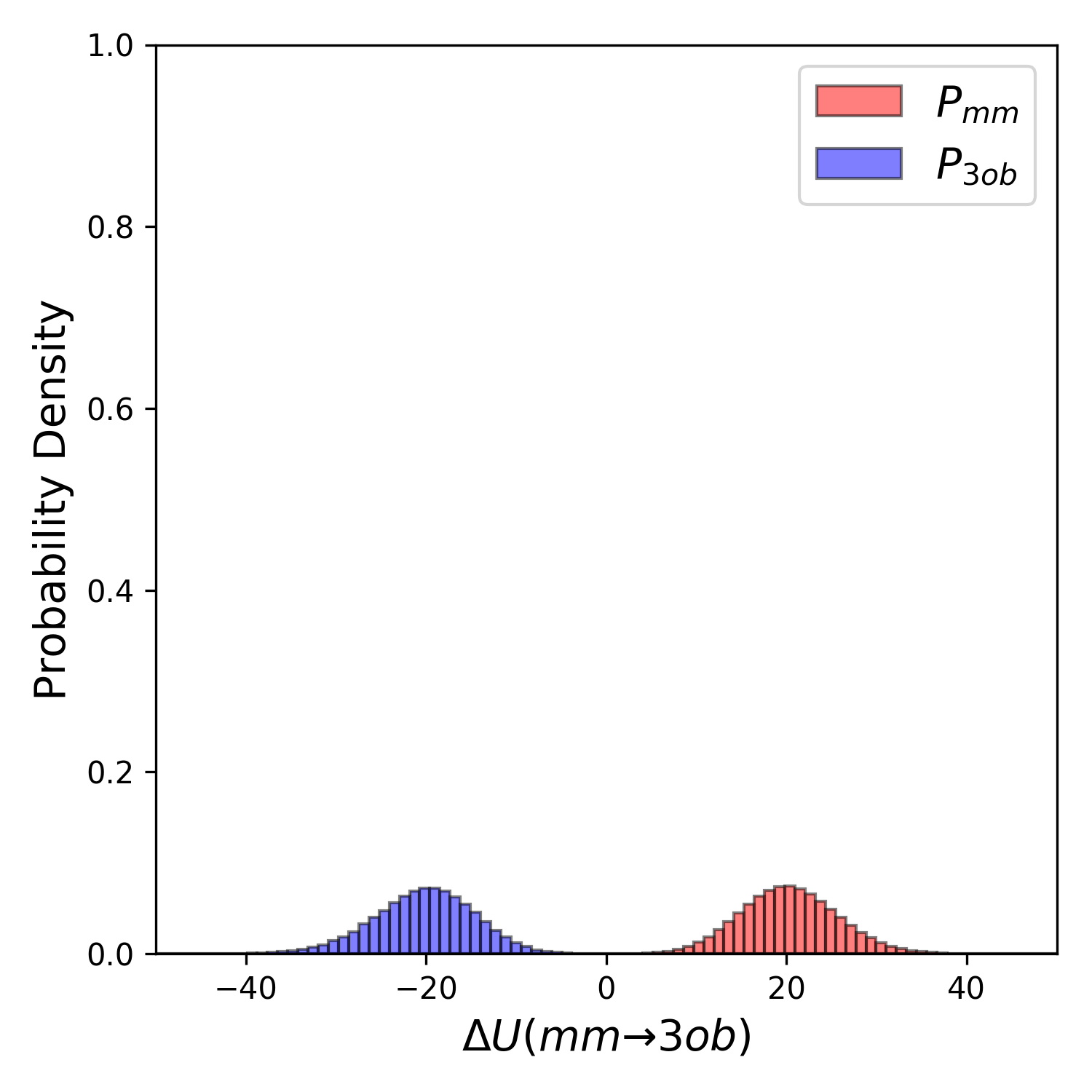

Supplement: Supplementary file 1 [file molecules-24-00681-s001.zip › final-SI/images/zinc_00164361-mm-3ob-fep-overlap.png]

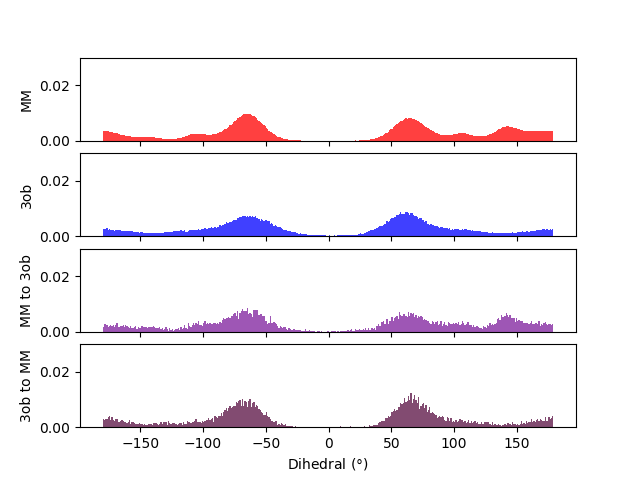

Supplement: Supplementary file 1 [file molecules-24-00681-s001.zip › final-SI/images/zinc_03127671-mm-3ob-subplots-dihe-chi5.png]

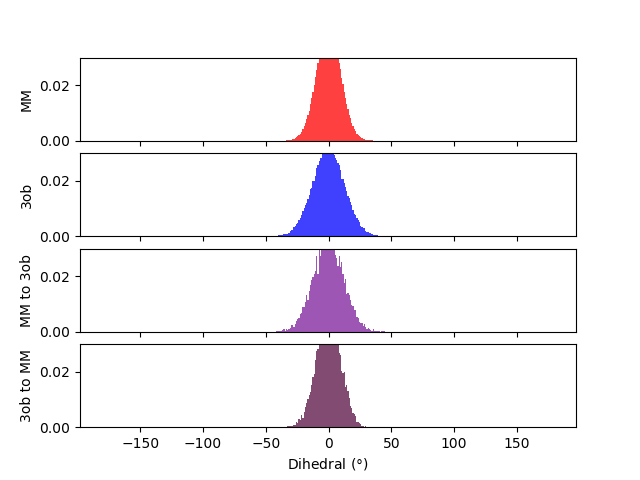

Supplement: Supplementary file 1 [file molecules-24-00681-s001.zip › final-SI/images/zinc_00107550-mm-3ob-subplots-dihe-chi3.png]

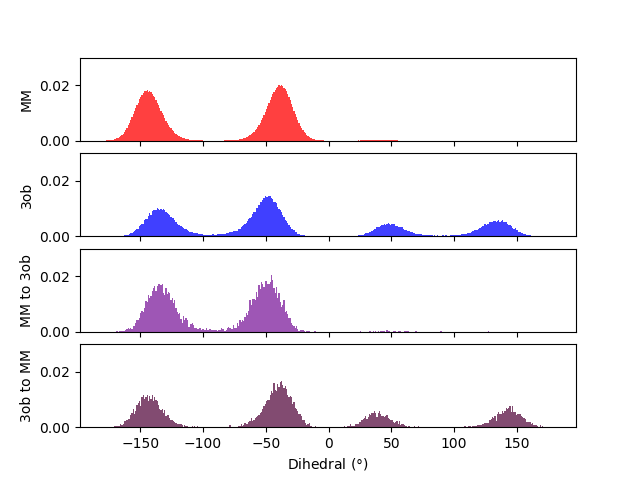

Supplement: Supplementary file 1 [file molecules-24-00681-s001.zip › final-SI/images/zinc_01867000-mm-3ob-subplots-dihe-chi2.png]

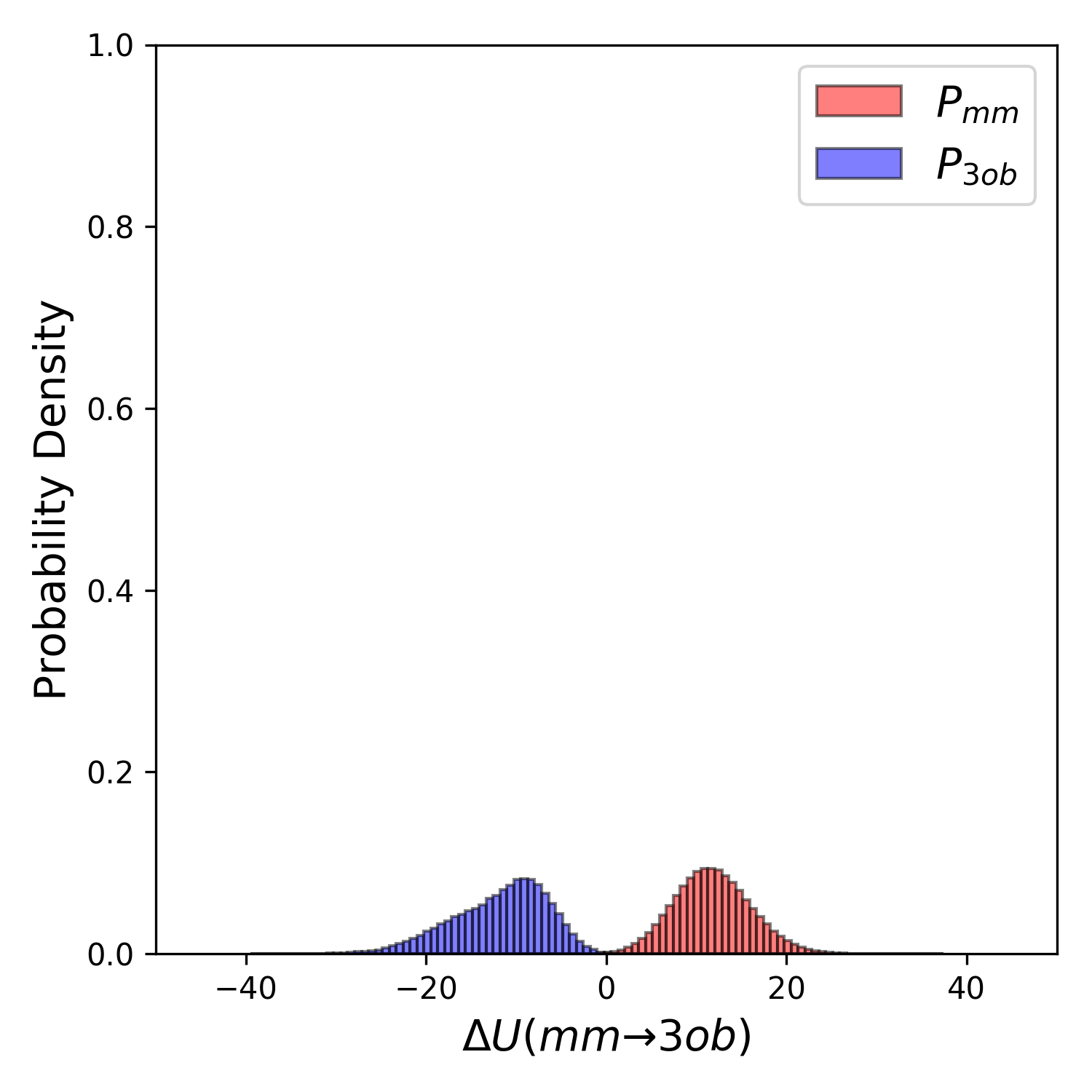

Supplement: Supplementary file 1 [file molecules-24-00681-s001.zip › final-SI/images/zinc_00077329-mm-3ob-fep-overlap.png]

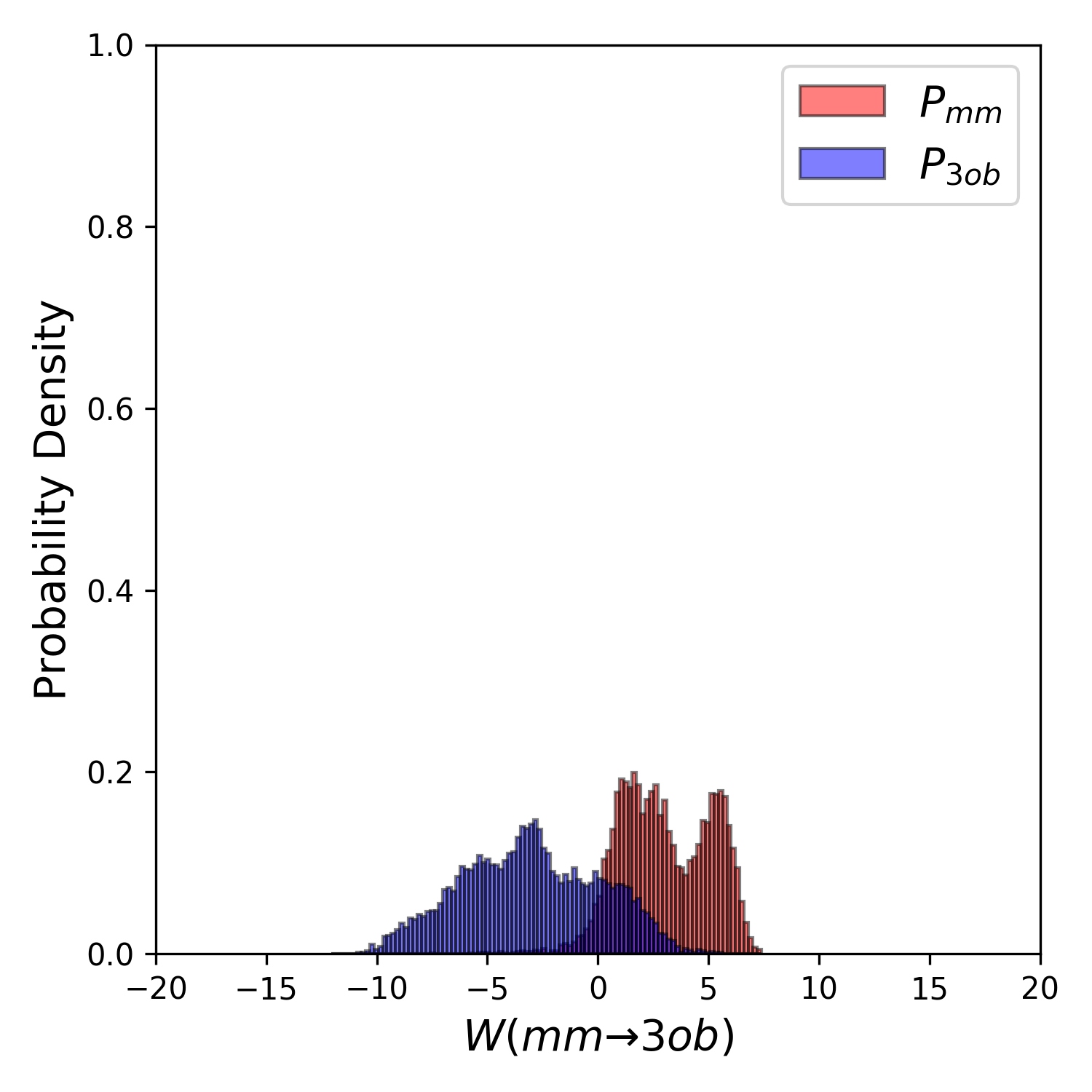

Supplement: Supplementary file 1 [file molecules-24-00681-s001.zip › final-SI/images/zinc_00095858-mm-3ob-jar-overlap.png]

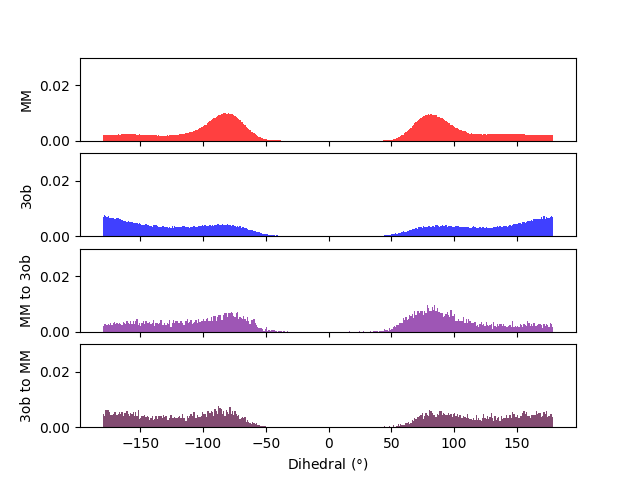

Supplement: Supplementary file 1 [file molecules-24-00681-s001.zip › final-SI/images/zinc_04344392-mm-3ob-subplots-dihe-chi7.png]

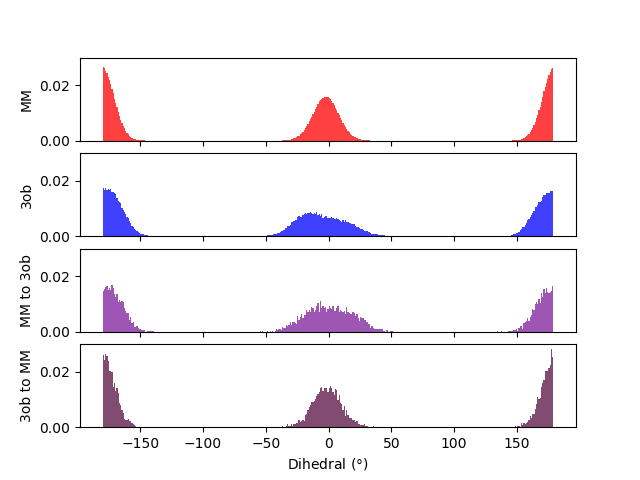

Supplement: Supplementary file 1 [file molecules-24-00681-s001.zip › final-SI/images/zinc_00087557-mm-3ob-subplots-dihe-chi4.png]

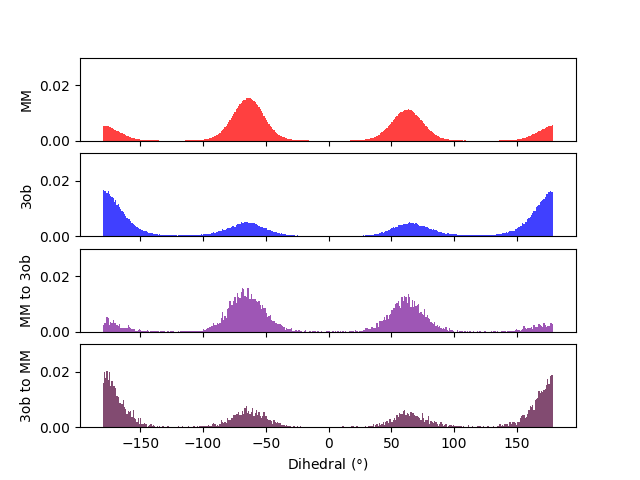

Supplement: Supplementary file 1 [file molecules-24-00681-s001.zip › final-SI/images/zinc_04344392-mm-3ob-subplots-dihe-chi9.png]

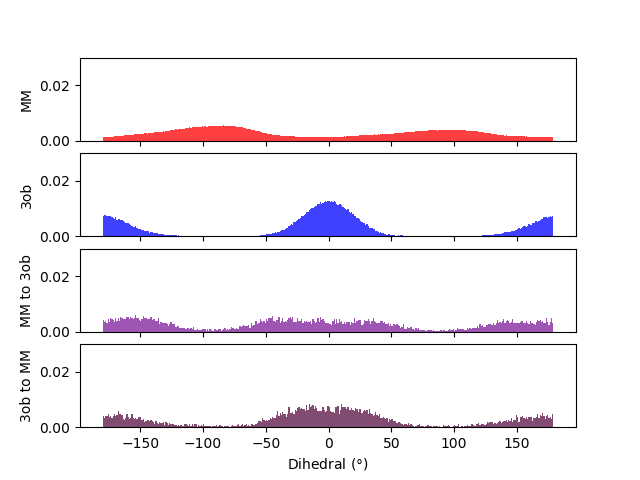

Supplement: Supplementary file 1 [file molecules-24-00681-s001.zip › final-SI/images/zinc_03127671-mm-3ob-subplots-dihe-chi1.png]

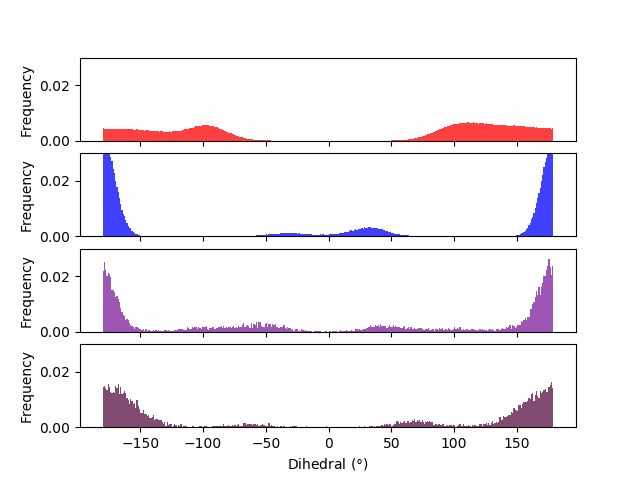

Supplement: Supplementary file 1 [file molecules-24-00681-s001.zip › final-SI/images/zinc_00123162-mm-3ob-subplots-dihe-chi5.png]

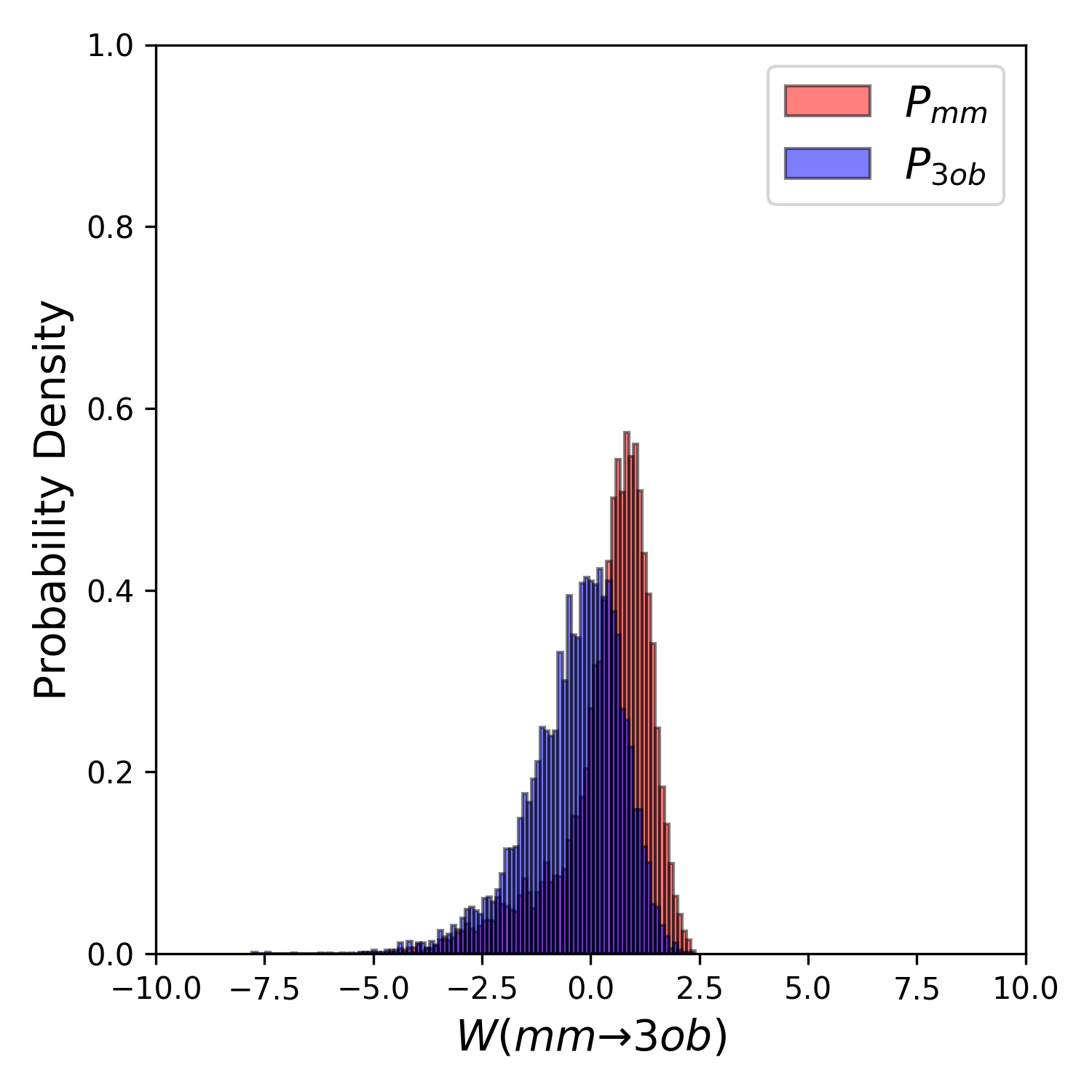

Supplement: Supplementary file 1 [file molecules-24-00681-s001.zip › final-SI/images/zinc_06568023-mm-3ob-jar-overlap.png]

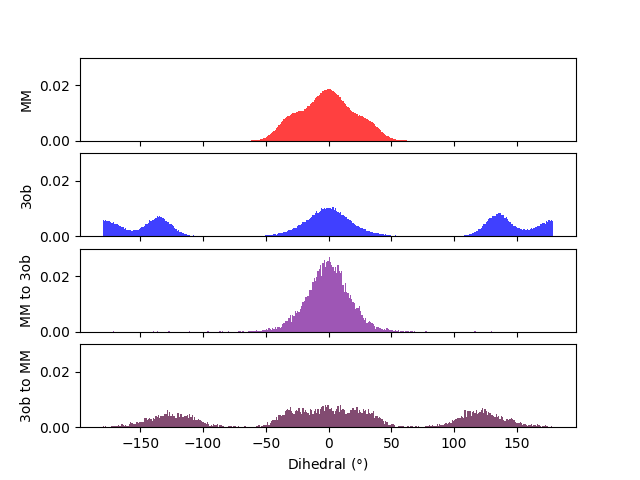

Supplement: Supplementary file 1 [file molecules-24-00681-s001.zip › final-SI/images/zinc_00107778-mm-3ob-subplots-dihe-chi2.png]

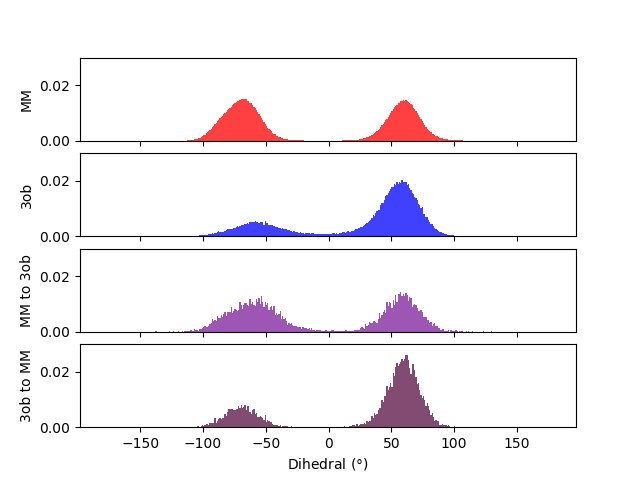

Supplement: Supplementary file 1 [file molecules-24-00681-s001.zip › final-SI/images/zinc_00138607-mm-3ob-subplots-dihe-chi2.png]

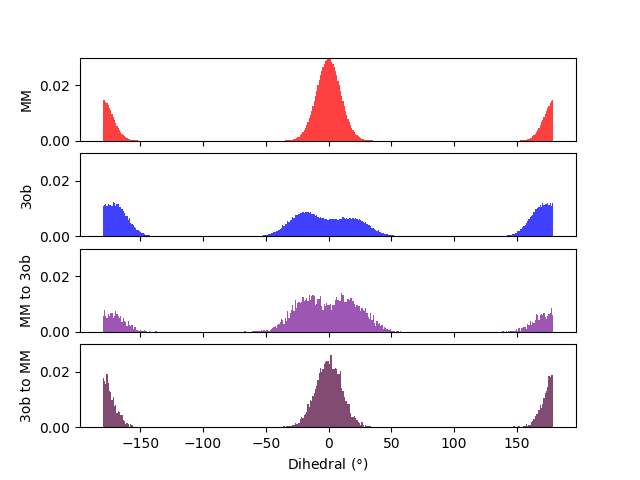

Supplement: Supplementary file 1 [file molecules-24-00681-s001.zip › final-SI/images/zinc_01755198-mm-3ob-subplots-dihe-chi3.png]

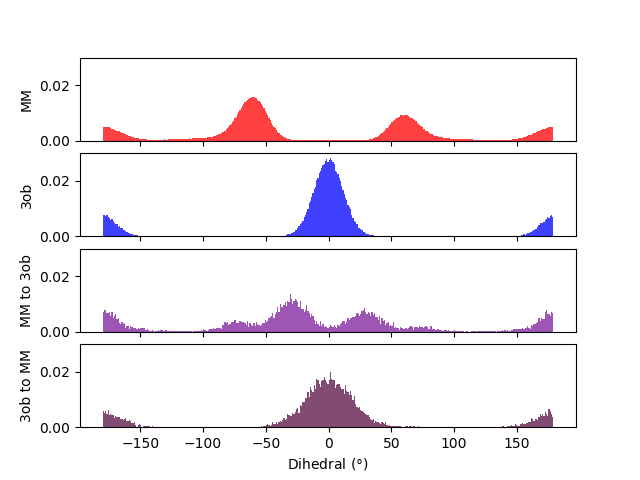

Supplement: Supplementary file 1 [file molecules-24-00681-s001.zip › final-SI/images/zinc_00061095-mm-3ob-subplots-dihe-chi4.png]

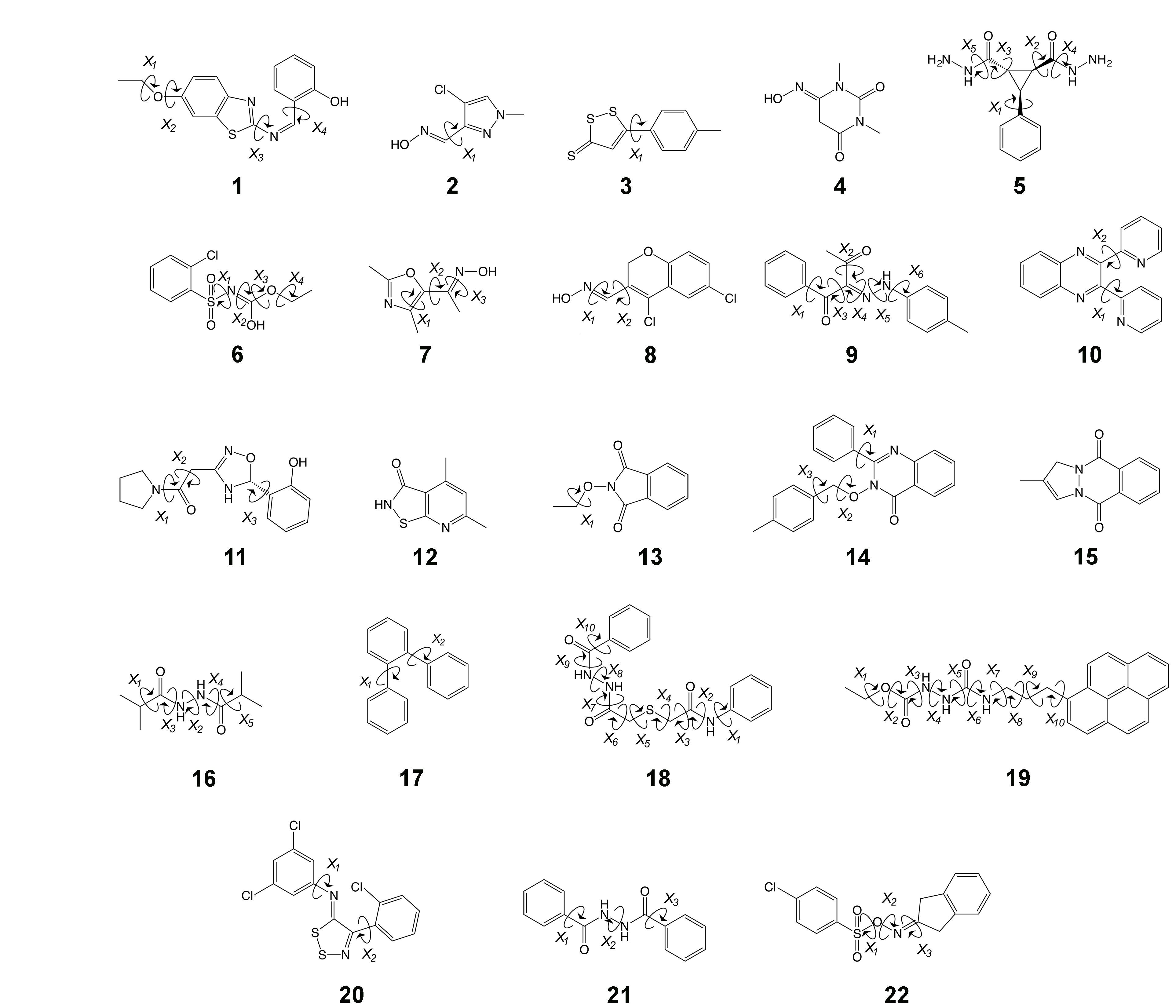

Supplement: Supplementary file 1 [file molecules-24-00681-s001.zip › final-SI/images/hipen22-chemdraw-11x14-300dpi.png]

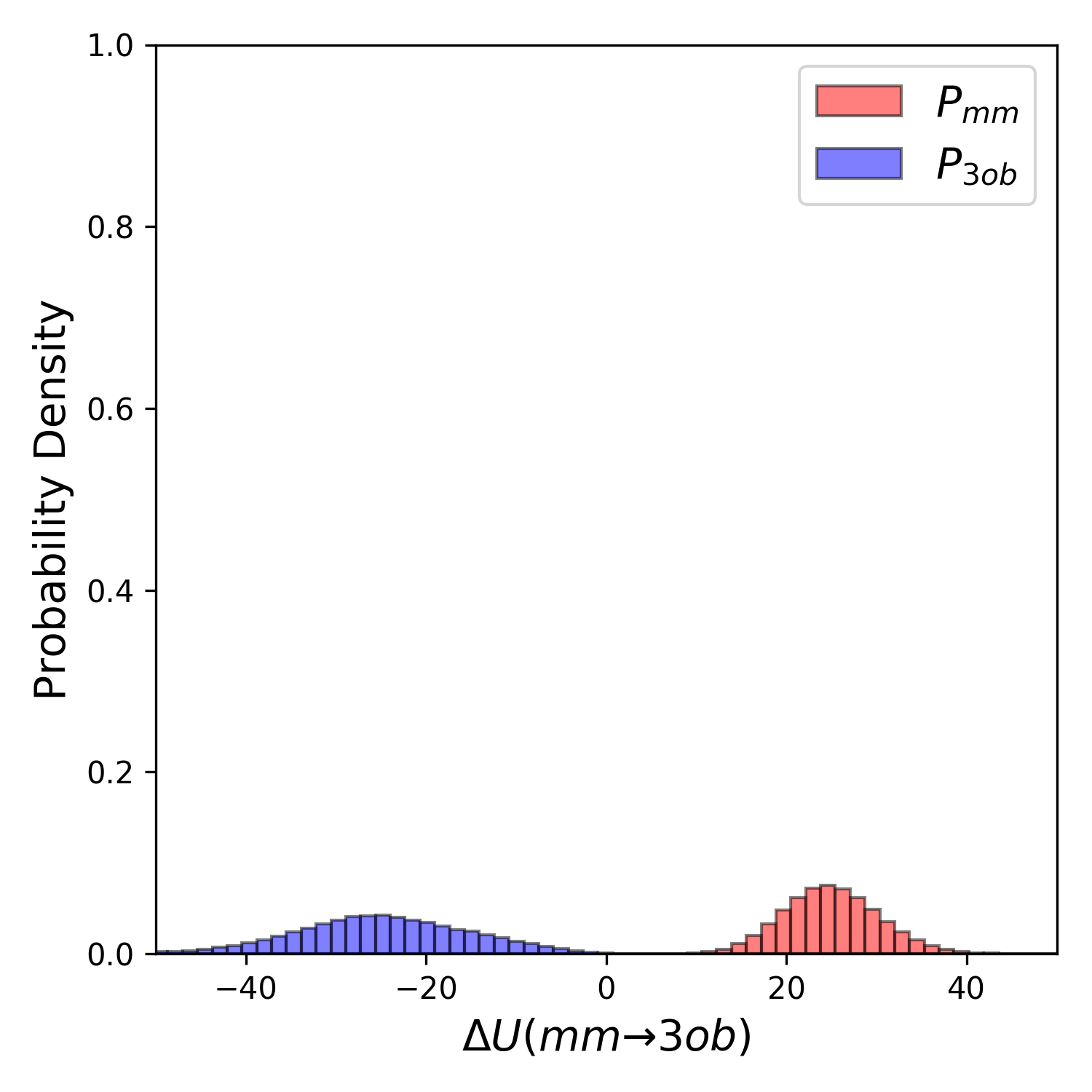

Supplement: Supplementary file 1 [file molecules-24-00681-s001.zip › final-SI/images/zinc_03127671-mm-3ob-fep-overlap.png]

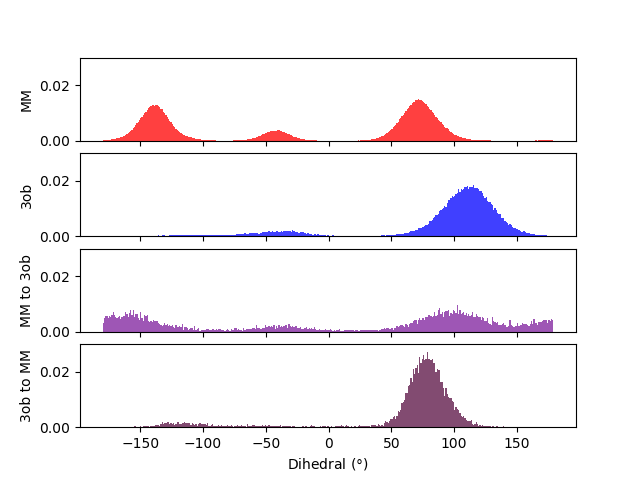

Supplement: Supplementary file 1 [file molecules-24-00681-s001.zip › final-SI/images/zinc_00087557-mm-3ob-subplots-dihe-chi2.png]

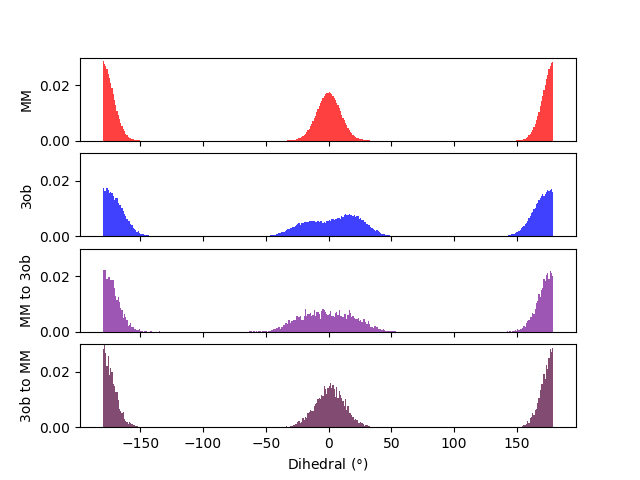

Supplement: Supplementary file 1 [file molecules-24-00681-s001.zip › final-SI/images/zinc_03127671-mm-3ob-subplots-dihe-chi7.png]

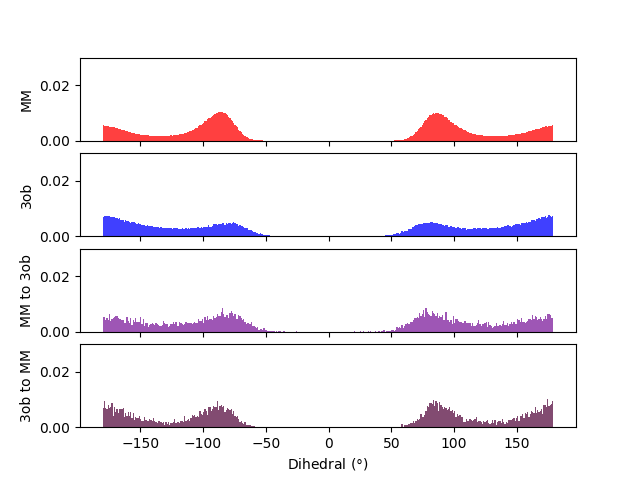

Supplement: Supplementary file 1 [file molecules-24-00681-s001.zip › final-SI/images/zinc_04344392-mm-3ob-subplots-dihe-chi1.png]

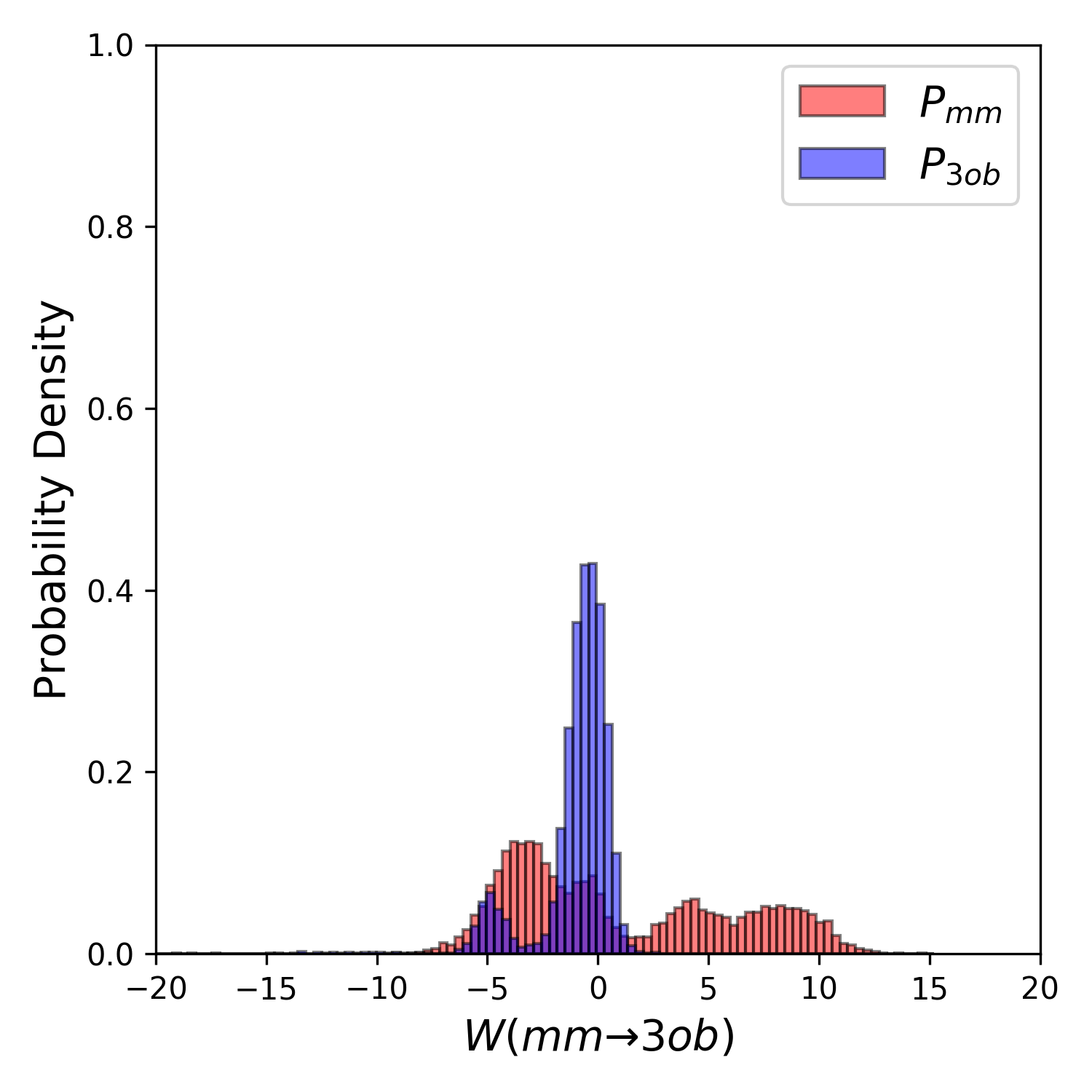

Supplement: Supplementary file 1 [file molecules-24-00681-s001.zip › final-SI/images/zinc_00061095-mm-3ob-jar-overlap.png]

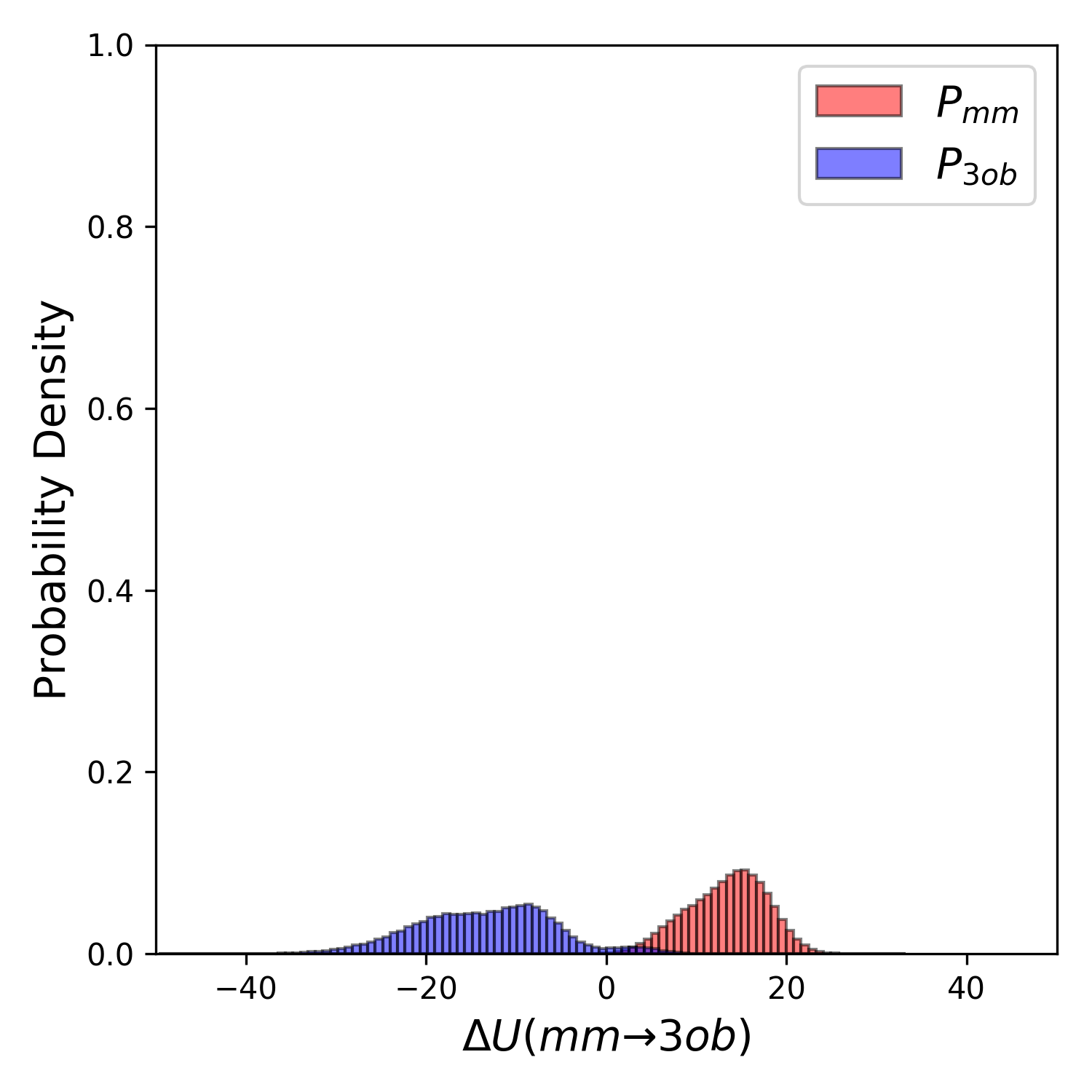

Supplement: Supplementary file 1 [file molecules-24-00681-s001.zip › final-SI/images/zinc_00107778-mm-3ob-fep-overlap.png]

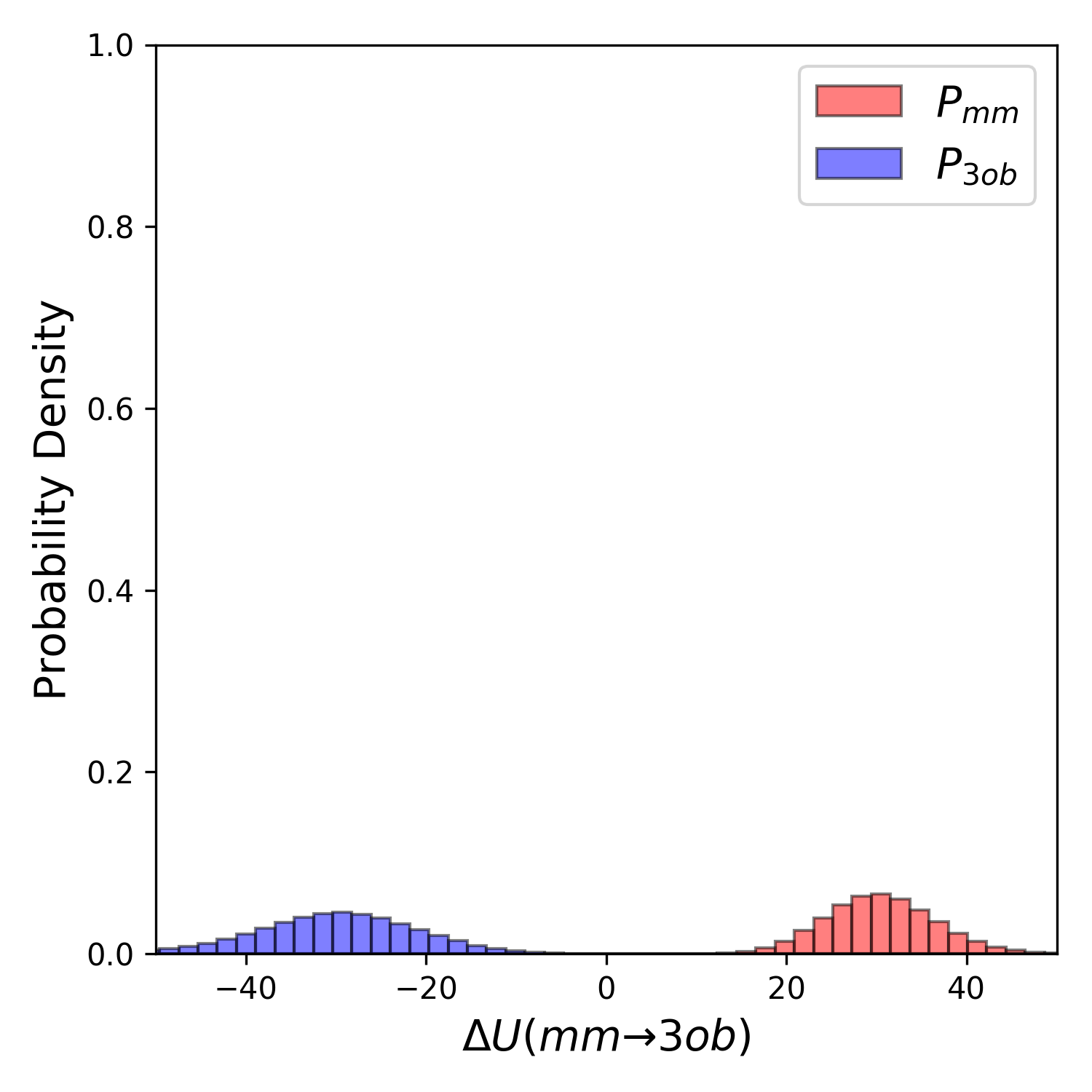

Supplement: Supplementary file 1 [file molecules-24-00681-s001.zip › final-SI/images/zinc_04344392-mm-3ob-fep-overlap.png]

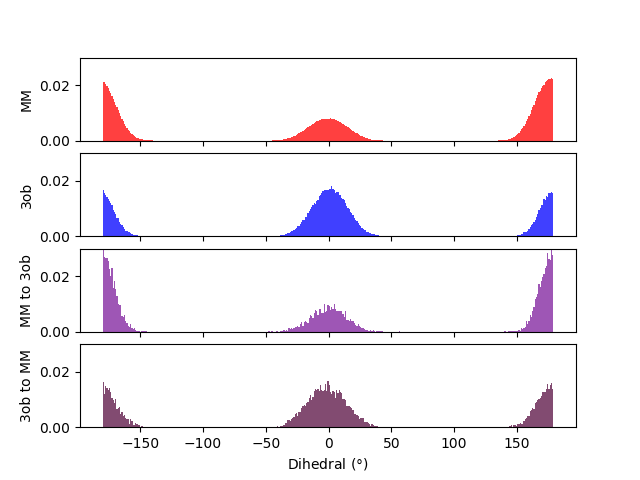

Supplement: Supplementary file 1 [file molecules-24-00681-s001.zip › final-SI/images/zinc_00138607-mm-3ob-subplots-dihe-chi1.png]

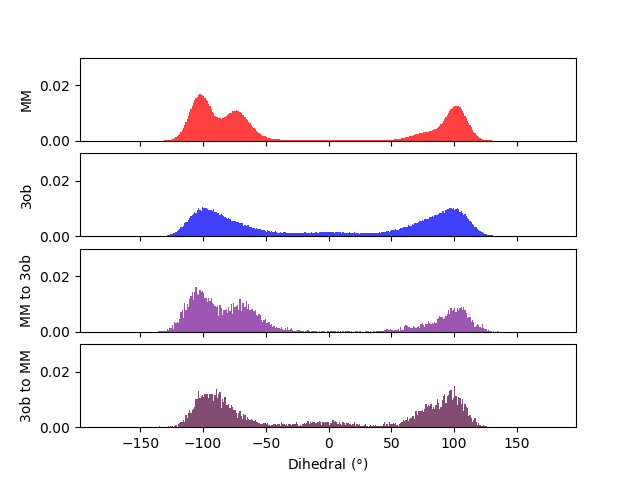

Supplement: Supplementary file 1 [file molecules-24-00681-s001.zip › final-SI/images/zinc_04344392-mm-3ob-subplots-dihe-chi10.png]

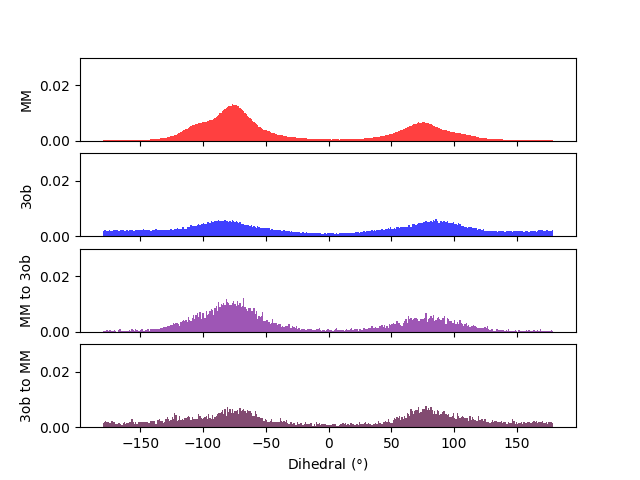

Supplement: Supplementary file 1 [file molecules-24-00681-s001.zip › final-SI/images/zinc_03127671-mm-3ob-subplots-dihe-chi6.png]

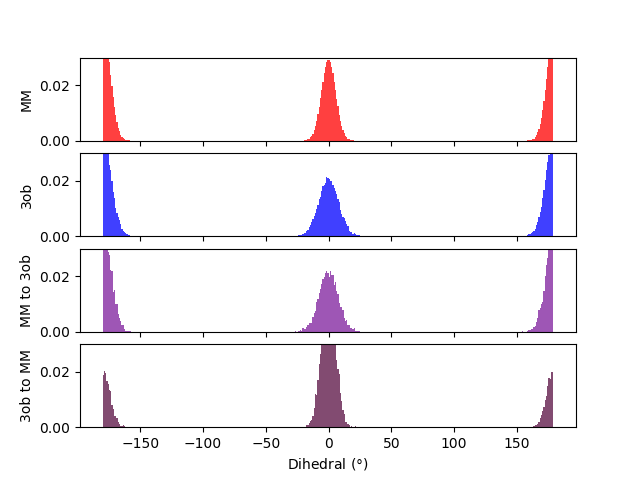

Supplement: Supplementary file 1 [file molecules-24-00681-s001.zip › final-SI/images/zinc_33381936-mm-3ob-subplots-dihe-chi3.png]

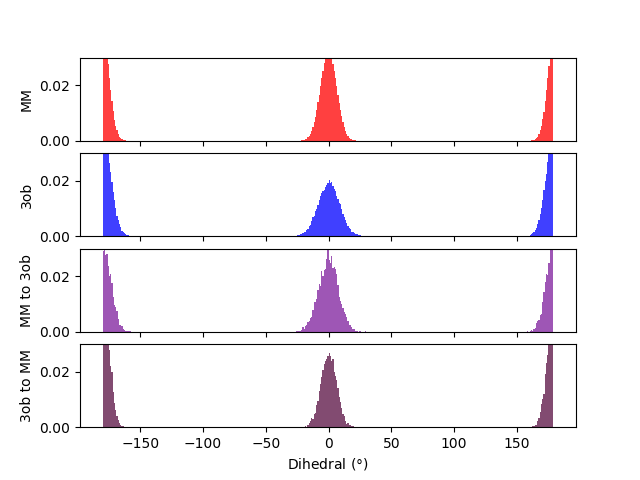

Supplement: Supplementary file 1 [file molecules-24-00681-s001.zip › final-SI/images/zinc_00107550-mm-3ob-subplots-dihe-chi1.png]

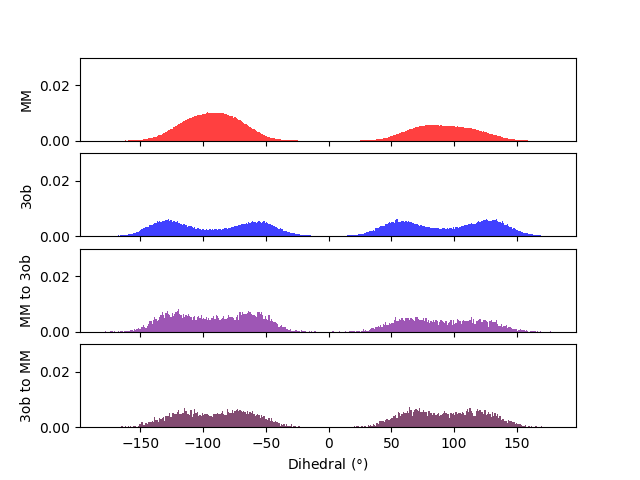

Supplement: Supplementary file 1 [file molecules-24-00681-s001.zip › final-SI/images/zinc_04363792-mm-3ob-subplots-dihe-chi1.png]

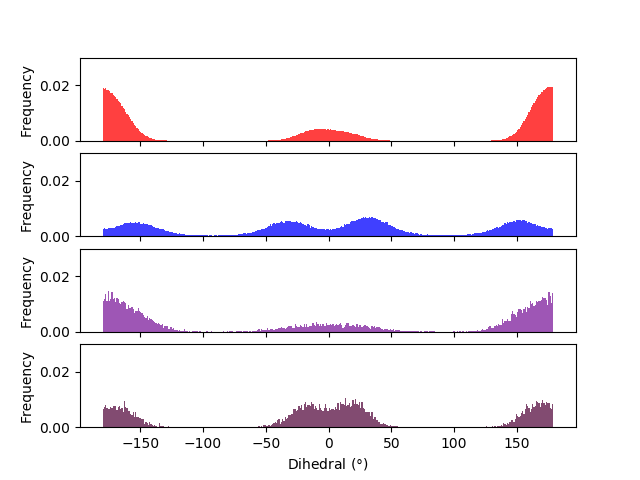

Supplement: Supplementary file 1 [file molecules-24-00681-s001.zip › final-SI/images/zinc_00123162-mm-3ob-subplots-dihe-chi1.png]

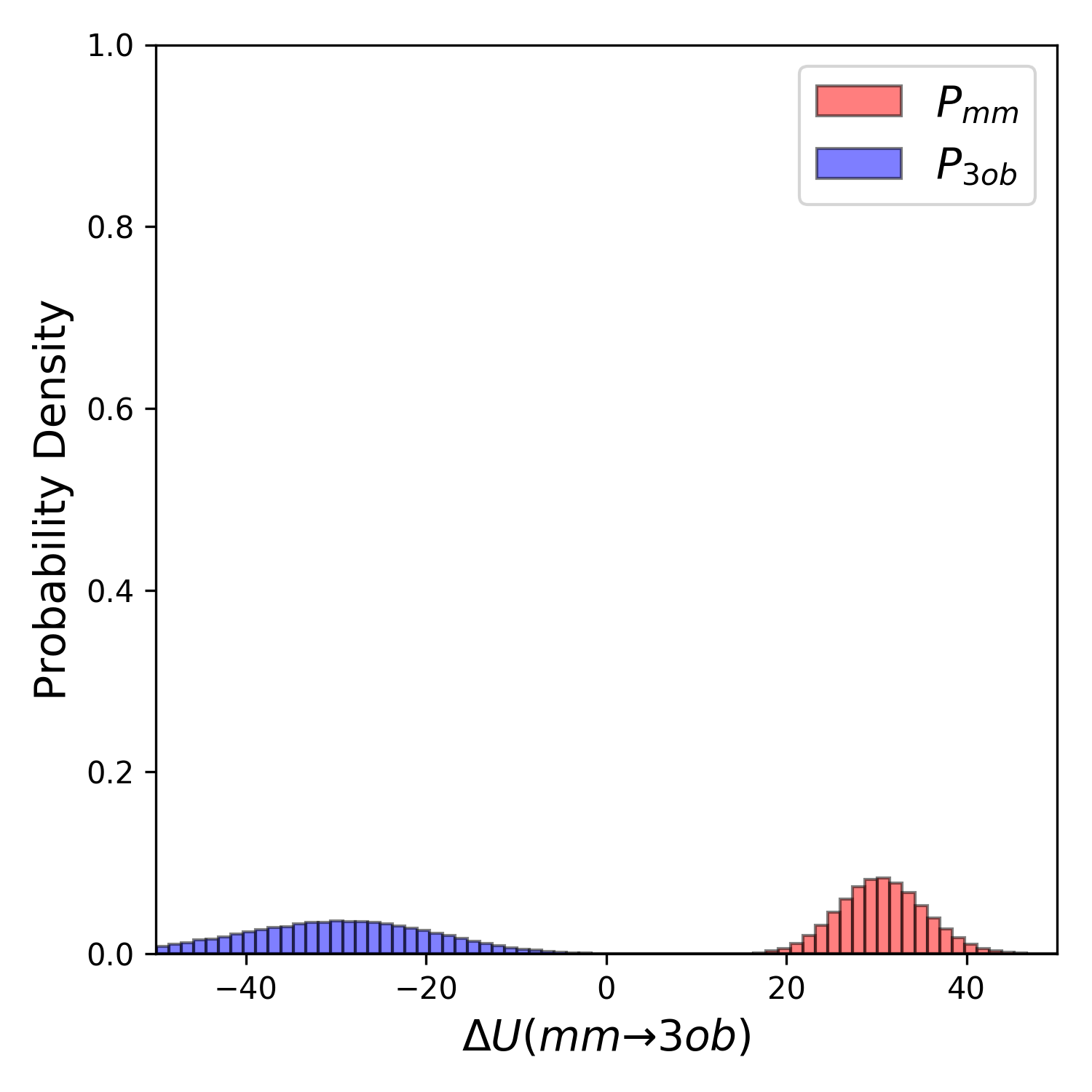

Supplement: Supplementary file 1 [file molecules-24-00681-s001.zip › final-SI/images/zinc_00140610-mm-3ob-fep-overlap.png]

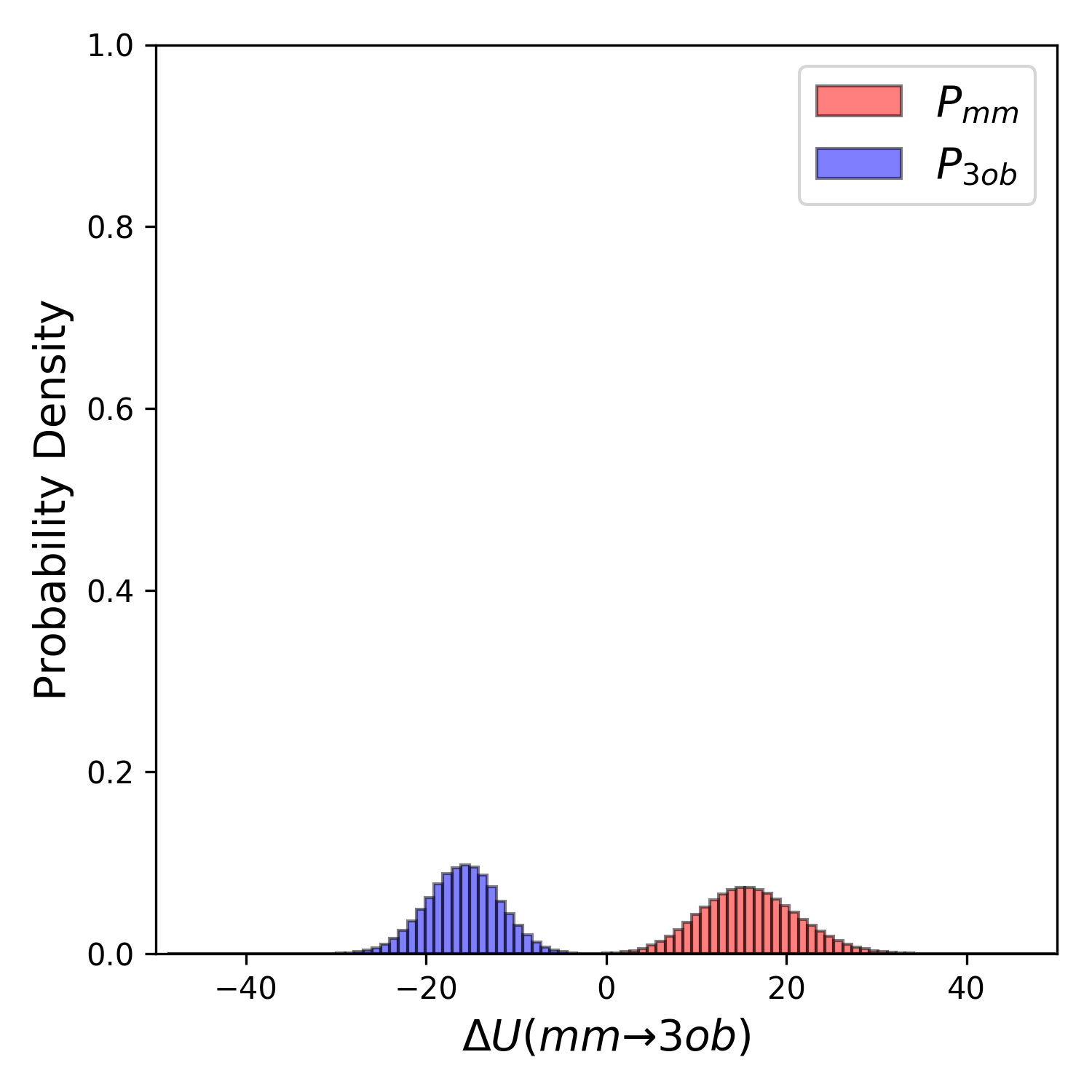

Supplement: Supplementary file 1 [file molecules-24-00681-s001.zip › final-SI/images/zinc_00133435-mm-3ob-fep-overlap.png]

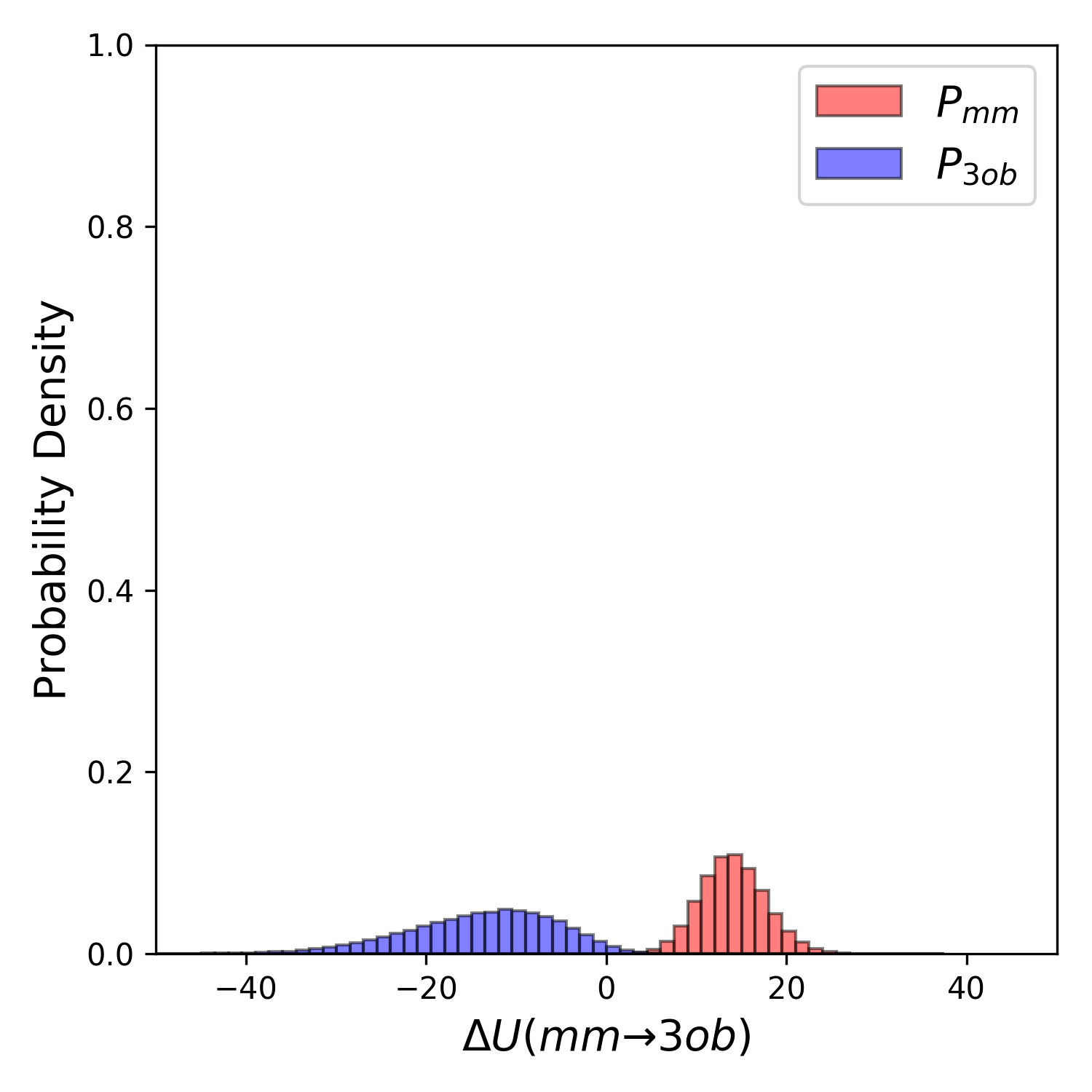

Supplement: Supplementary file 1 [file molecules-24-00681-s001.zip › final-SI/images/zinc_06568023-mm-3ob-fep-overlap.png]

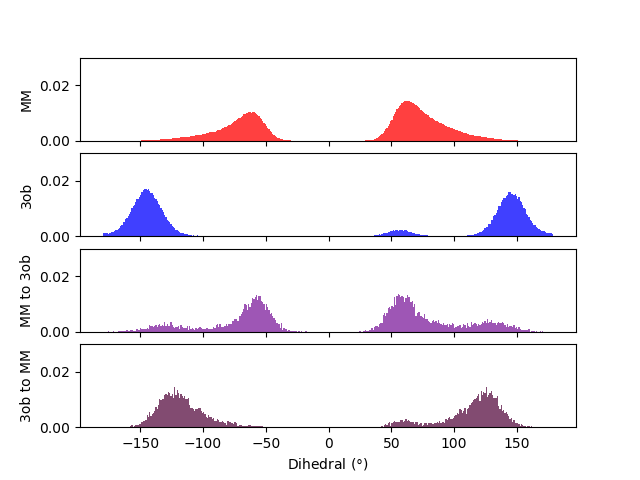

Supplement: Supplementary file 1 [file molecules-24-00681-s001.zip › final-SI/images/zinc_04363792-mm-3ob-subplots-dihe-chi2.png]

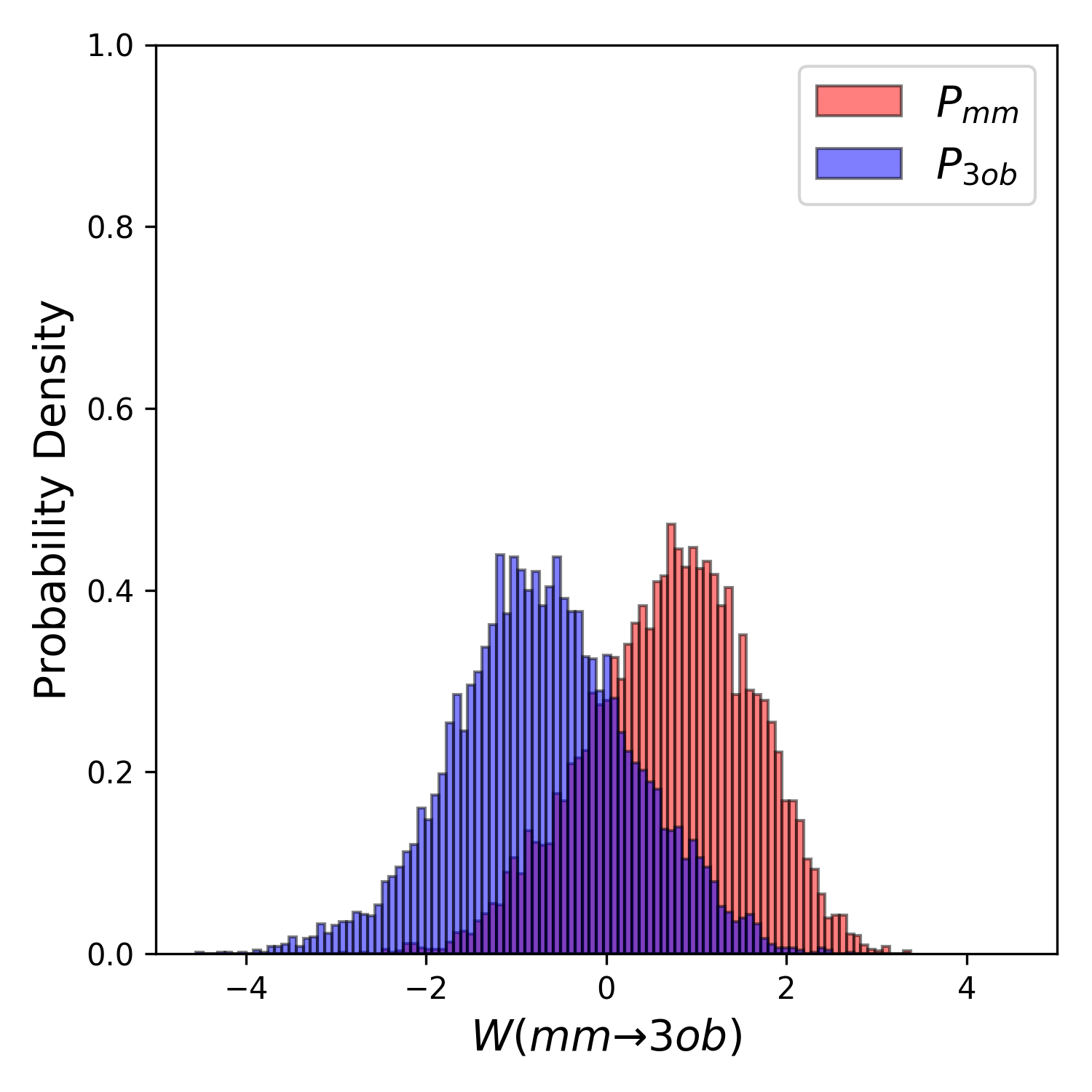

Supplement: Supplementary file 1 [file molecules-24-00681-s001.zip › final-SI/images/zinc_00167648-mm-3ob-jar-overlap.png]

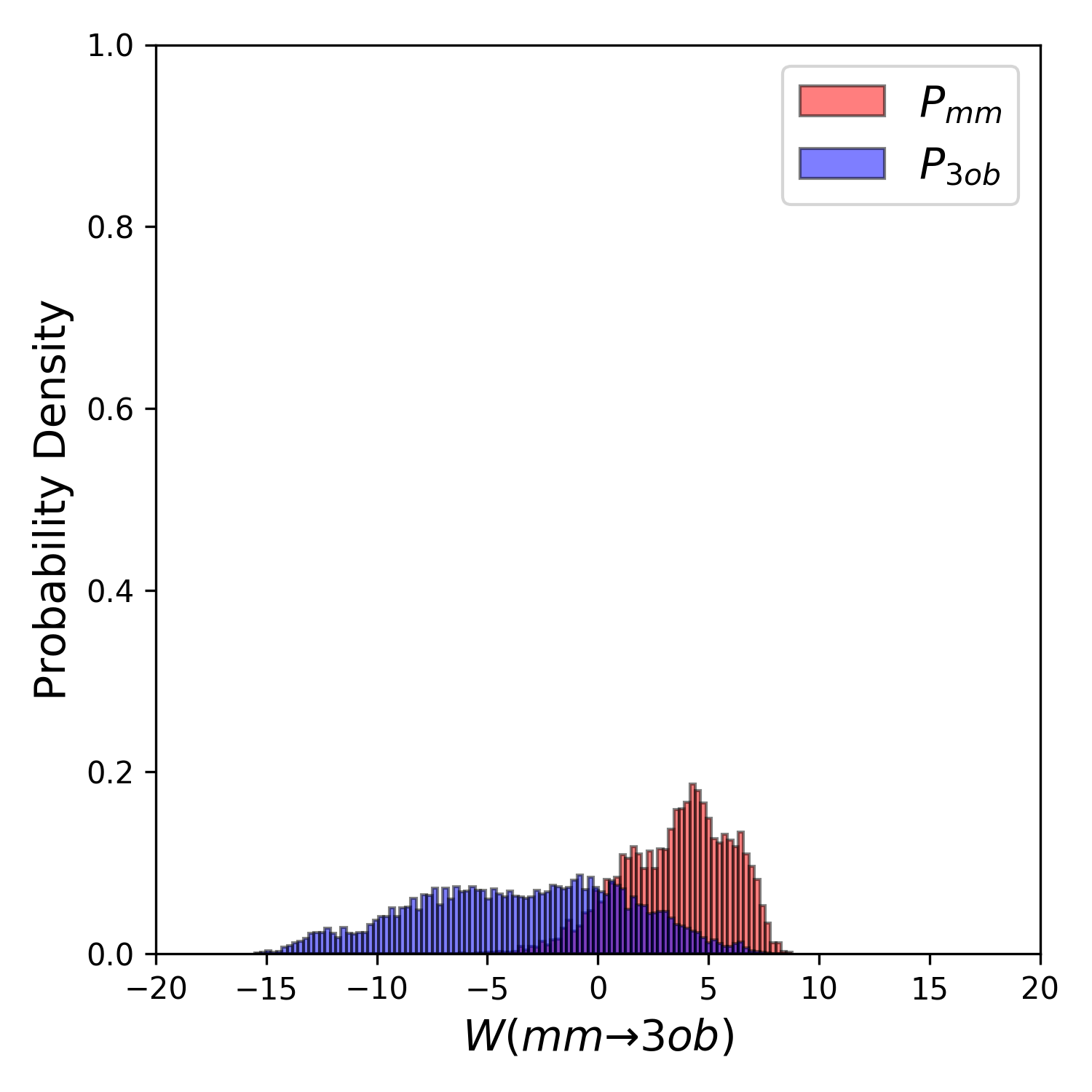

Supplement: Supplementary file 1 [file molecules-24-00681-s001.zip › final-SI/images/zinc_01755198-mm-3ob-jar-overlap.png]

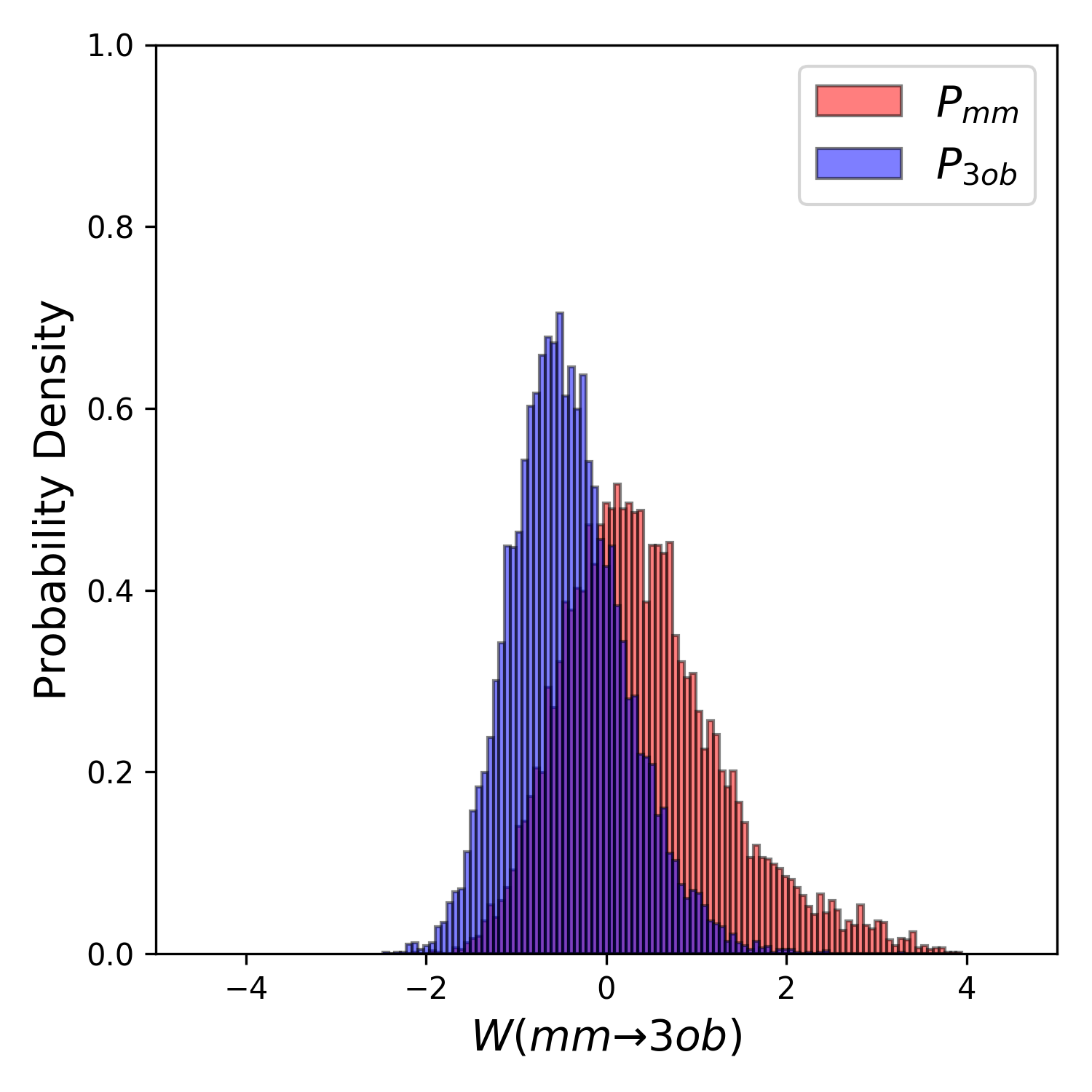

Supplement: Supplementary file 1 [file molecules-24-00681-s001.zip › final-SI/images/zinc_00079729-mm-3ob-jar-overlap.png]

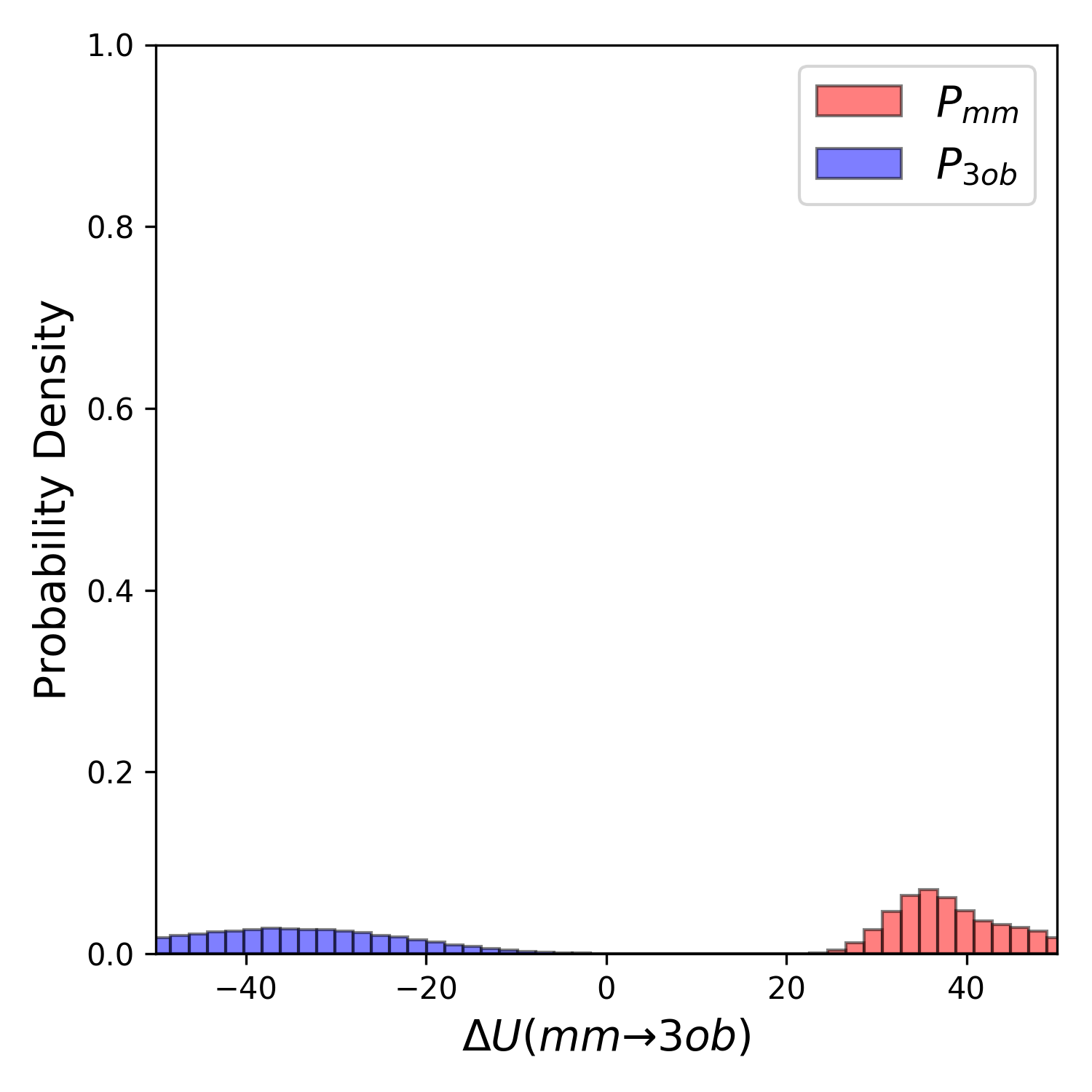

Supplement: Supplementary file 1 [file molecules-24-00681-s001.zip › final-SI/images/zinc_04363792-mm-3ob-fep-overlap.png]

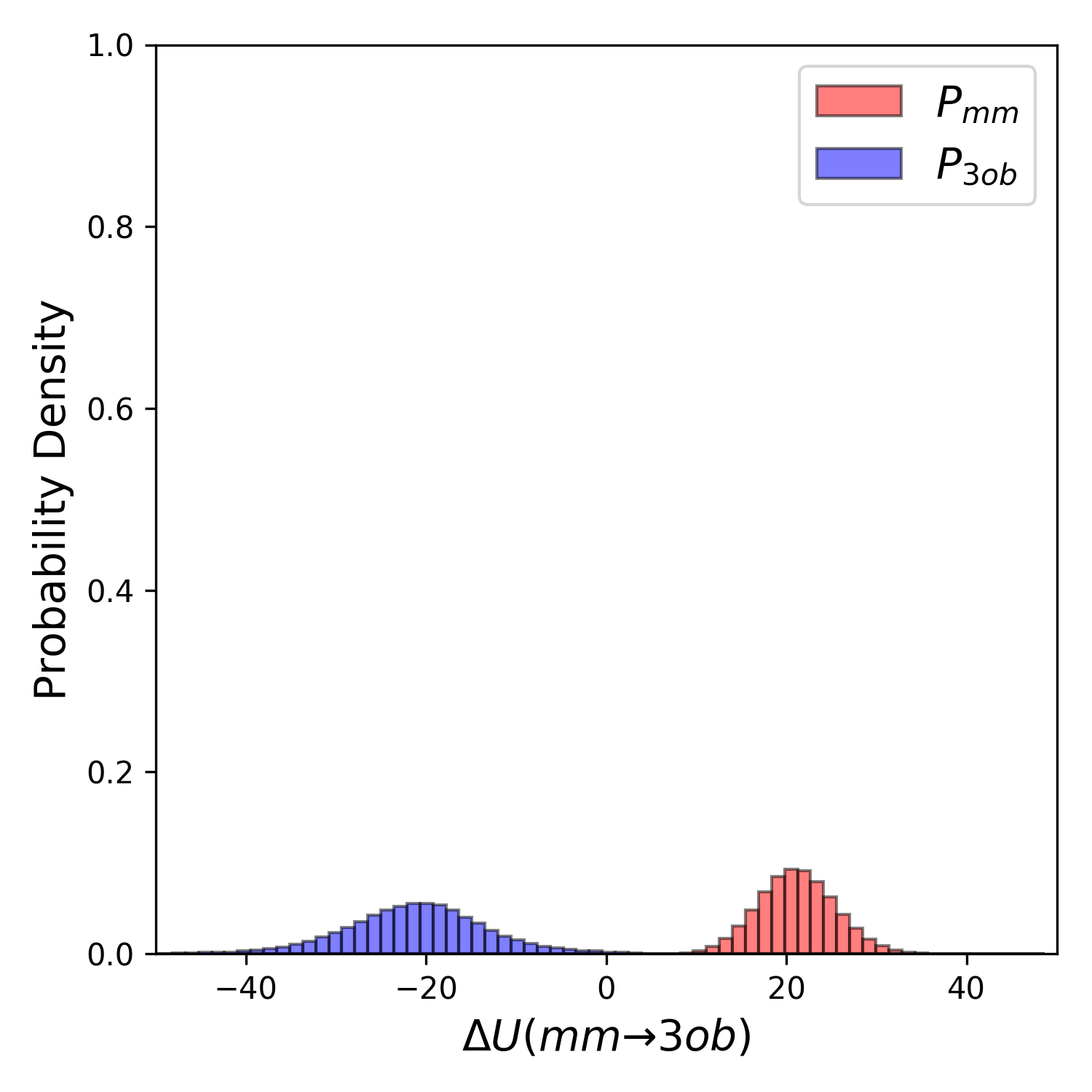

Supplement: Supplementary file 1 [file molecules-24-00681-s001.zip › final-SI/images/zinc_01755198-mm-3ob-fep-overlap.png]

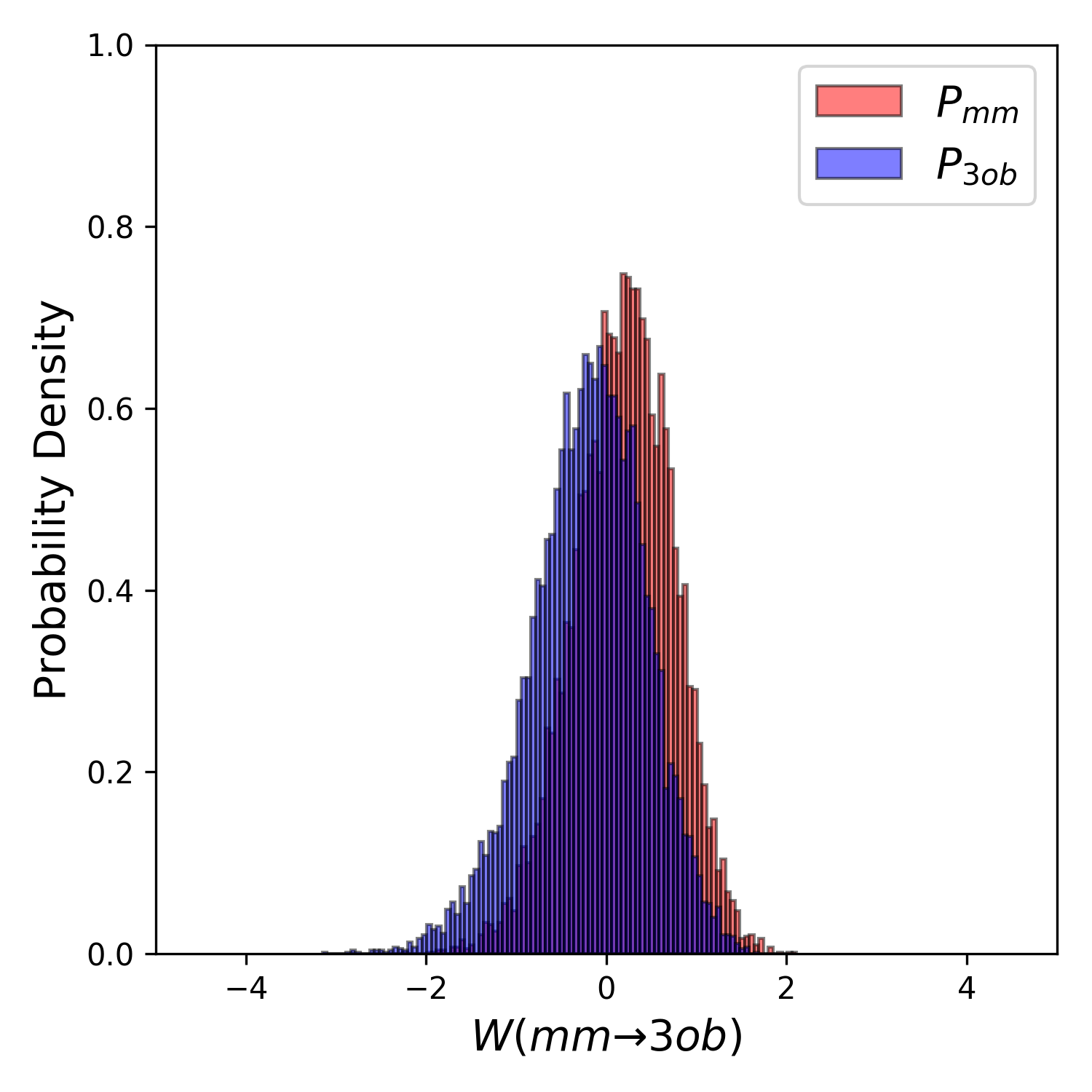

Supplement: Supplementary file 1 [file molecules-24-00681-s001.zip › final-SI/images/zinc_00140610-mm-3ob-jar-overlap.png]

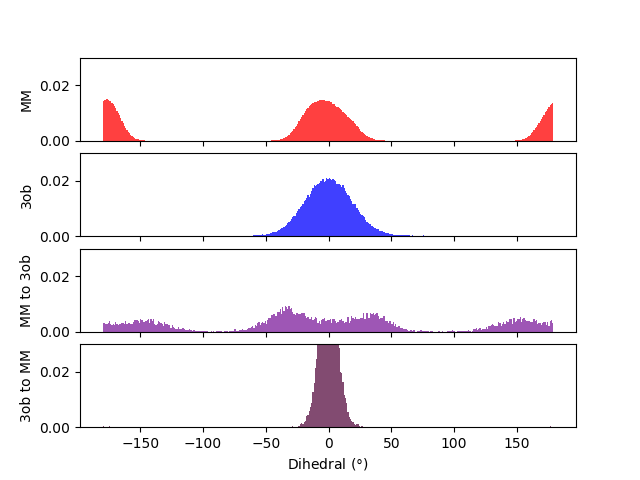

Supplement: Supplementary file 1 [file molecules-24-00681-s001.zip › final-SI/images/zinc_00061095-mm-3ob-subplots-dihe-chi3.png]

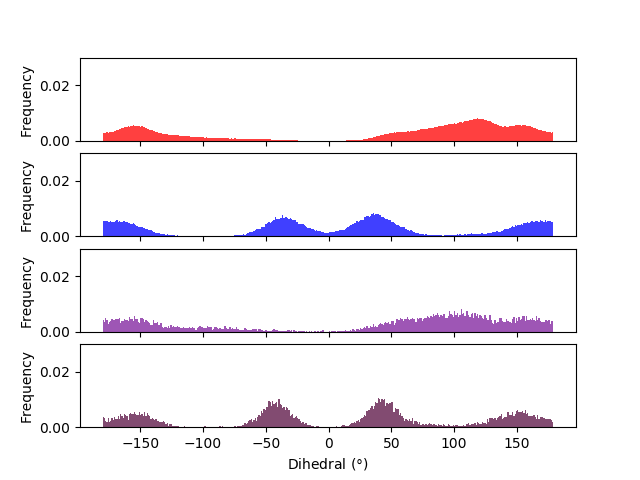

Supplement: Supplementary file 1 [file molecules-24-00681-s001.zip › final-SI/images/zinc_00123162-mm-3ob-subplots-dihe-chi3.png]

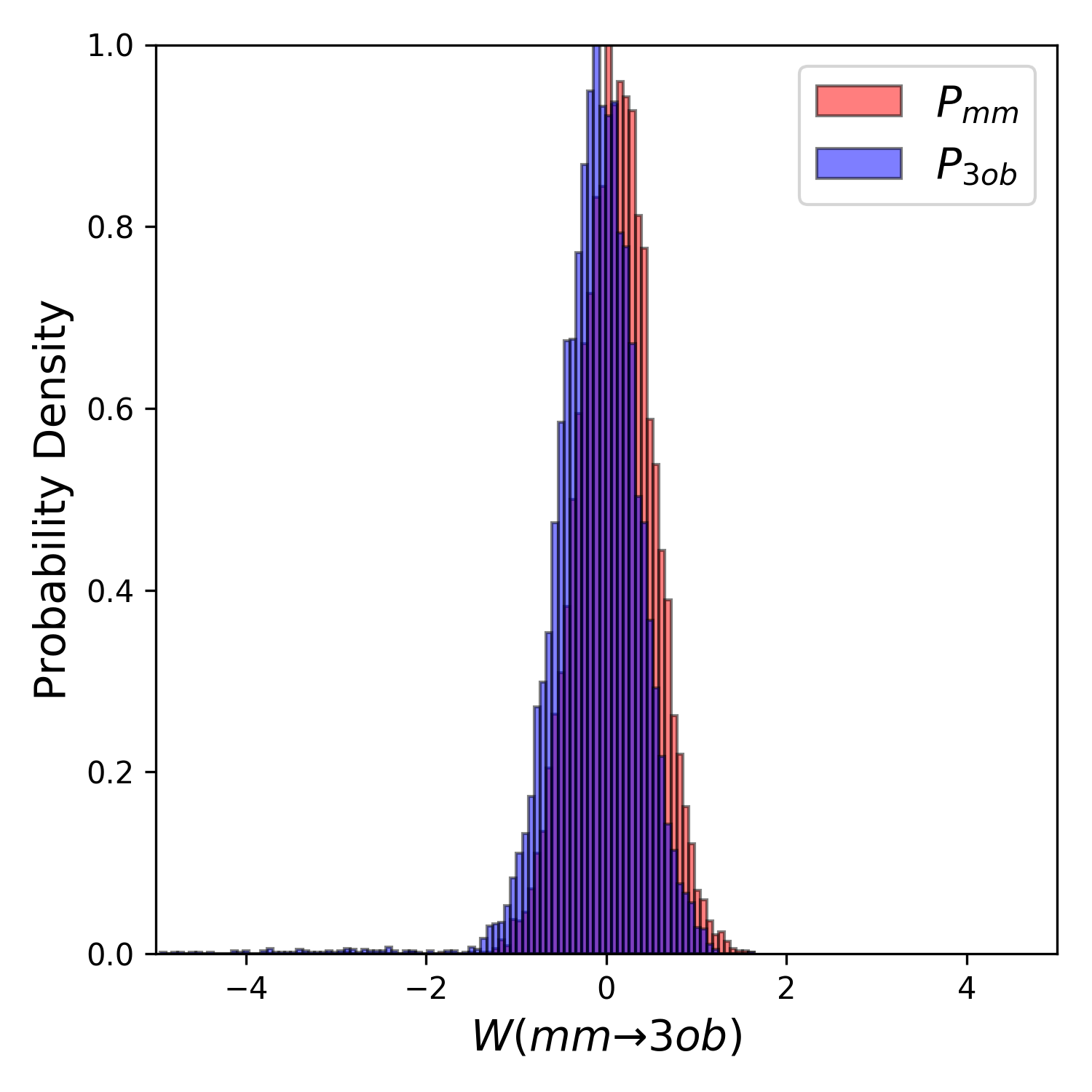

Supplement: Supplementary file 1 [file molecules-24-00681-s001.zip › final-SI/images/zinc_00086442-mm-3ob-jar-overlap.png]

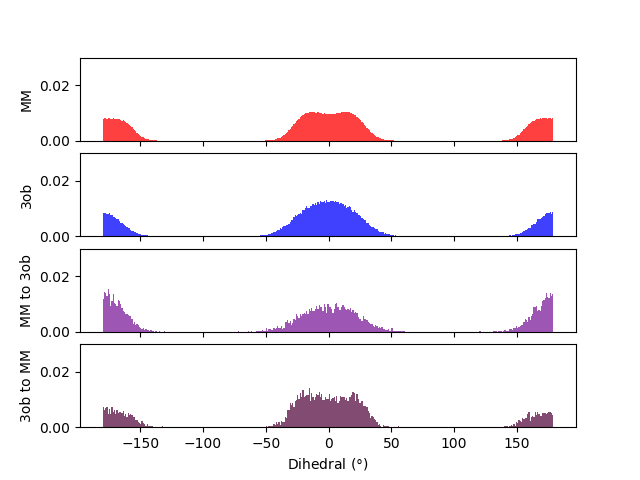

Supplement: Supplementary file 1 [file molecules-24-00681-s001.zip › final-SI/images/zinc_04344392-mm-3ob-subplots-dihe-chi6.png]

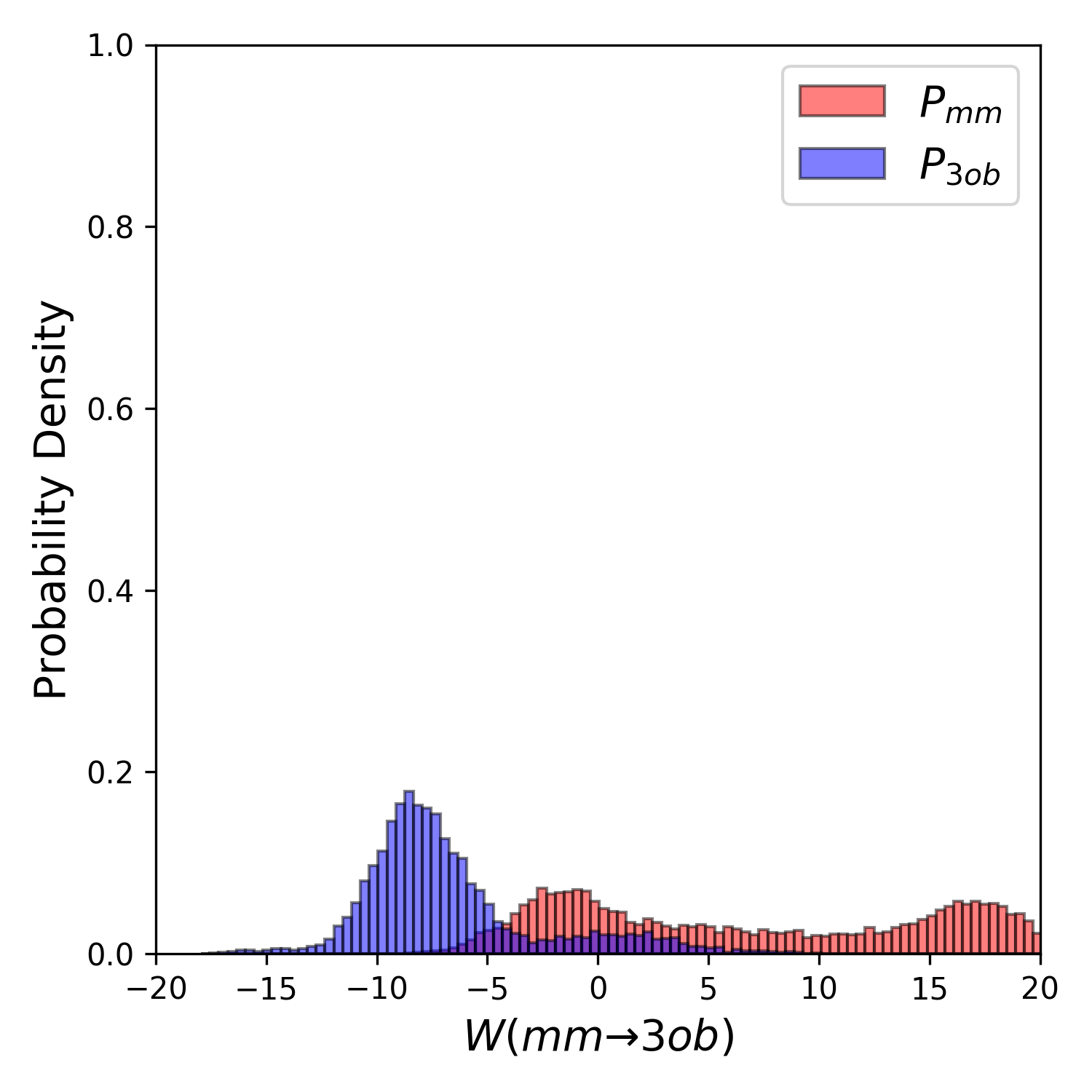

Supplement: Supplementary file 1 [file molecules-24-00681-s001.zip › final-SI/images/zinc_00123162-mm-3ob-jar-overlap.png]

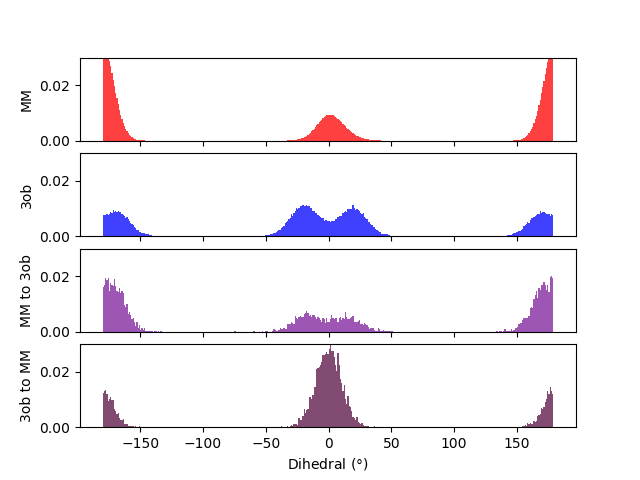

Supplement: Supplementary file 1 [file molecules-24-00681-s001.zip › final-SI/images/zinc_04344392-mm-3ob-subplots-dihe-chi3.png]

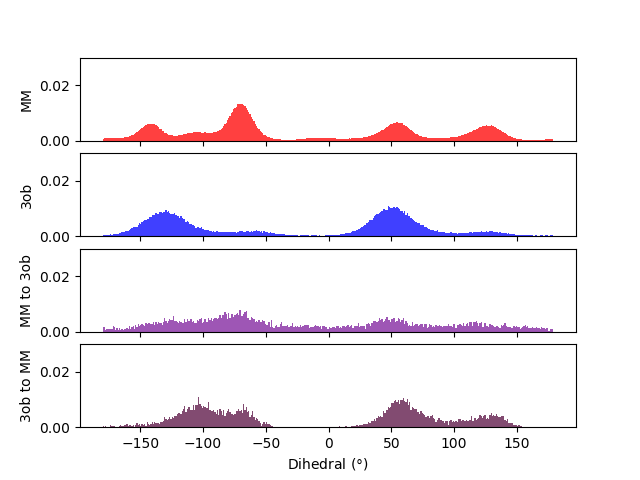

Supplement: Supplementary file 1 [file molecules-24-00681-s001.zip › final-SI/images/zinc_00087557-mm-3ob-subplots-dihe-chi1.png]

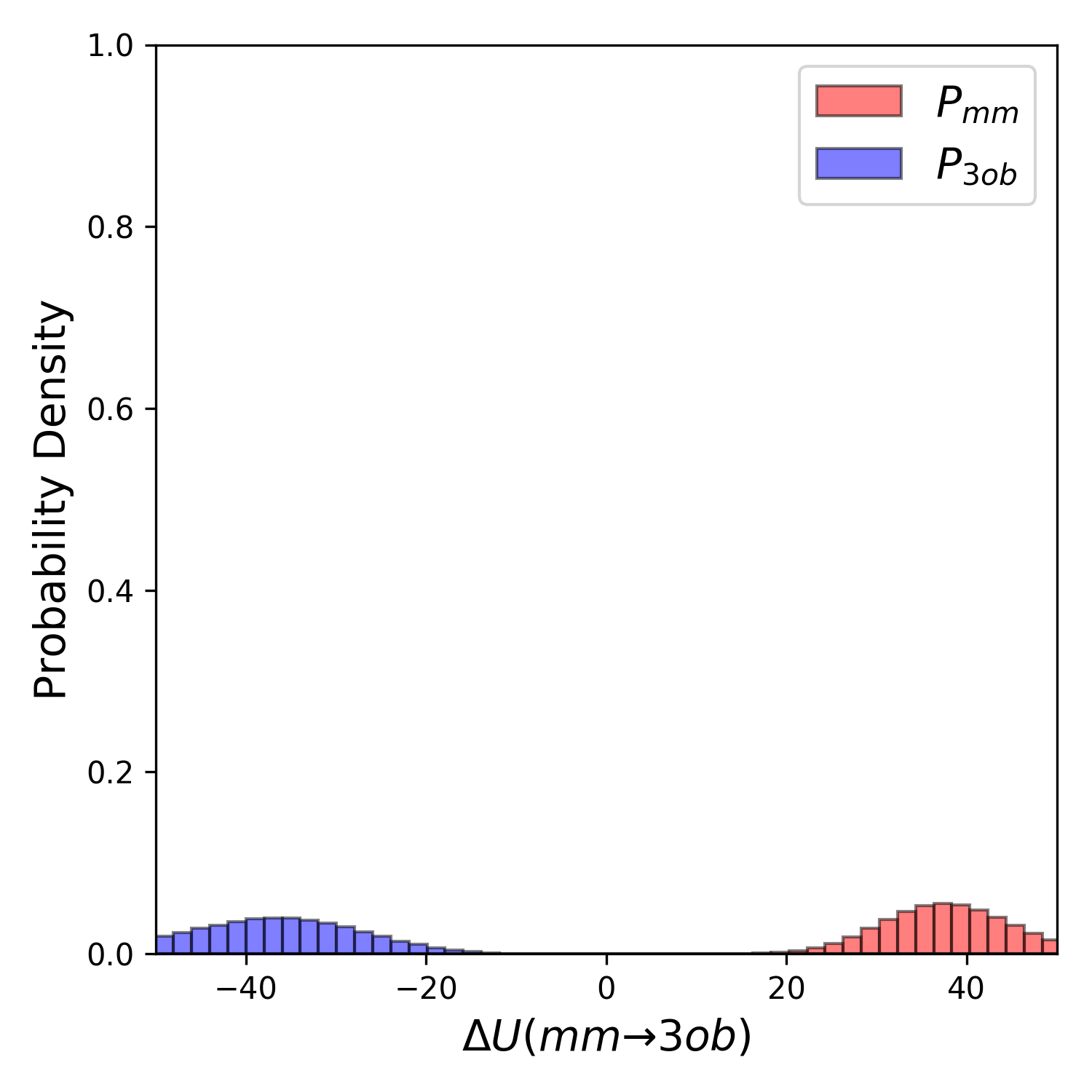

Supplement: Supplementary file 1 [file molecules-24-00681-s001.zip › final-SI/images/zinc_00095858-mm-3ob-fep-overlap.png]

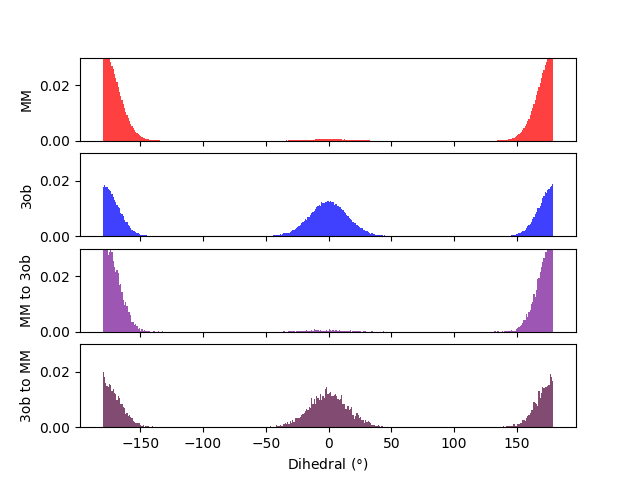

Supplement: Supplementary file 1 [file molecules-24-00681-s001.zip › final-SI/images/zinc_00077329-mm-3ob-subplots-dihe-chi1.png]

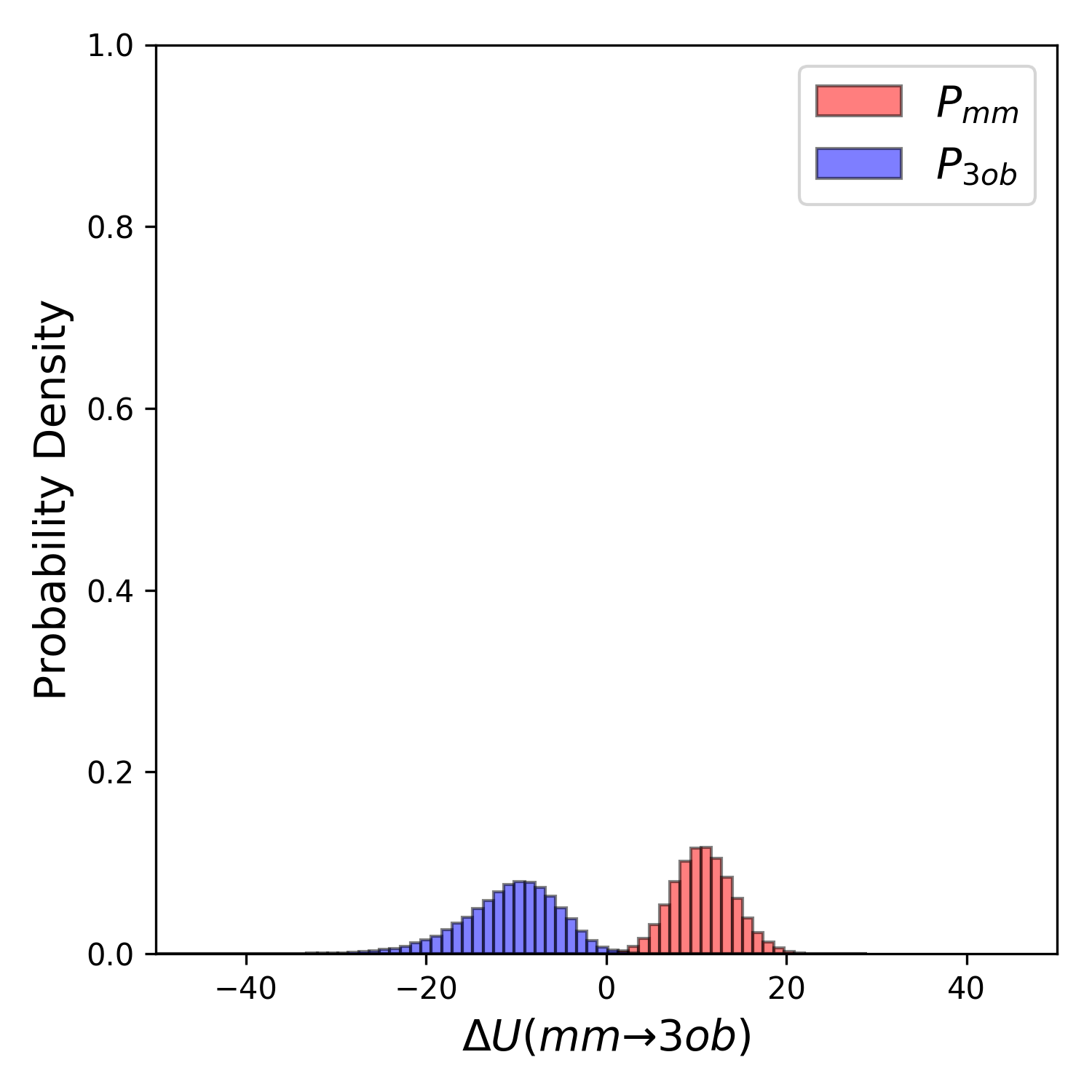

Supplement: Supplementary file 1 [file molecules-24-00681-s001.zip › final-SI/images/zinc_00169358-mm-3ob-fep-overlap.png]

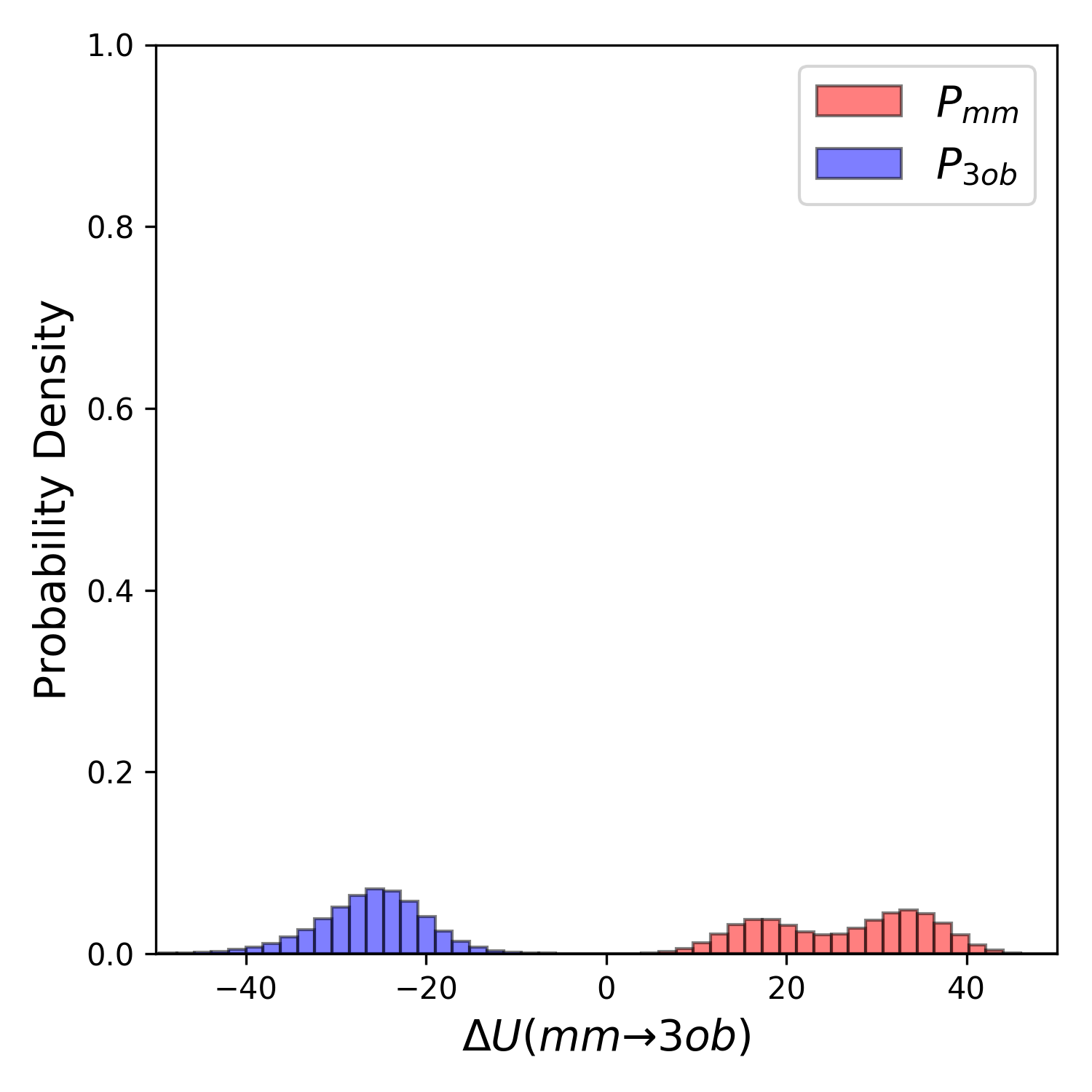

Supplement: Supplementary file 1 [file molecules-24-00681-s001.zip › final-SI/images/zinc_00123162-mm-3ob-fep-overlap.png]

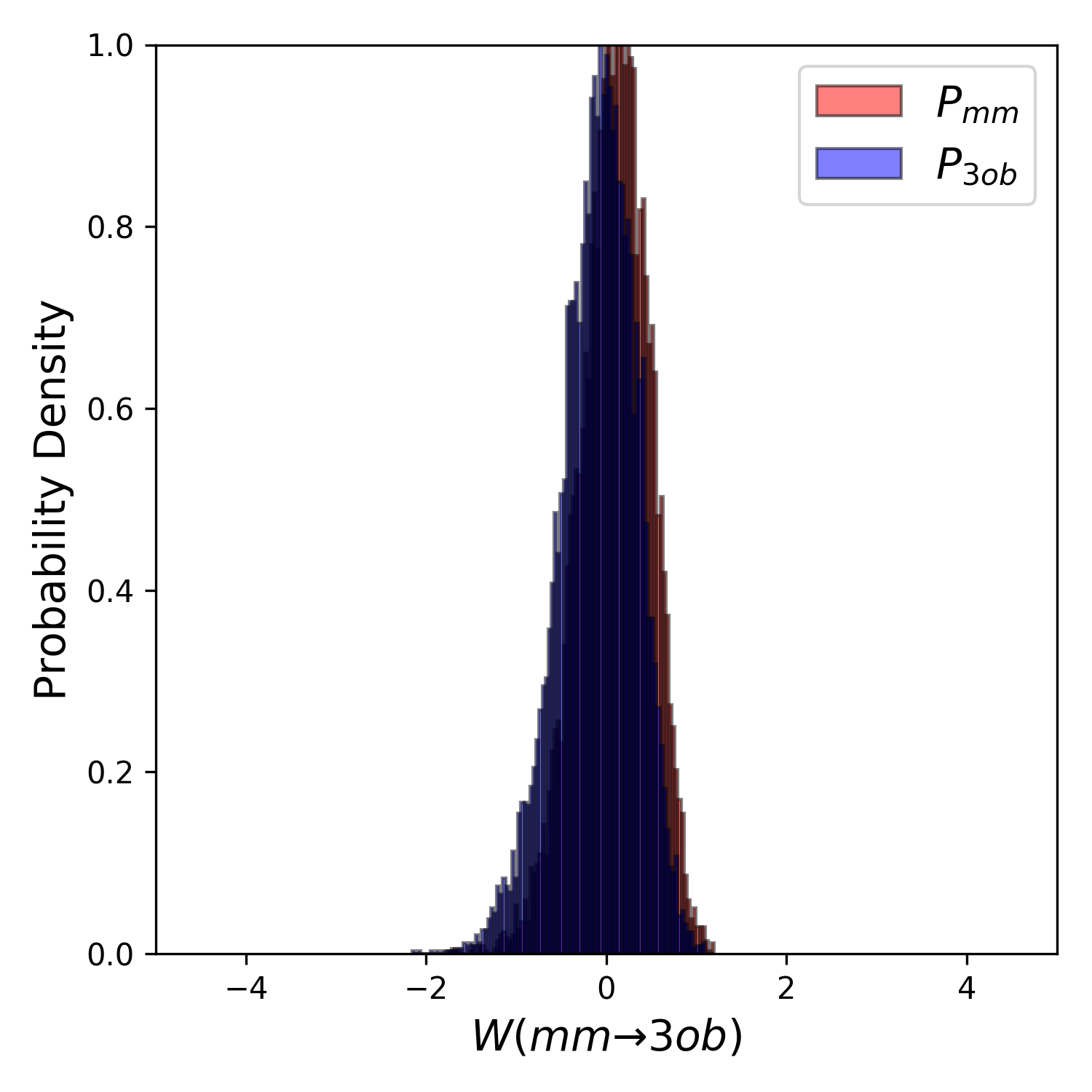

Supplement: Supplementary file 1 [file molecules-24-00681-s001.zip › final-SI/images/zinc_00169358-mm-3ob-jar-overlap.png]

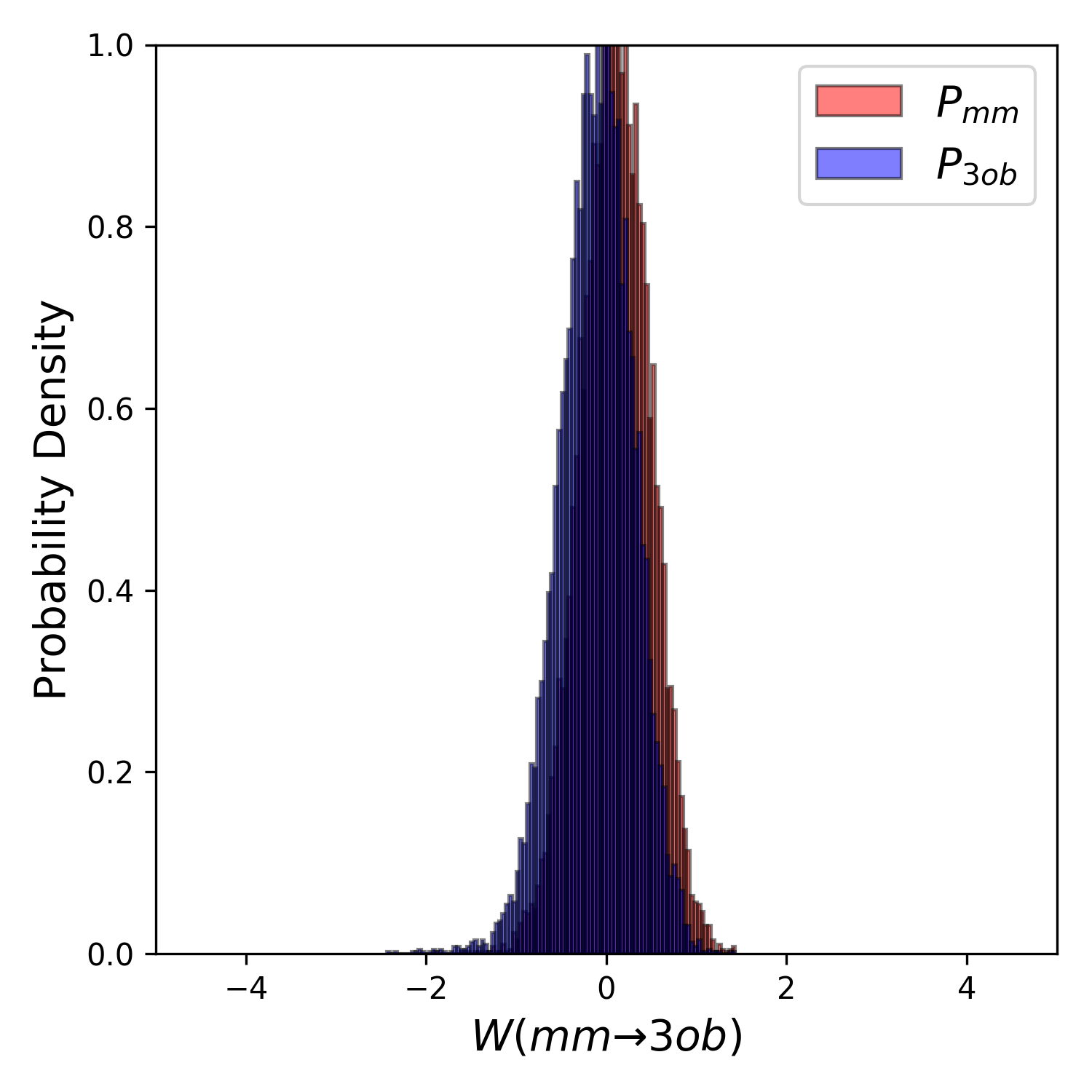

Supplement: Supplementary file 1 [file molecules-24-00681-s001.zip › final-SI/images/zinc_01867000-mm-3ob-jar-overlap.png]

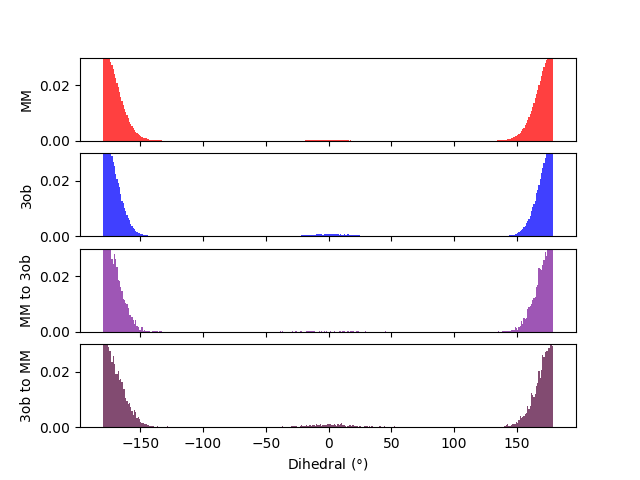

Supplement: Supplementary file 1 [file molecules-24-00681-s001.zip › final-SI/images/zinc_04344392-mm-3ob-subplots-dihe-chi2.png]

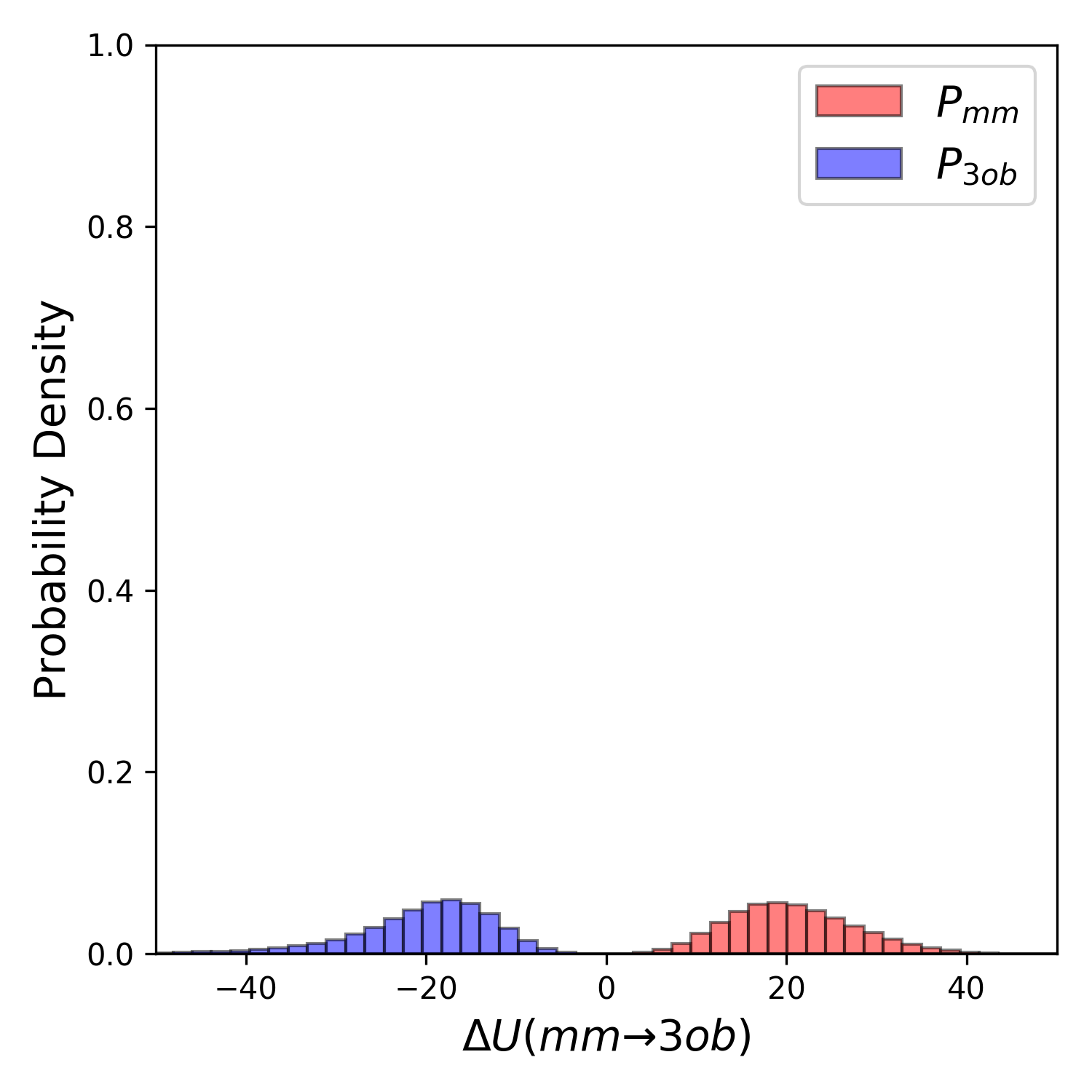

Supplement: Supplementary file 1 [file molecules-24-00681-s001.zip › final-SI/images/zinc_00138607-mm-3ob-fep-overlap.png]

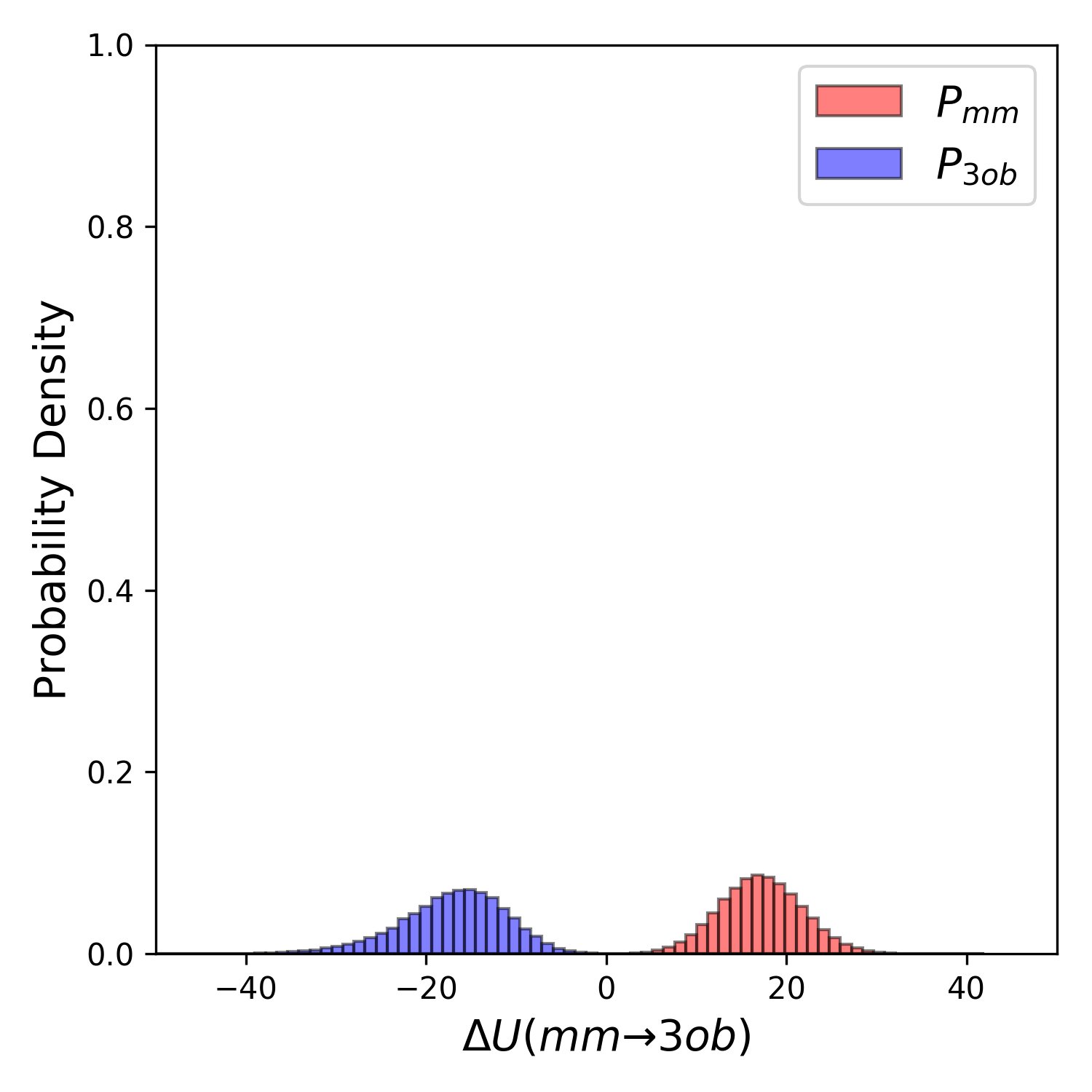

Supplement: Supplementary file 1 [file molecules-24-00681-s001.zip › final-SI/images/zinc_00167648-mm-3ob-fep-overlap.png]

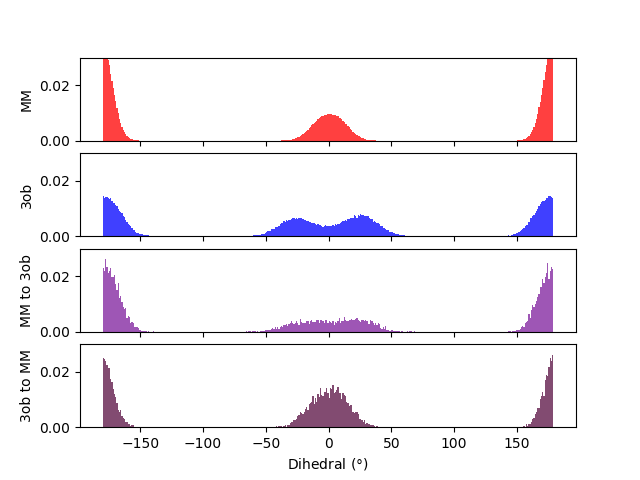

Supplement: Supplementary file 1 [file molecules-24-00681-s001.zip › final-SI/images/zinc_03127671-mm-3ob-subplots-dihe-chi9.png]

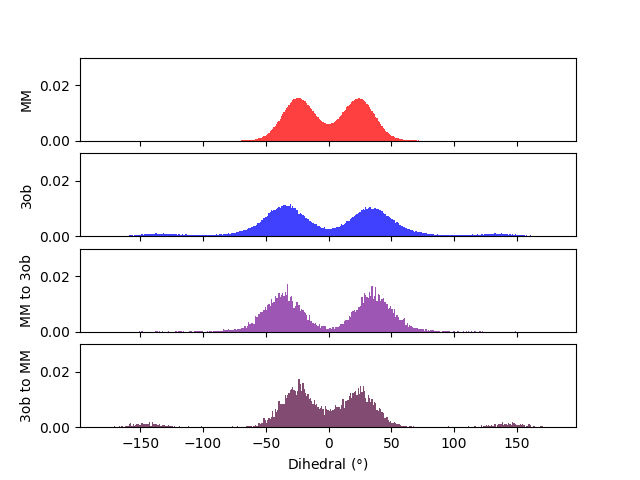

Supplement: Supplementary file 1 [file molecules-24-00681-s001.zip › final-SI/images/zinc_00133435-mm-3ob-subplots-dihe-chi1.png]

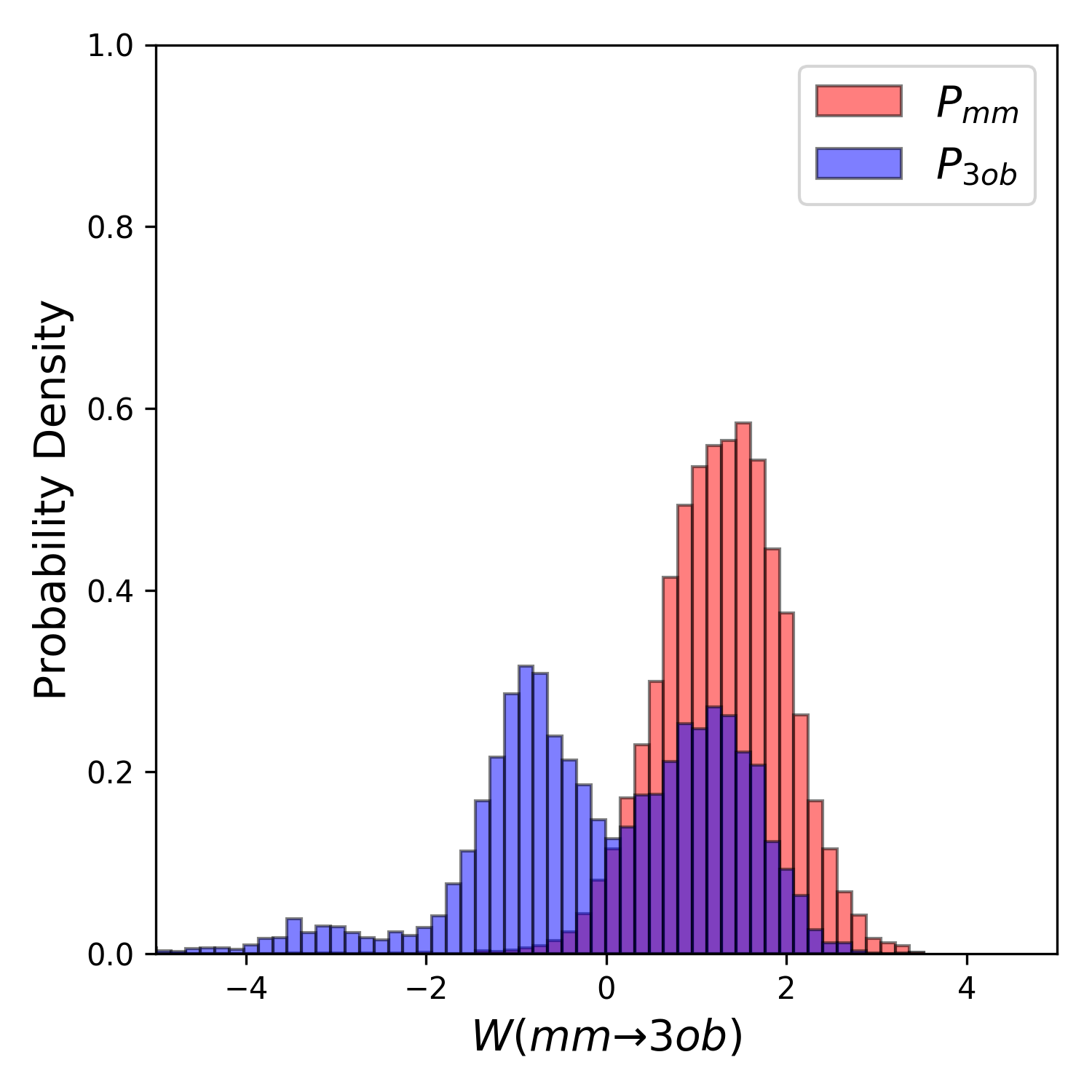

Supplement: Supplementary file 1 [file molecules-24-00681-s001.zip › final-SI/images/zinc_00107550-mm-3ob-jar-overlap.png]

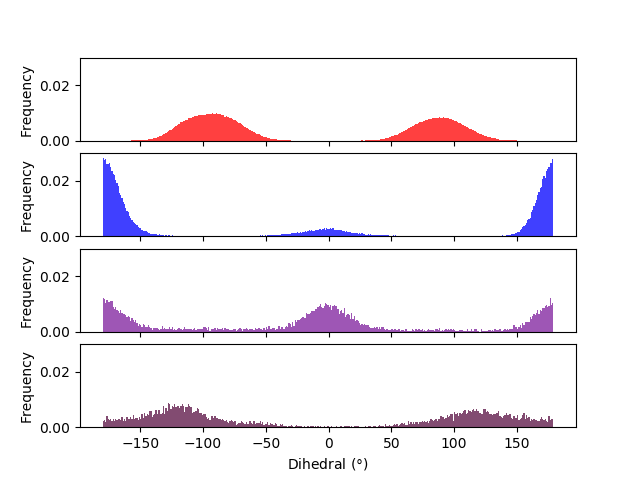

Supplement: Supplementary file 1 [file molecules-24-00681-s001.zip › final-SI/images/zinc_00123162-mm-3ob-subplots-dihe-chi6.png]

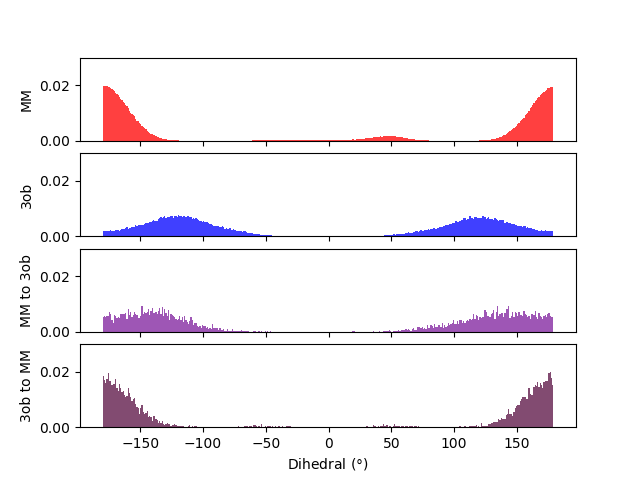

Supplement: Supplementary file 1 [file molecules-24-00681-s001.zip › final-SI/images/zinc_04344392-mm-3ob-subplots-dihe-chi4.png]

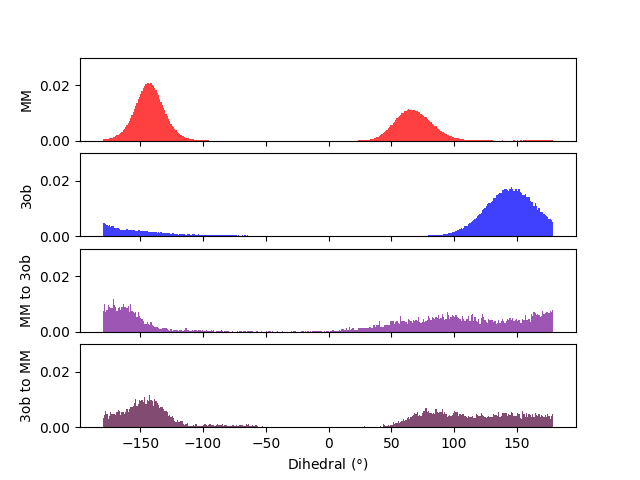

Supplement: Supplementary file 1 [file molecules-24-00681-s001.zip › final-SI/images/zinc_00087557-mm-3ob-subplots-dihe-chi3.png]

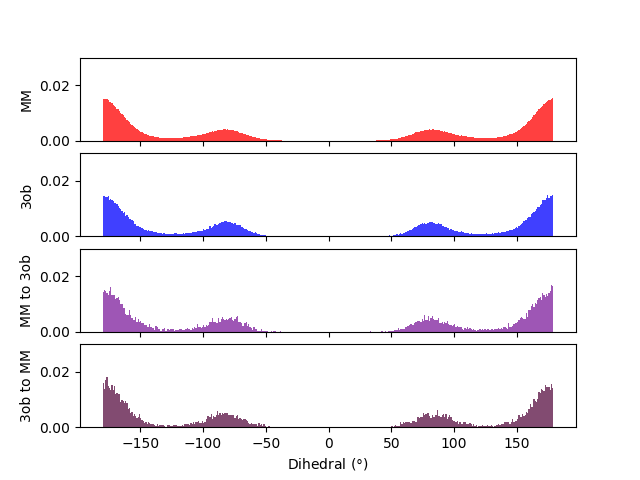

Supplement: Supplementary file 1 [file molecules-24-00681-s001.zip › final-SI/images/zinc_00061095-mm-3ob-subplots-dihe-chi1.png]

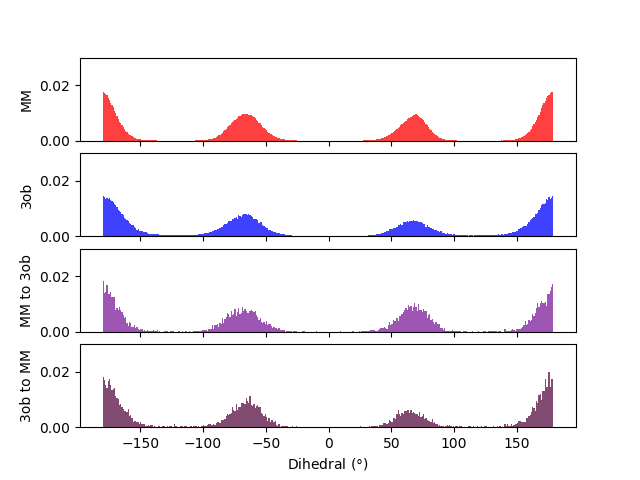

Supplement: Supplementary file 1 [file molecules-24-00681-s001.zip › final-SI/images/zinc_04344392-mm-3ob-subplots-dihe-chi8.png]

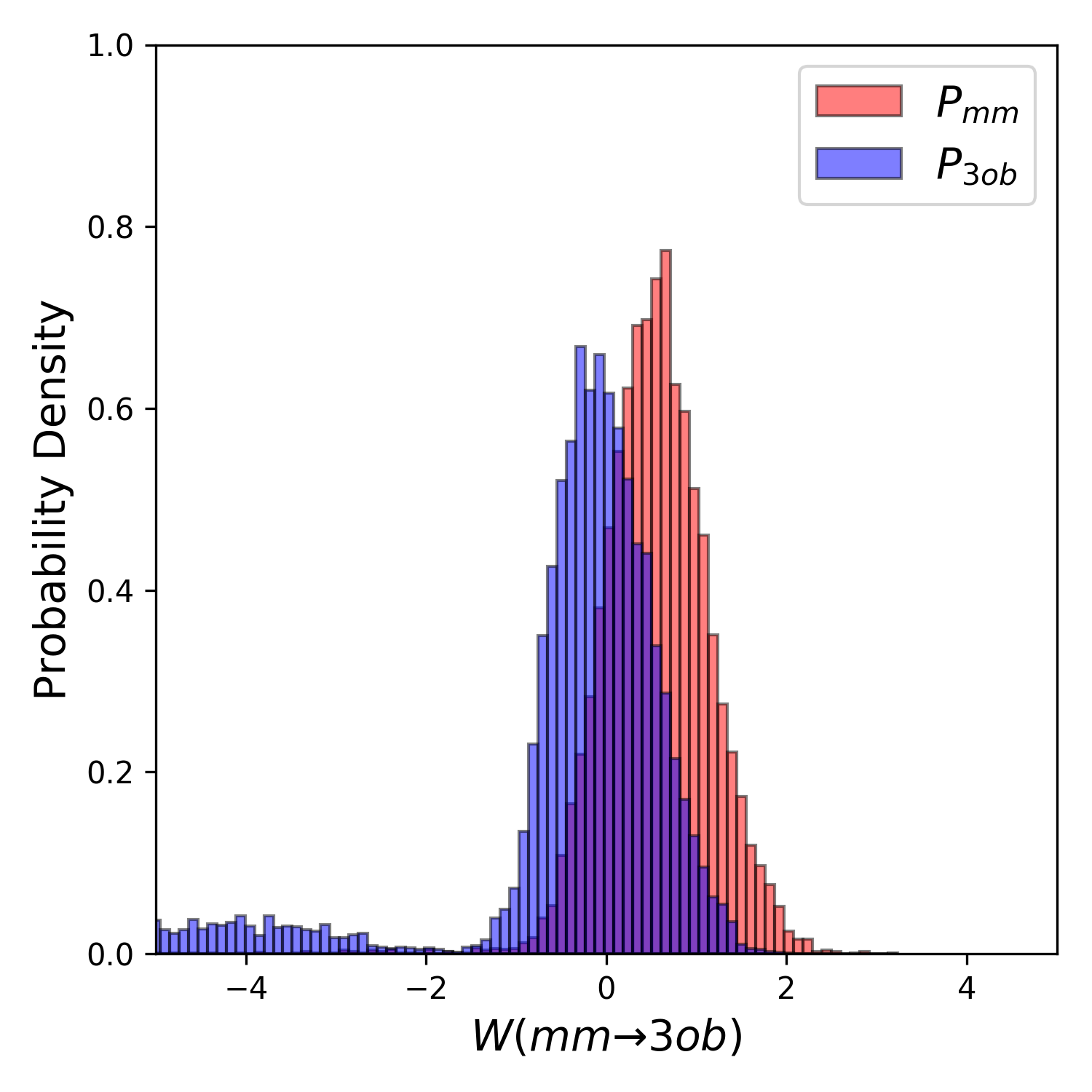

Supplement: Supplementary file 1 [file molecules-24-00681-s001.zip › final-SI/images/zinc_00077329-mm-3ob-jar-overlap.png]

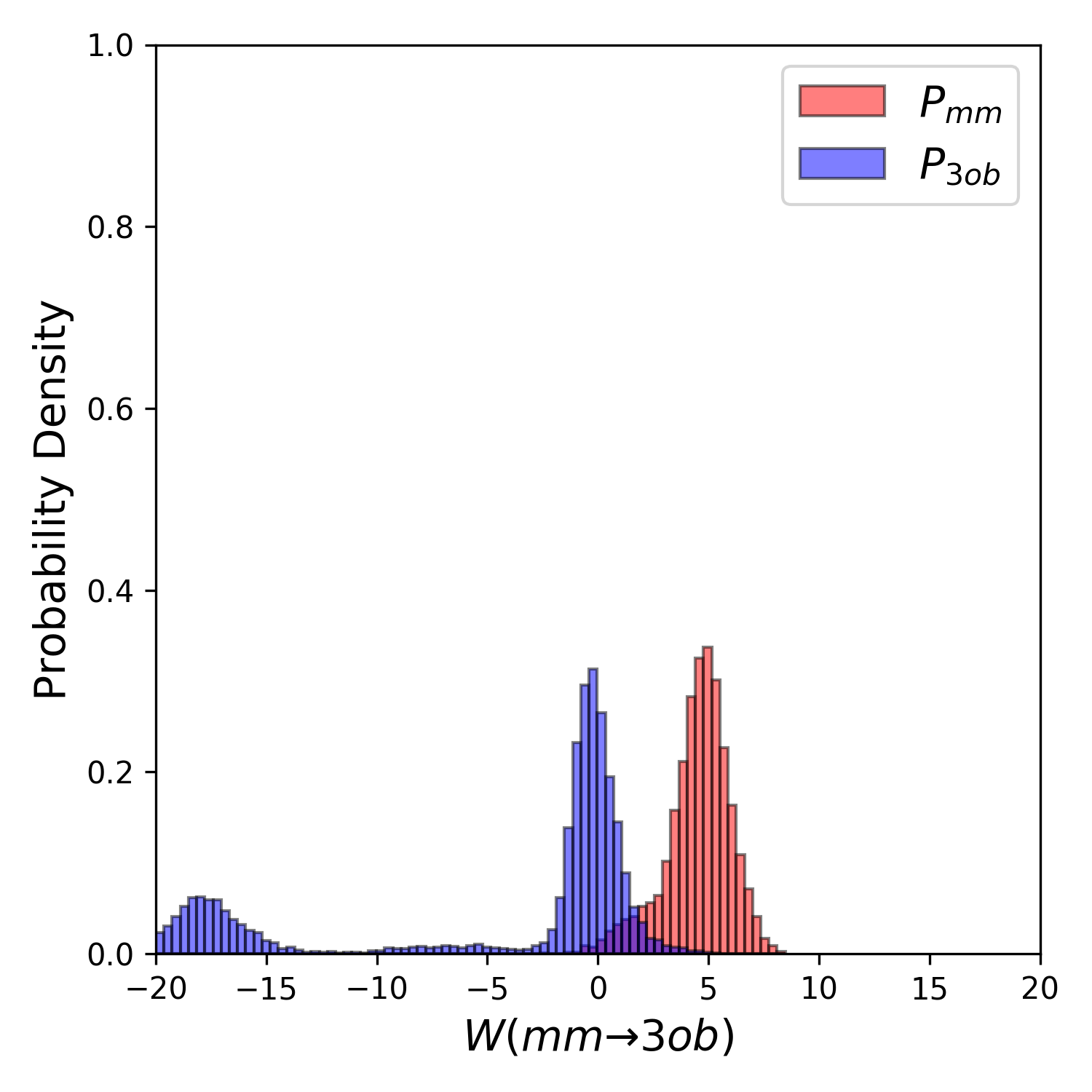

Supplement: Supplementary file 1 [file molecules-24-00681-s001.zip › final-SI/images/zinc_33381936-mm-3ob-jar-overlap.png]

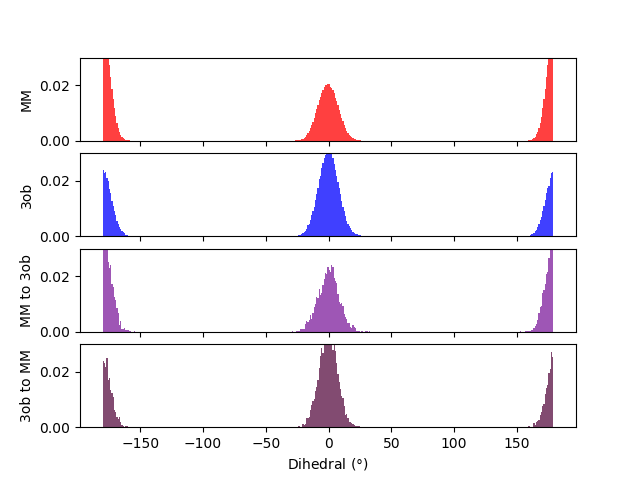

Supplement: Supplementary file 1 [file molecules-24-00681-s001.zip › final-SI/images/zinc_00107778-mm-3ob-subplots-dihe-chi1.png]

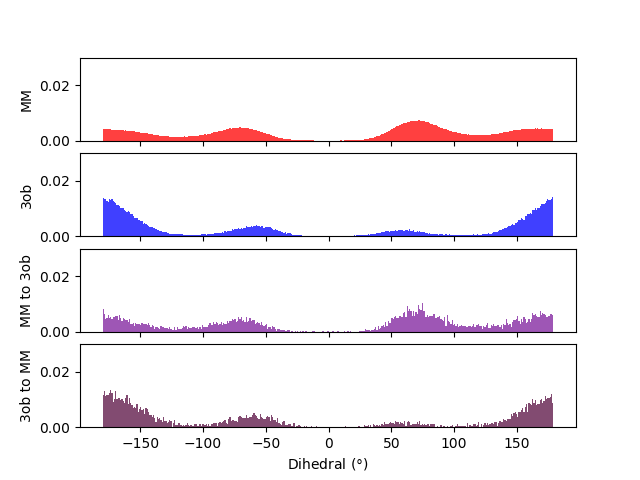

Supplement: Supplementary file 1 [file molecules-24-00681-s001.zip › final-SI/images/zinc_00167648-mm-3ob-subplots-dihe-chi2.png]

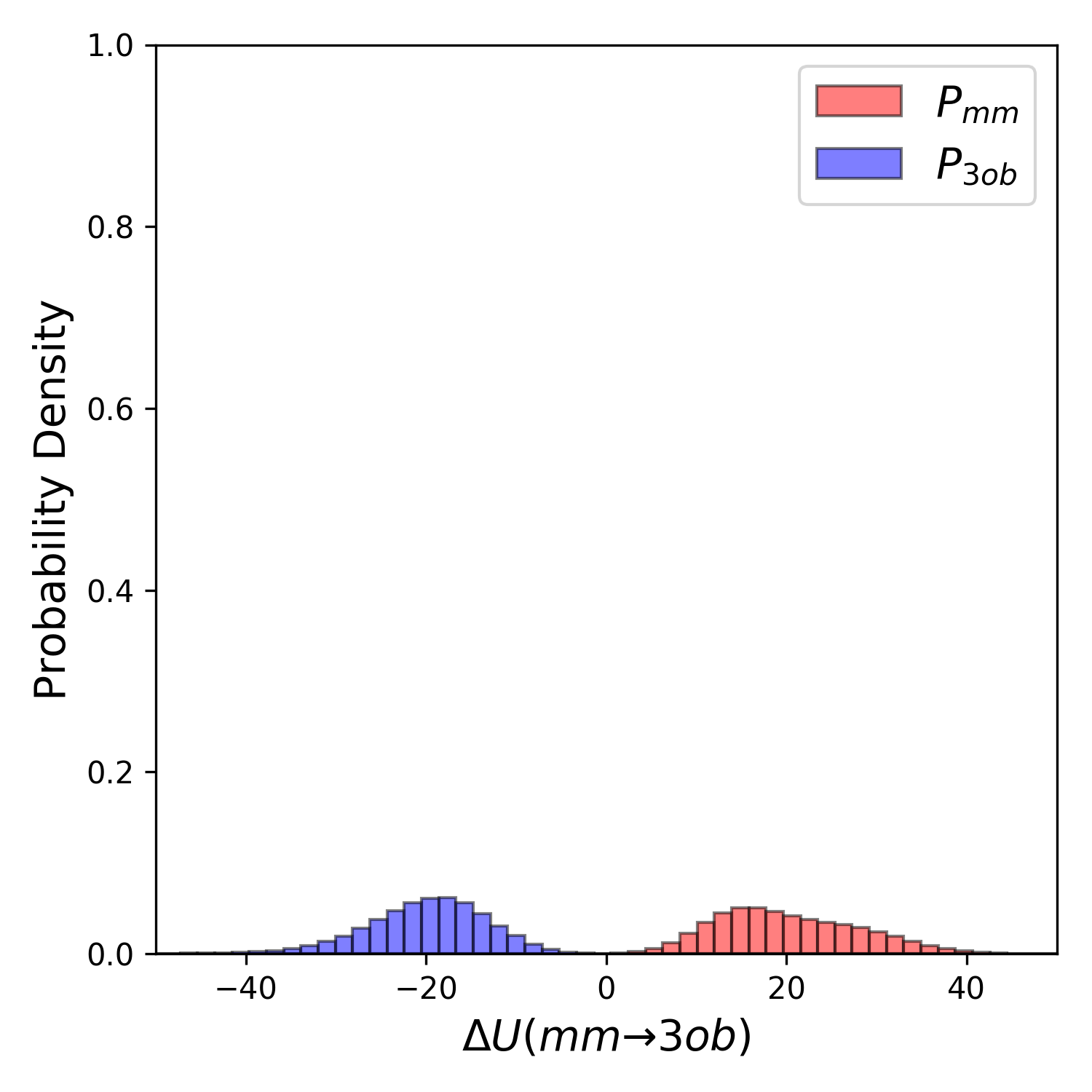

Supplement: Supplementary file 1 [file molecules-24-00681-s001.zip › final-SI/images/zinc_00061095-mm-3ob-fep-overlap.png]

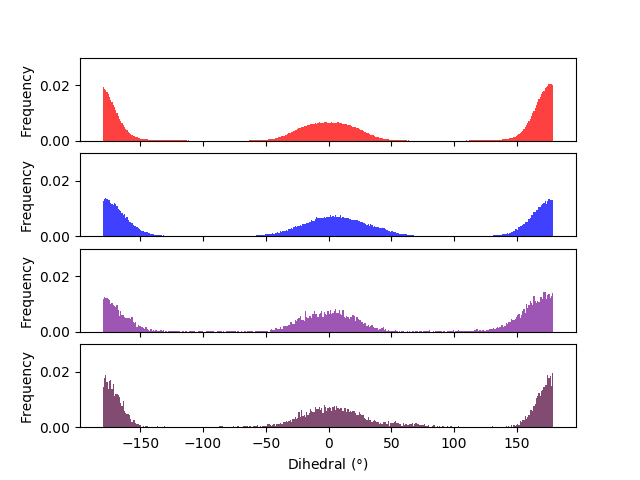

Supplement: Supplementary file 1 [file molecules-24-00681-s001.zip › final-SI/images/zinc_00123162-mm-3ob-subplots-dihe-chi2.png]

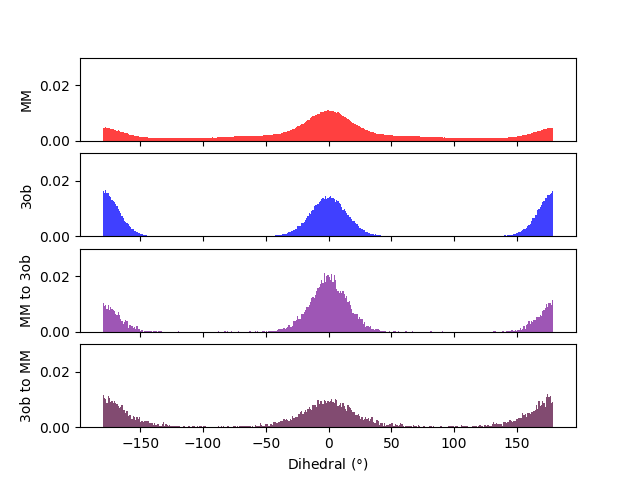

Supplement: Supplementary file 1 [file molecules-24-00681-s001.zip › final-SI/images/zinc_00061095-mm-3ob-subplots-dihe-chi2.png]

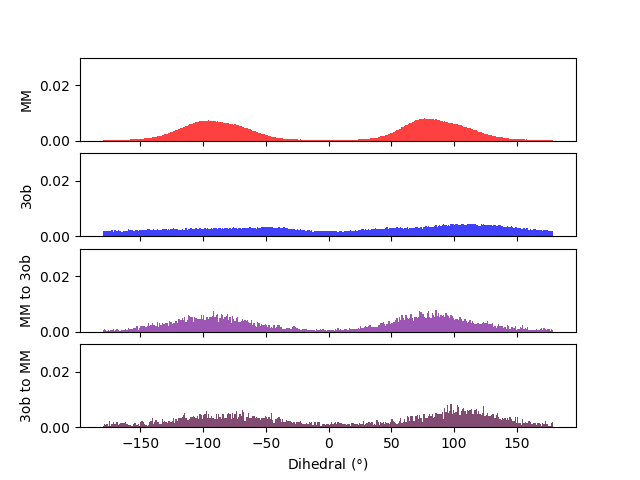

Supplement: Supplementary file 1 [file molecules-24-00681-s001.zip › final-SI/images/zinc_00167648-mm-3ob-subplots-dihe-chi3.png]

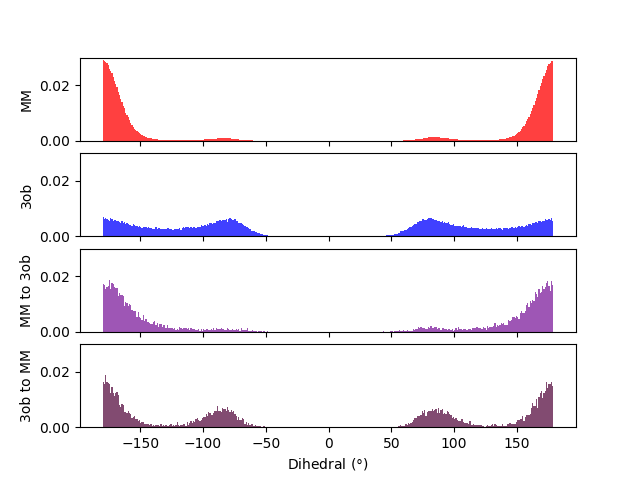

Supplement: Supplementary file 1 [file molecules-24-00681-s001.zip › final-SI/images/zinc_00095858-mm-3ob-subplots-dihe-chi3.png]

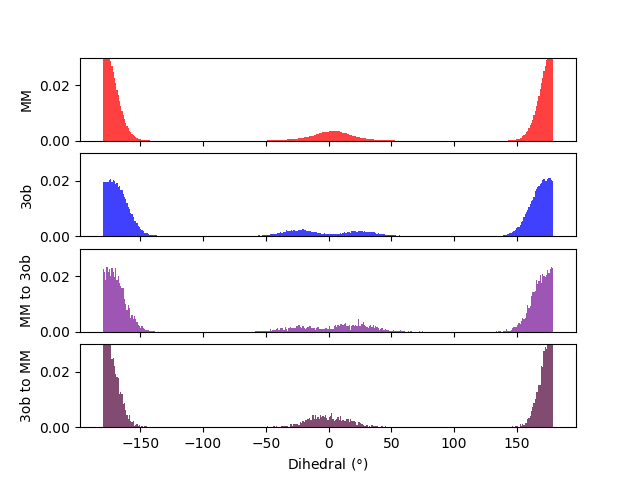

Supplement: Supplementary file 1 [file molecules-24-00681-s001.zip › final-SI/images/zinc_04344392-mm-3ob-subplots-dihe-chi5.png]

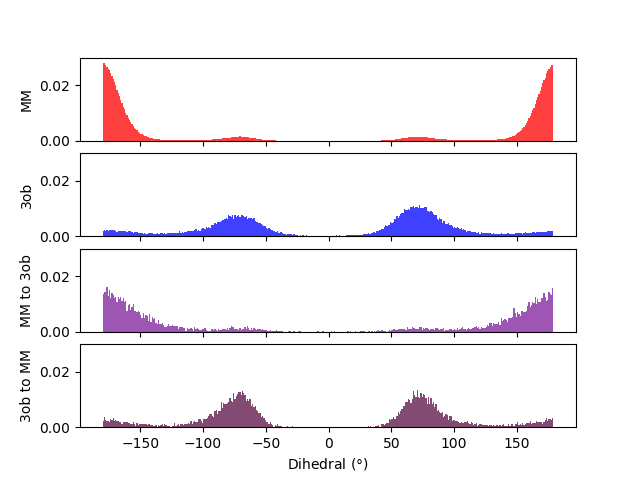

Supplement: Supplementary file 1 [file molecules-24-00681-s001.zip › final-SI/images/zinc_33381936-mm-3ob-subplots-dihe-chi1.png]

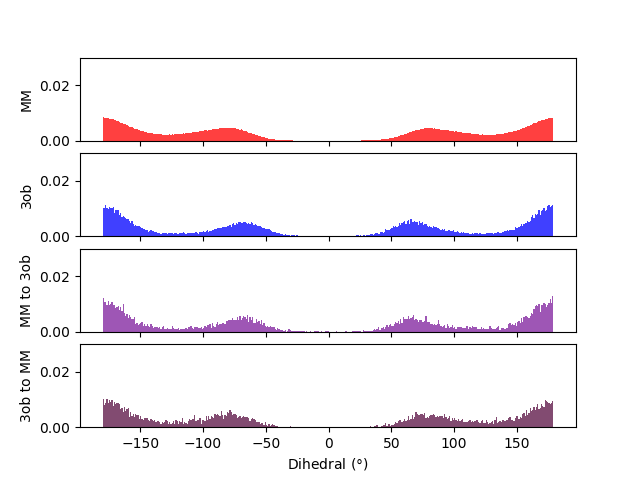

Supplement: Supplementary file 1 [file molecules-24-00681-s001.zip › final-SI/images/zinc_00164361-mm-3ob-subplots-dihe-chi1.png]

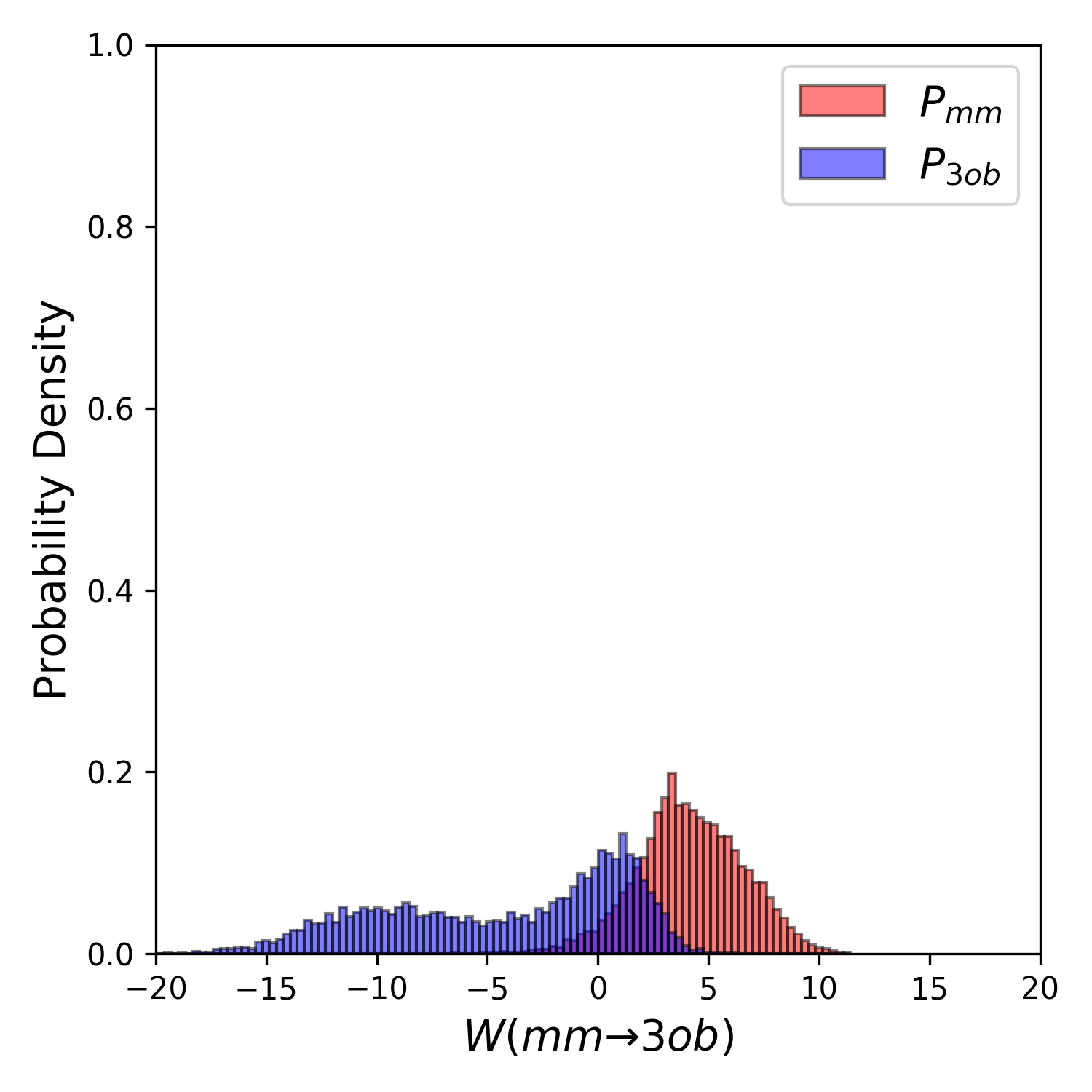

Supplement: Supplementary file 1 [file molecules-24-00681-s001.zip › final-SI/images/zinc_03127671-mm-3ob-jar-overlap.png]

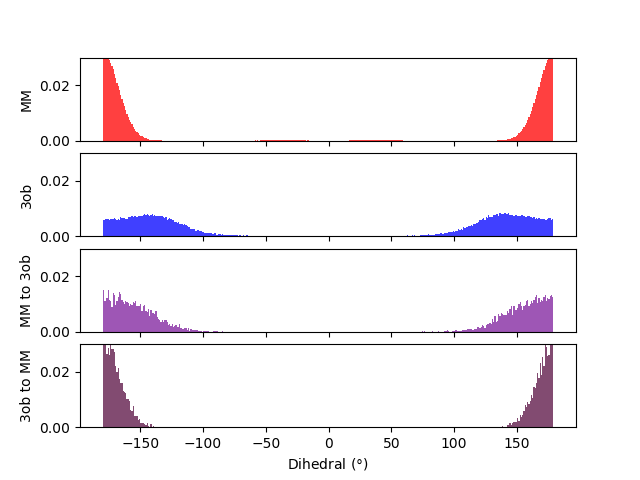

Supplement: Supplementary file 1 [file molecules-24-00681-s001.zip › final-SI/images/zinc_06568023-mm-3ob-subplots-dihe-chi2.png]

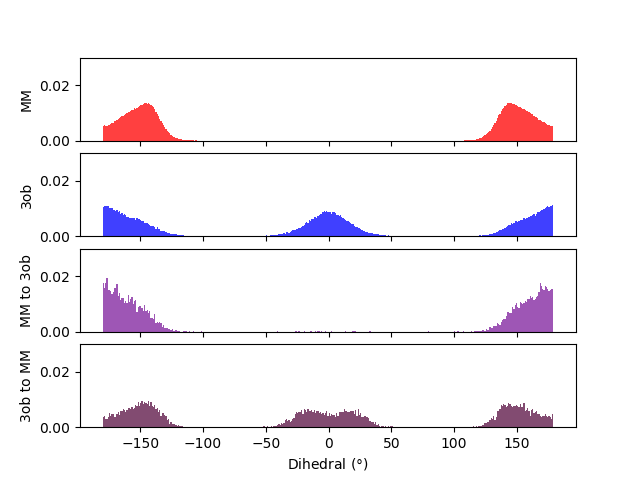

Supplement: Supplementary file 1 [file molecules-24-00681-s001.zip › final-SI/images/zinc_00107550-mm-3ob-subplots-dihe-chi2.png]

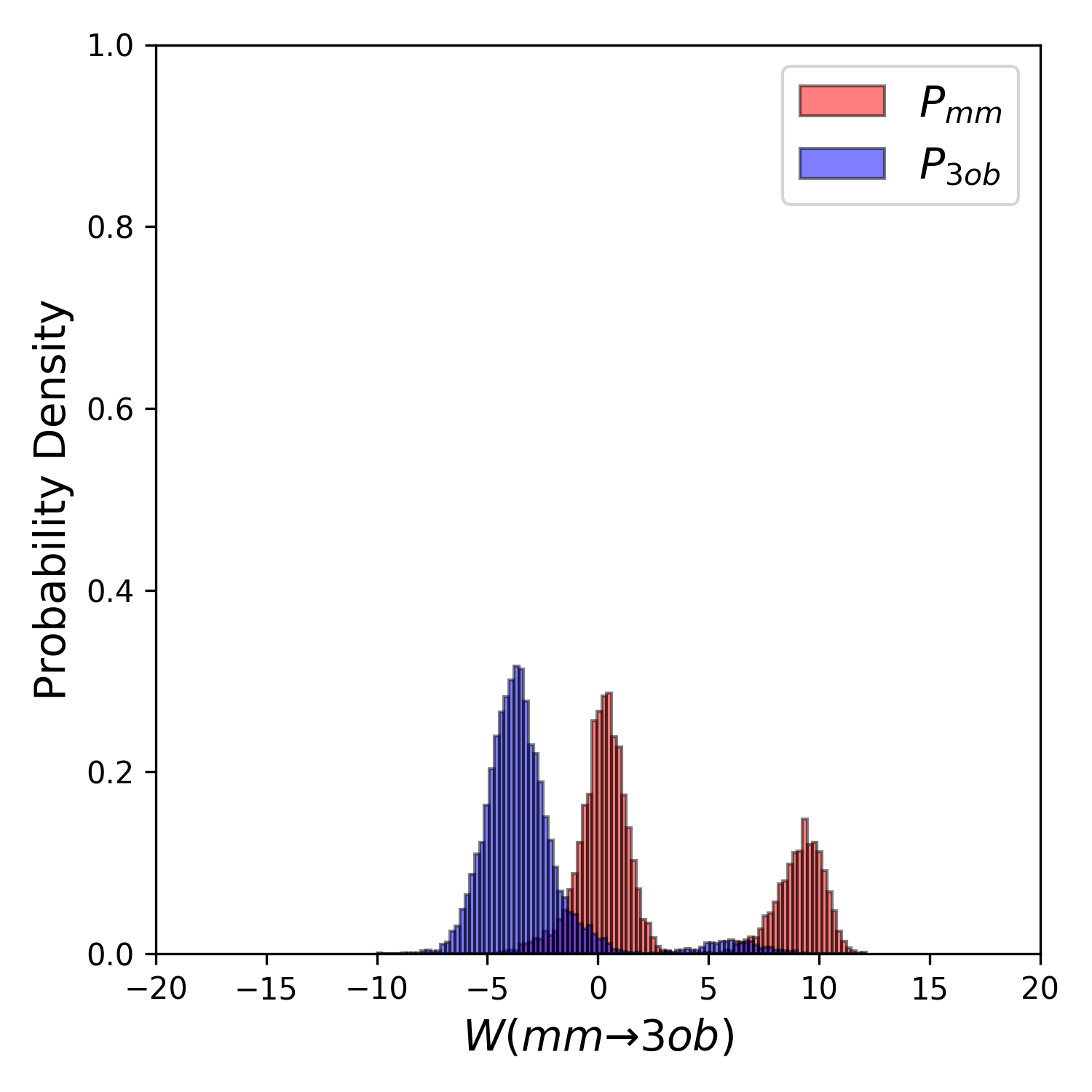

Supplement: Supplementary file 1 [file molecules-24-00681-s001.zip › final-SI/images/zinc_04363792-mm-3ob-jar-overlap.png]

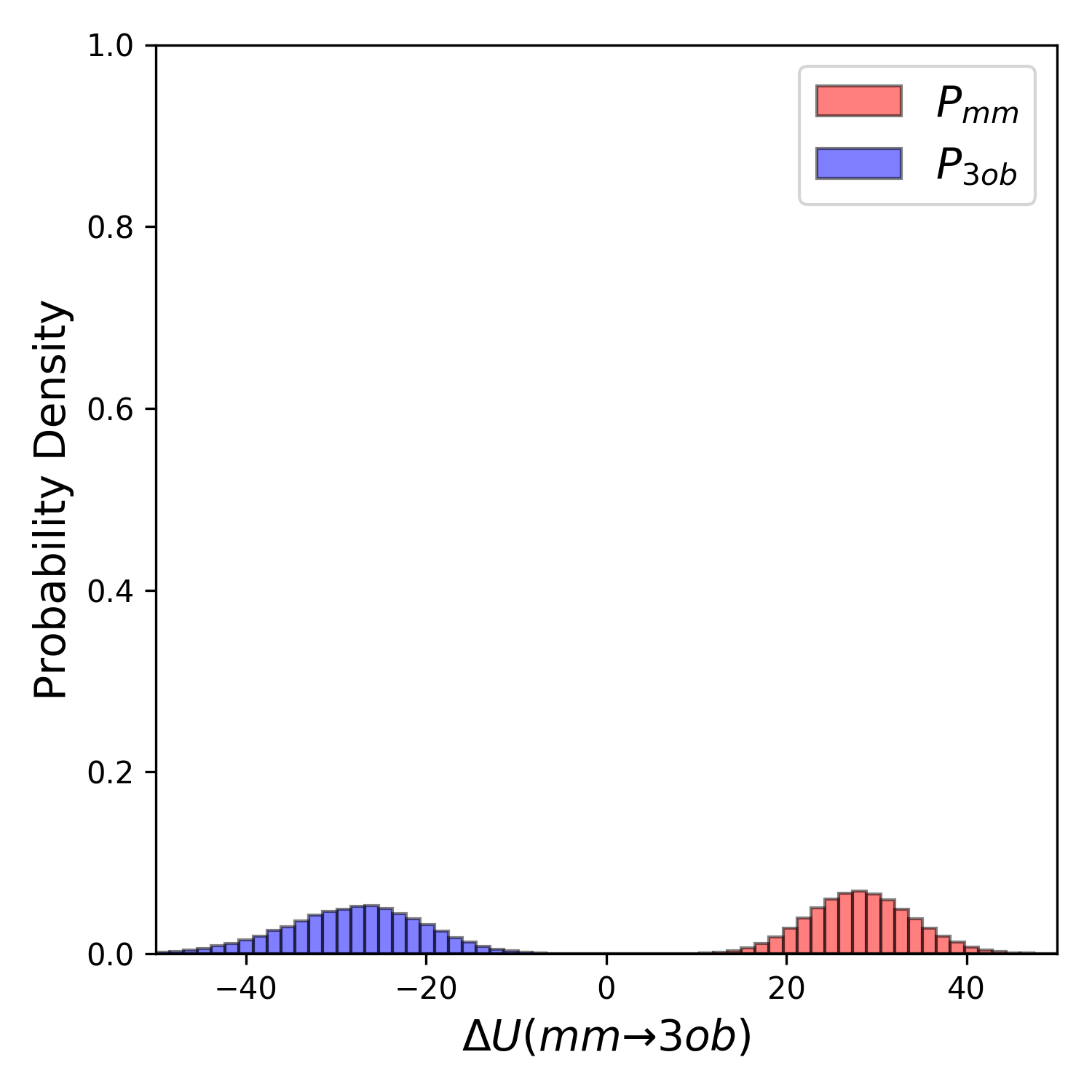

Supplement: Supplementary file 1 [file molecules-24-00681-s001.zip › final-SI/images/zinc_00087557-mm-3ob-fep-overlap.png]

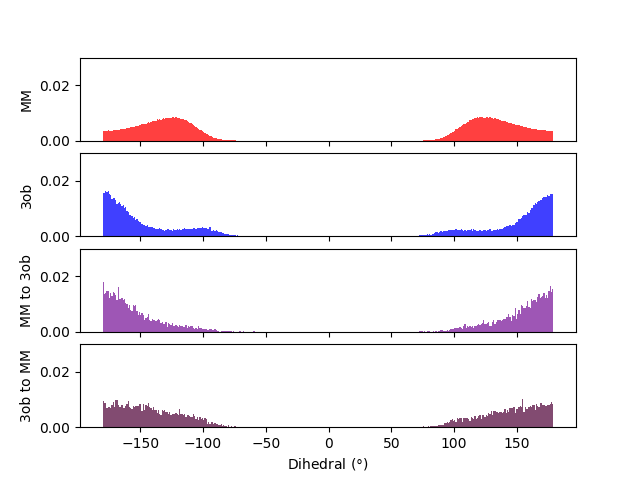

Supplement: Supplementary file 1 [file molecules-24-00681-s001.zip › final-SI/images/zinc_33381936-mm-3ob-subplots-dihe-chi2.png]

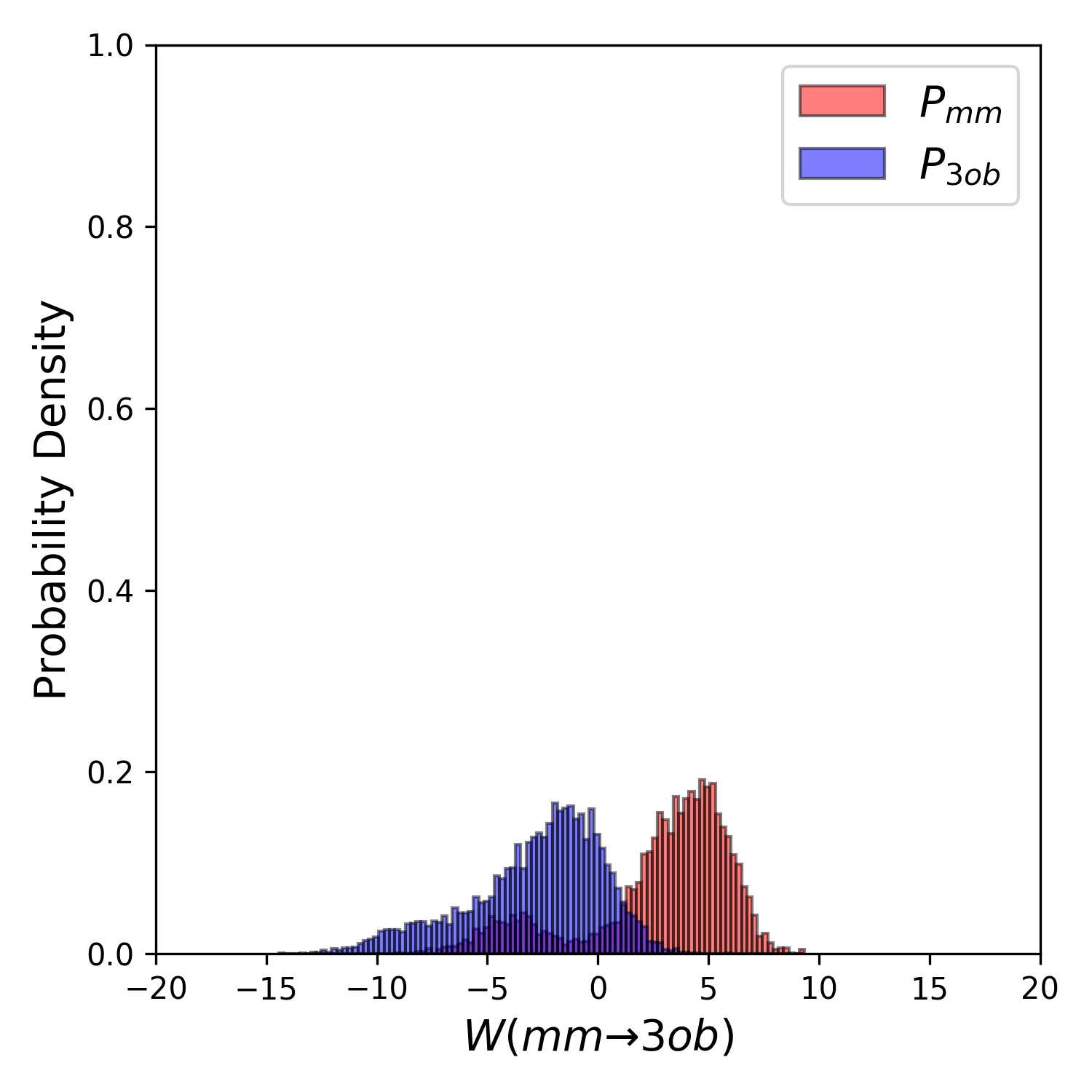

Supplement: Supplementary file 1 [file molecules-24-00681-s001.zip › final-SI/images/zinc_04344392-mm-3ob-jar-overlap.png]

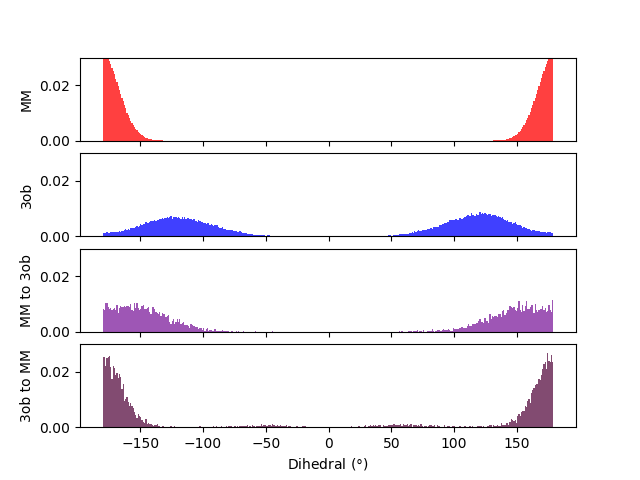

Supplement: Supplementary file 1 [file molecules-24-00681-s001.zip › final-SI/images/zinc_01755198-mm-3ob-subplots-dihe-chi2.png]

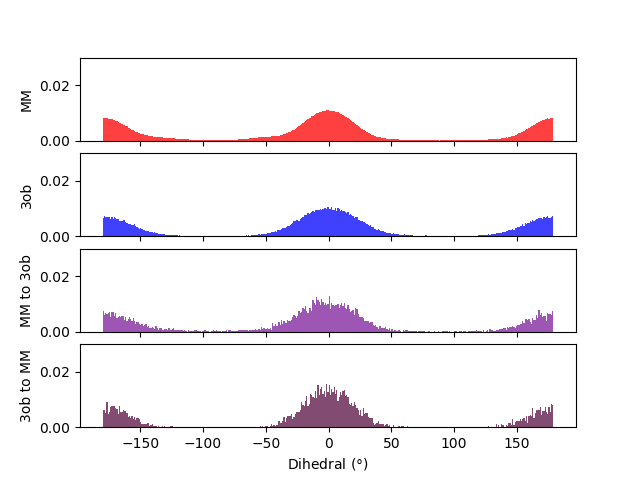

Supplement: Supplementary file 1 [file molecules-24-00681-s001.zip › final-SI/images/zinc_06568023-mm-3ob-subplots-dihe-chi1.png]

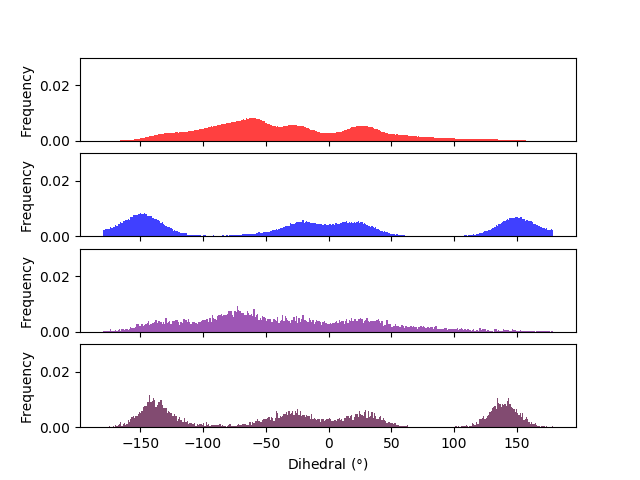

Supplement: Supplementary file 1 [file molecules-24-00681-s001.zip › final-SI/images/zinc_00123162-mm-3ob-subplots-dihe-chi4.png]

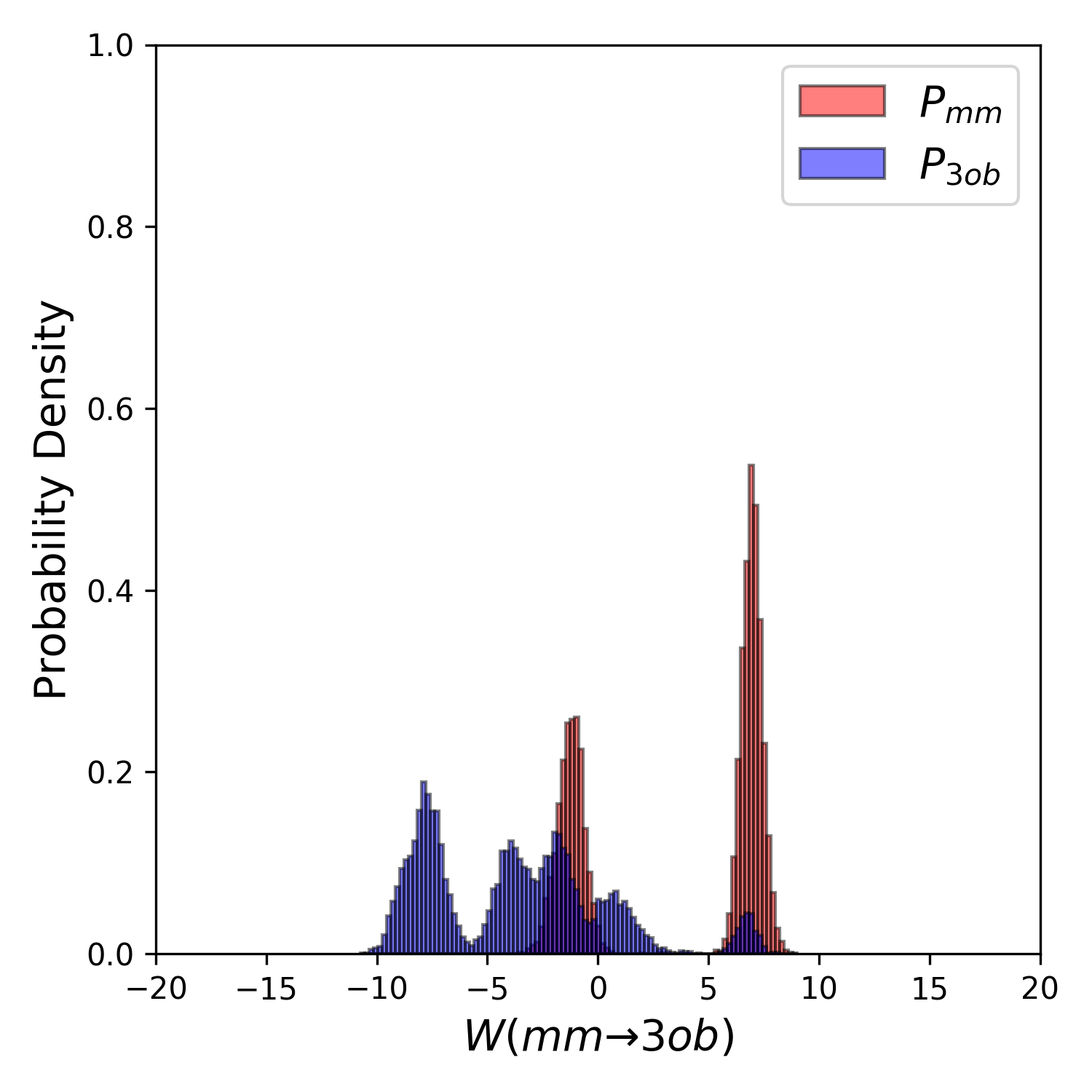

Supplement: Supplementary file 1 [file molecules-24-00681-s001.zip › final-SI/images/zinc_00107778-mm-3ob-jar-overlap.png]

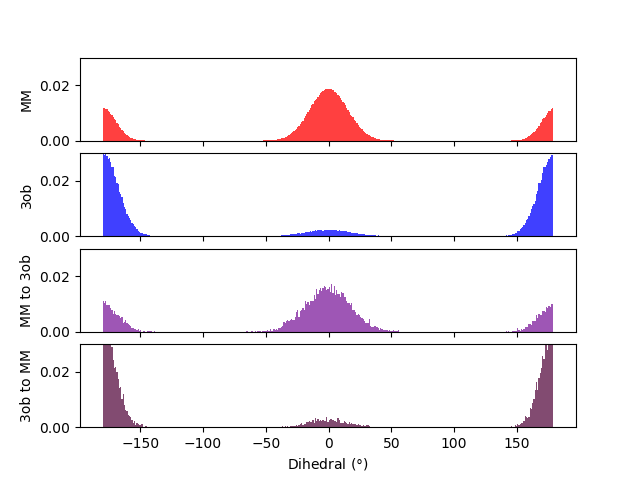

Supplement: Supplementary file 1 [file molecules-24-00681-s001.zip › final-SI/images/zinc_03127671-mm-3ob-subplots-dihe-chi2.png]

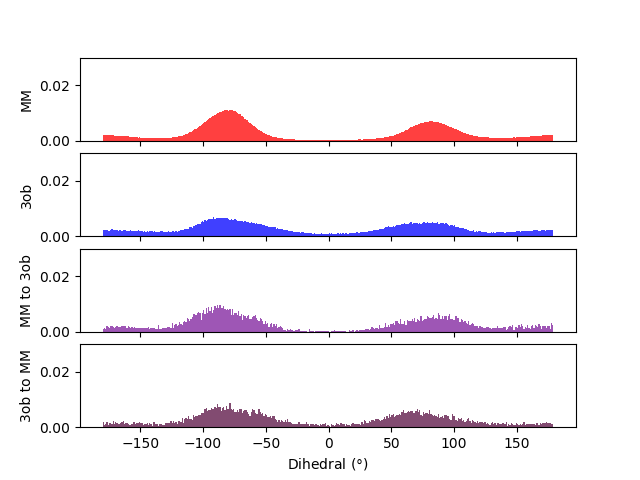

Supplement: Supplementary file 1 [file molecules-24-00681-s001.zip › final-SI/images/zinc_03127671-mm-3ob-subplots-dihe-chi3.png]

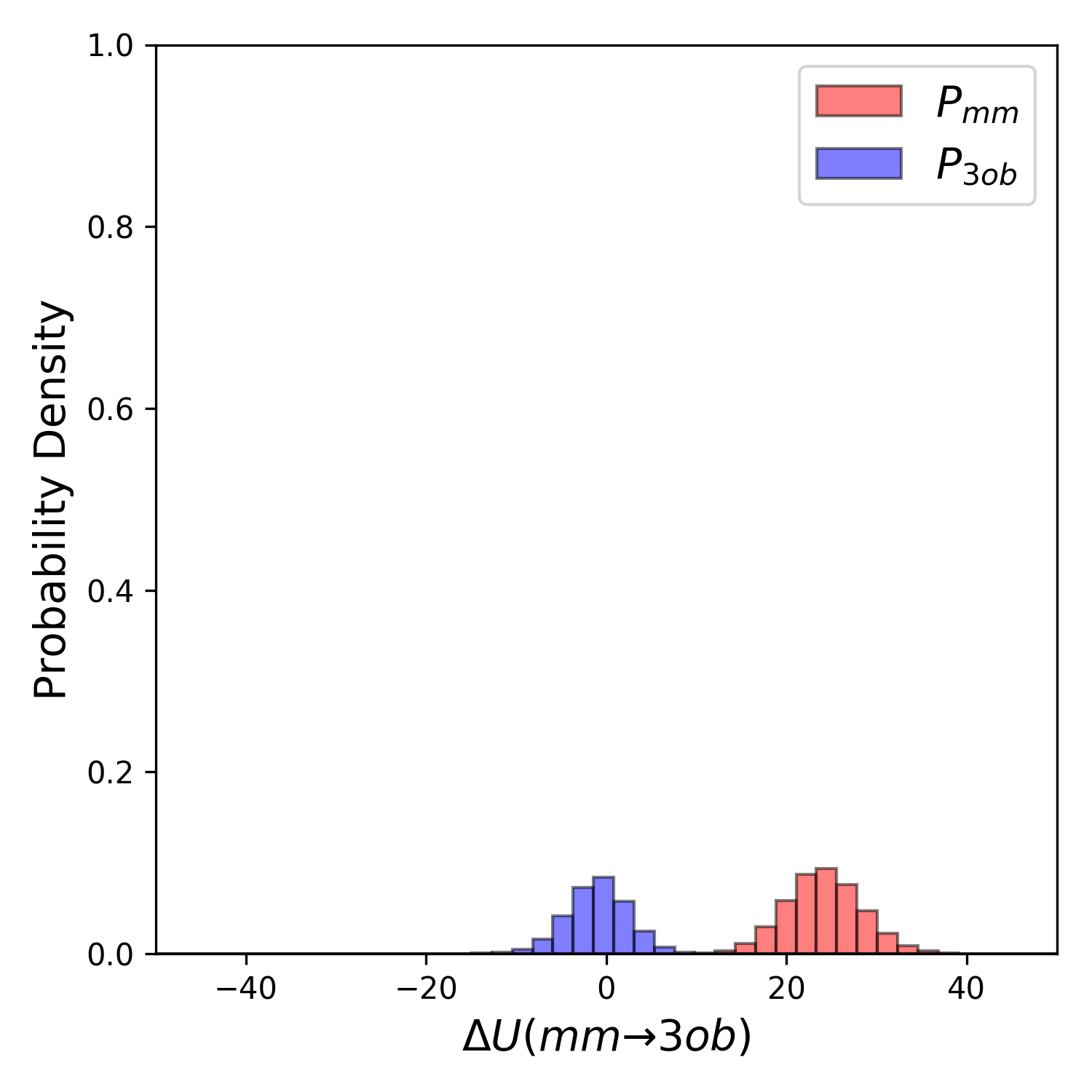

Supplement: Supplementary file 1 [file molecules-24-00681-s001.zip › final-SI/images/zinc_33381936-mm-3ob-fep-overlap.png]

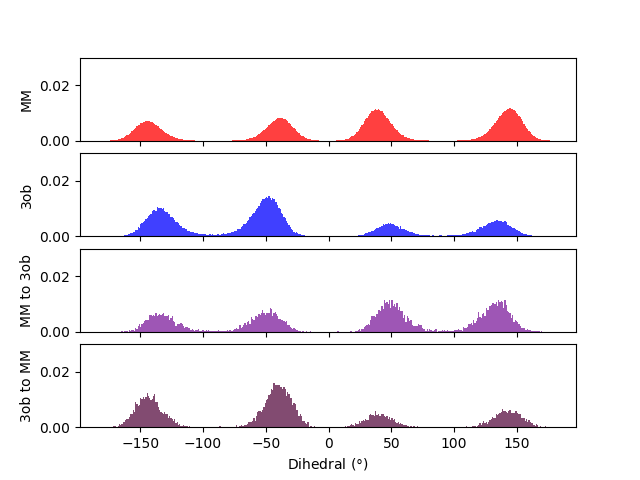

Supplement: Supplementary file 1 [file molecules-24-00681-s001.zip › final-SI/images/zinc_01867000-mm-3ob-subplots-dihe-chi1.png]

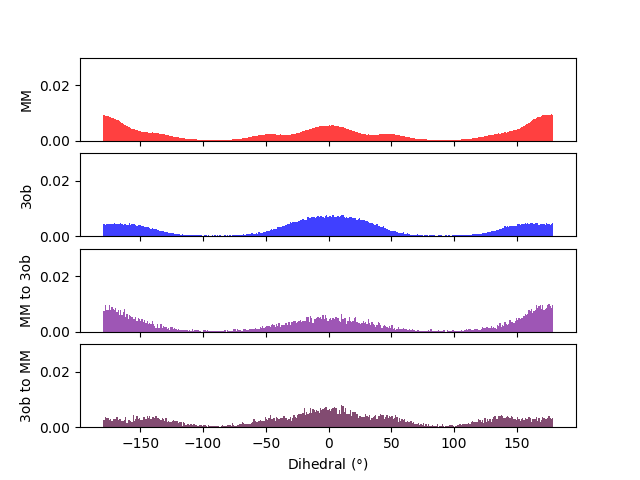

Supplement: Supplementary file 1 [file molecules-24-00681-s001.zip › final-SI/images/zinc_03127671-mm-3ob-subplots-dihe-chi10.png]

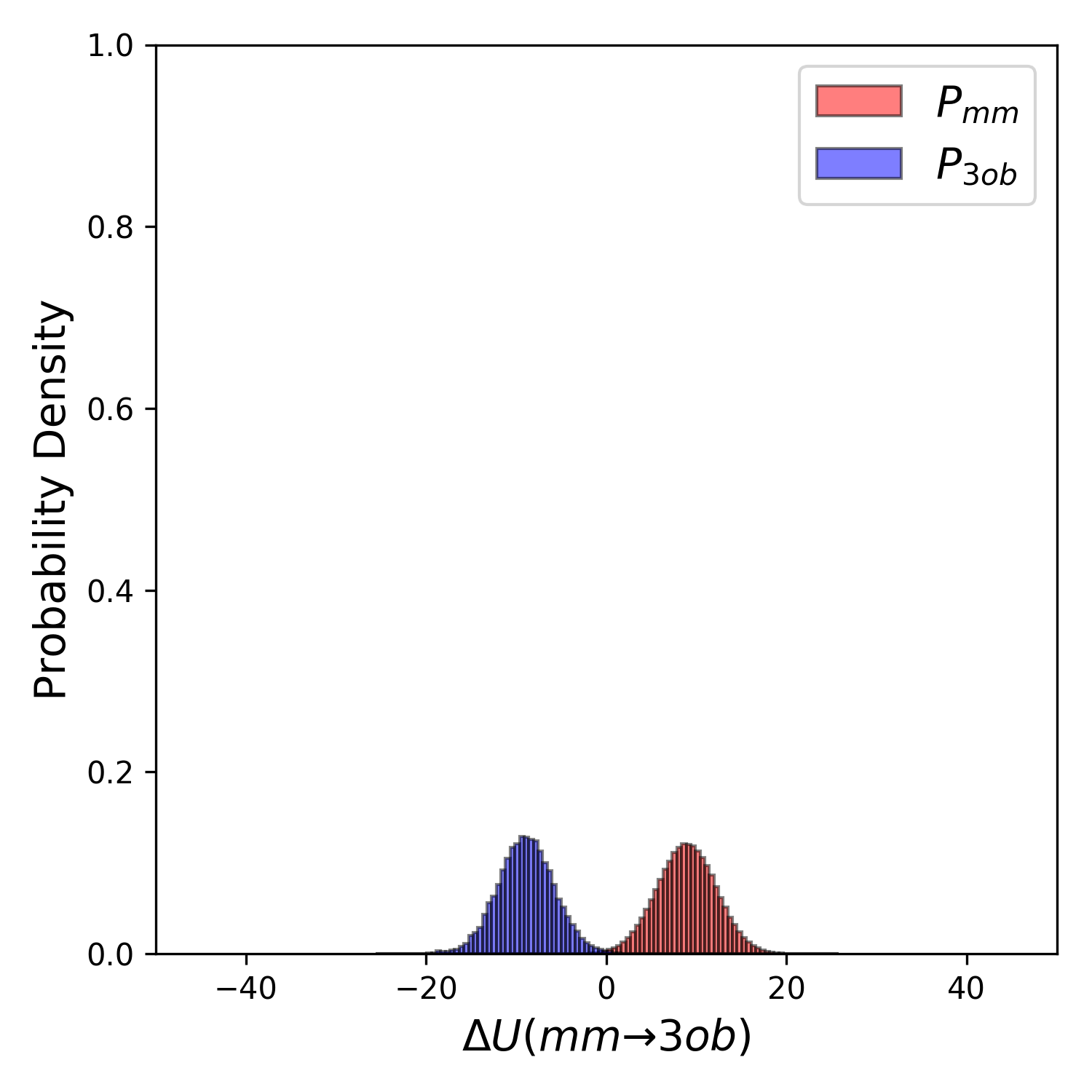

Supplement: Supplementary file 1 [file molecules-24-00681-s001.zip › final-SI/images/zinc_01867000-mm-3ob-fep-overlap.png]

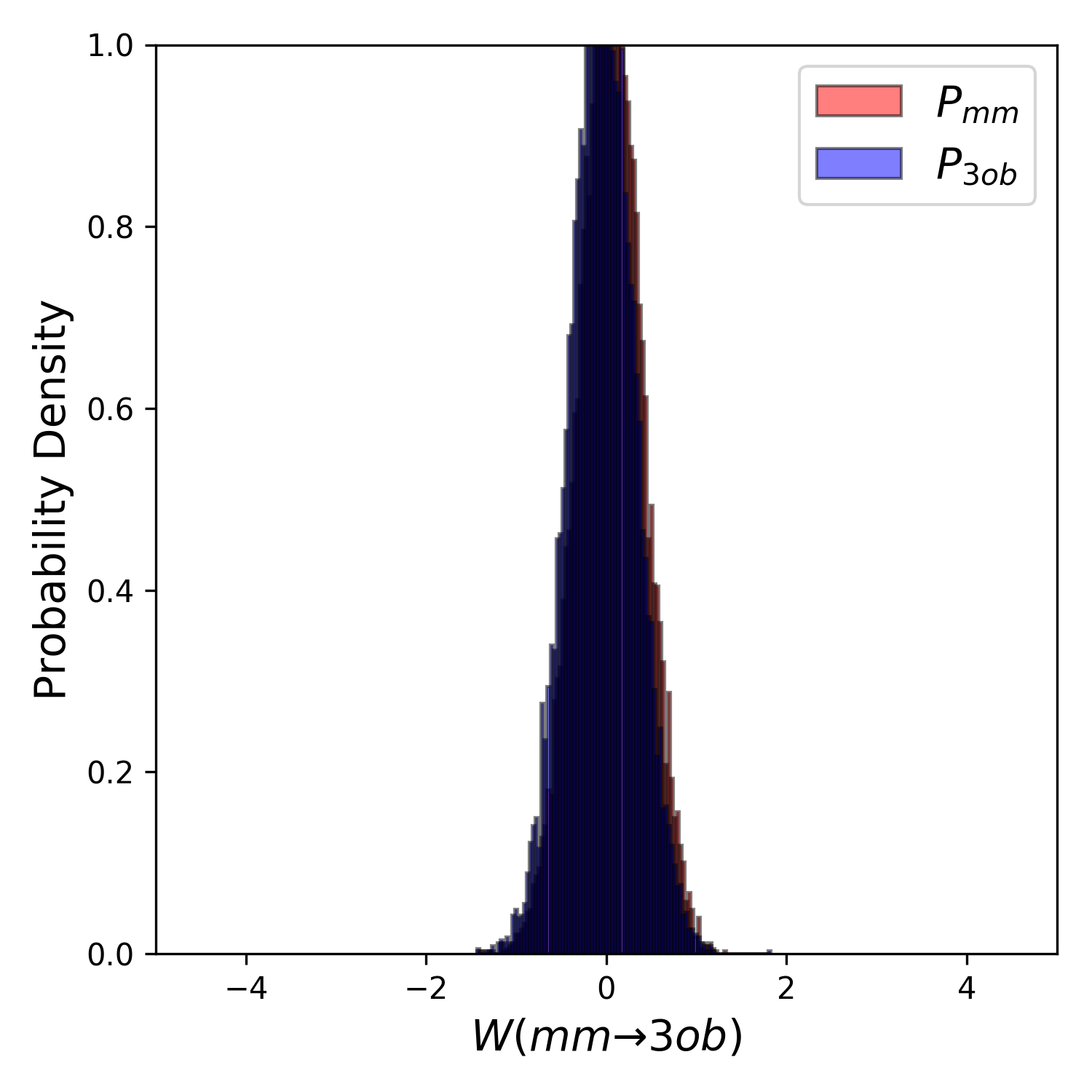

Supplement: Supplementary file 1 [file molecules-24-00681-s001.zip › final-SI/images/zinc_00164361-mm-3ob-jar-overlap.png]

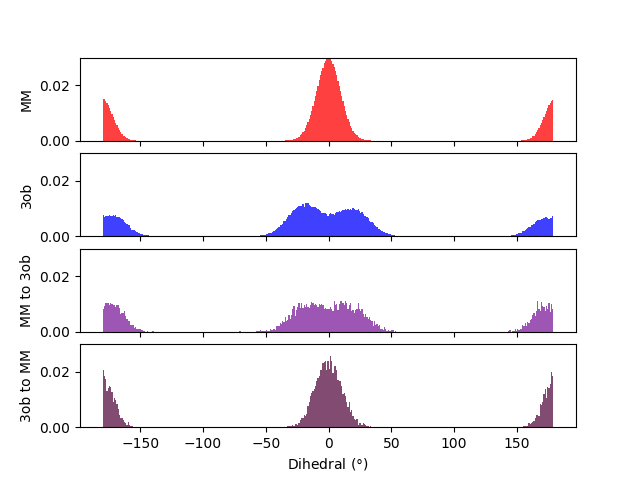

Supplement: Supplementary file 1 [file molecules-24-00681-s001.zip › final-SI/images/zinc_01755198-mm-3ob-subplots-dihe-chi5.png]

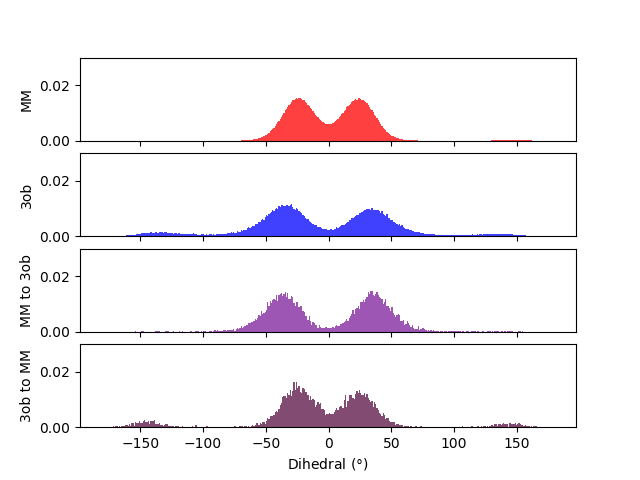

Supplement: Supplementary file 1 [file molecules-24-00681-s001.zip › final-SI/images/zinc_00133435-mm-3ob-subplots-dihe-chi2.png]
